# Capsaicin-Inspired Hydroxamate Hybrids as Selective HDAC6 Inhibitors with Antiproliferative Activity in Hematological Malignancies

**DOI:** 10.1021/acsomega.5c11286

**Published:** 2026-01-28

**Authors:** Lara Gimenez Borges, Thais Nascimento de Oliveira Alves, Sandra Valeria Vassiliades, Jorge Antonio Elias Godoy Carlos, Karoline de Barros Waitman, Sebastian Hilscher, Mike Schutkowski, Wolfgang Sippl, Maurício Temotheo Tavares, Monica Franco Zannini Junqueira Toledo, Letícia Veras Costa-Lotufo, Thales Kronenberger, João Agostinho Machado-Neto, Roberto Parise-Filho

**Affiliations:** a Department of Pharmacy, Faculty of Pharmaceutical Sciences, University of São Paulo, São Paulo 05508-900, Brazil; b Department of Pharmacology, Institute of Biomedical Sciences, University of São Paulo, São Paulo 05508-900, Brazil; c Faculty of Biosciences, Martin-Luther-University of Halle-Wittenberg, Halle/Saale 06120, Germany; d Department of Cancer Biology, 1855 Dana-Farber Cancer Institute, Boston, Massachusetts 02215, United States; e Department of Biological Chemistry and Molecular Pharmacology, Harvard Medical School, Boston, Massachusetts 02215, United States; f Interfaculty Institute of Microbiology and Infection Medicine (IMIT), University of Tübingen, Tübingen 72076, Germany; g German Center for Infection Research (DZIF), Tübingen 72076, Germany; h School of Pharmacy, Faculty of Health Sciences, University of Eastern Finland, P.O. Box 1627, Kuopio FI-70211, Finland

## Abstract

A series of capsaicin-inspired benzodioxol-benzyl-hydroxamate hybrids was synthesized in three steps with high purity (≥95%) and excellent yields (71–94%). Compounds **7a** and **7c** exhibited the strongest antiproliferative effects against Jurkat, Namalwa, and K-562 cells (IC_50_ = 3.0–4.5 μM). Enzymatic assays revealed potent and selective HDAC6 inhibition in the nanomolar range (IC_5__0_ = 0.040 μM ± 0.011 for **7a** and 0.007 μM ± 0.001 for **7c**), with over 300- and 1600-fold selectivity versus HDAC1, respectively. Western blot confirmed target engagement through α-tubulin hyperacetylation, while molecular docking and dynamics studies supported stable bidentate zinc coordination and favorable hydrophobic interactions in the HDAC6 active site. Computational ADMET analyses further supported the experimental findings. These findings identify **7a** and **7c** as potent and selective HDAC6 inhibitors with antiproliferative activity in hematologic tumor cells, highlighting benzodioxol-benzyl hydroxamate hybrids as promising scaffolds for anticancer drug development.

## Graphical abstract

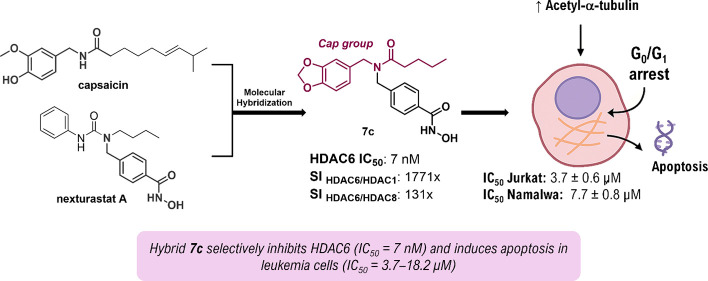

## 1 Introduction

Hematological cancers, including leukemia, lymphoma, and multiple myeloma, represent a major global health burden, with approximately 1.3 million new cases reported in 2019.^1^ Cancer progression is marked by pronounced heterogeneity, giving rise to genetically distinct subpopulations that contribute to variable molecular signatures, treatment responses, and therapeutic resistance.^2,3^ Minimal residual disease can drive relapse linked to epigenetic dysregulation.^4,5^ Among epigenetic mechanisms, histone modifications (*e.g.,* acetylation and methylation) are pivotal in modulating chromatin structure and gene transcription.^6–9^ Histone acetylation is balanced by histone acetyltransferases (HATs) and histone deacetylases (HDACs), enzymes that also target nonhistone proteins. HDACs are classified into zinc-dependent classes I (1,2, 3, 8), IIa (4, 5, 7, 9), IIb (6, 10), and IV (11), and NAD^+^-dependent class III (sirtuins, SIRT1–7), with individual isoforms displaying distinct biological functions.^10^

Among them, HDAC6 attracted particular interest due to its cytosolic activity, regulating cytoskeletal dynamics, cell migration, division, and angiogenesis. HDAC6 dysregulation is associated with immunological and neurological disorders.^8,11–13^ In hematological cancers, HDAC6 overexpression is linked to B-cell, T-cell, Hodgkin’s lymphomas, and lymphocytic leukemia, positioning it as a promising therapeutic target.^14–16^ Moreover, HDAC-mediated deacetylation contributes to inactivation of tumor suppressor genes, strengthening the inhibition relevance.^17–22^

Therefore, HDAC inhibitors (HDACi) development rapidly advanced over the last two decades, with multiple compounds in clinical use and many under investigation (Figure 1).^23^ Vorinostat, approved for cutaneous T-cell lymphoma (CTCL), alongside romidepsin and belinostat for peripheral T-cell lymphomas, demonstrates the broad applicability of these agents.^24^ The approval of panobinostat for multiple myeloma emphasizes its significance in hematological malignancies.^25^ Recently, tucidinostat (i.e., chidamide) was approved for relapsed or refractory peripheral T-cell leukemia/lymphomas, representing the first orally bioavailable benzamide HDACi (Figure 1A).^26^ While some inhibitors lack selectivity among class I isoforms (HDAC1/2/3), resulting in adverse effects, ongoing studies with ricolinostat and citarinostat in therapeutic combinations offer promising prospects^27–29^ (Figure 1B). Selective HDAC6 inhibitors, such as tubastatin A (Figure 1B) and nexturastat A (**1**, Figure 1C), are emerging as innovative treatment strategies.^30,31^ Nexturastat A **1** is effective in hematological neoplasms and myeloma, inhibiting tumor growth and inducing apoptosis.^32–34^

**Figure 1:**
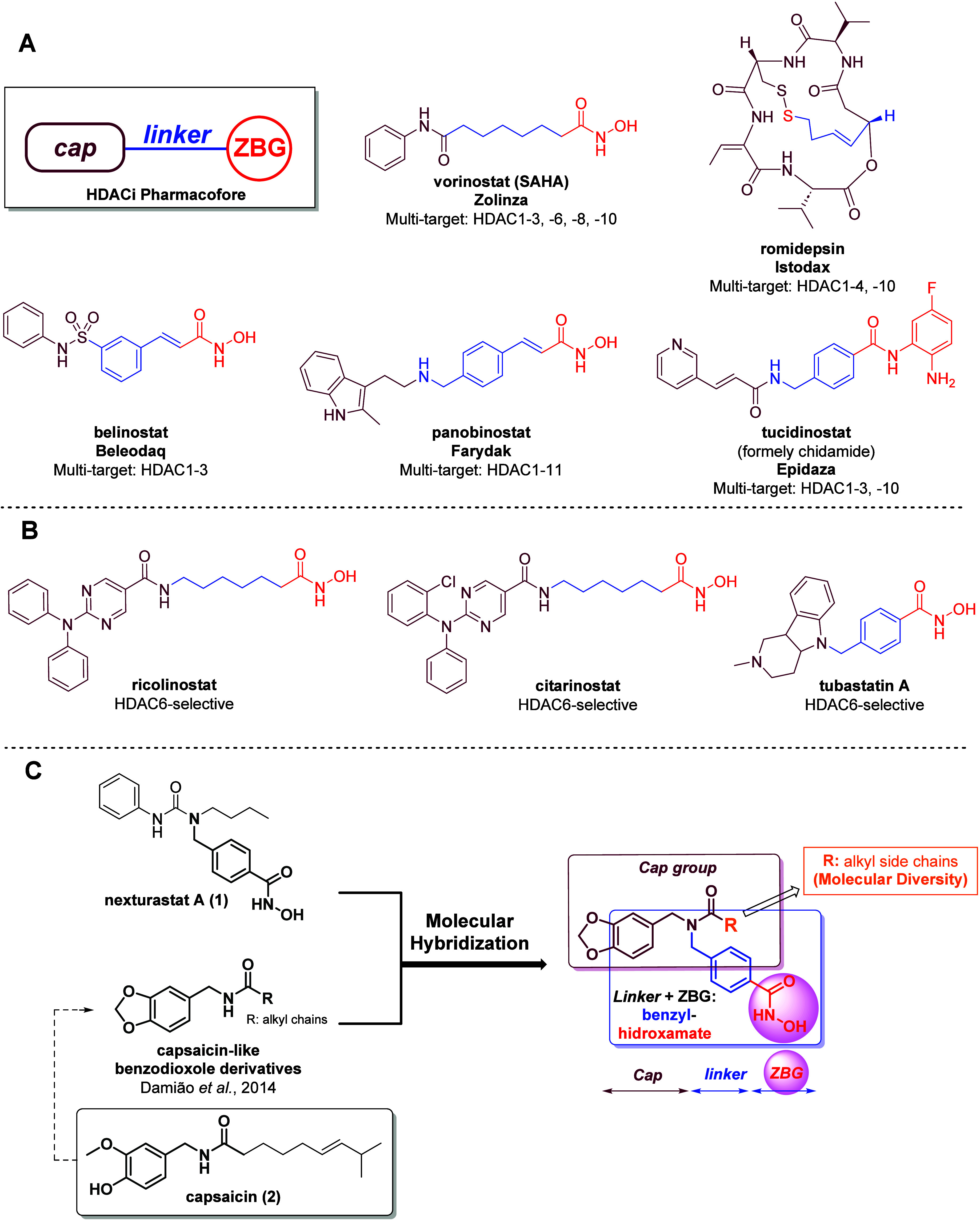
A) Pharmacophoric model of HDACis and chemical structures of the approved HDACis. B) HDAC6-selective inhibitors under investigation. C) Design of benzodioxol-benzyl-hydroxamate derivatives by molecular hybridization. Original figure created for this study.

HDAC’s pharmacophore consists of three interconnected structural portions, namely: (i) a zinc binding group (ZBG), which interacts with the catalytic Zn^2+^; (ii) a linker, that occupies the narrow tunnel leading to the cavity; and (iii) the or cap, which lies on the catalytic cavity’s surface (Figure 1A).^35^ Notably, HDAC6’s cavity surface can accommodate a diverse array of caps due to its inherent flexibility.^35^ Conversely, modifications in the compound’s ZGB and linker significantly impact HDAC6 inhibition, highgliting the role of molecular design to maintain optimal interactions.^35,36^

To expand chemical diversity within the cap region, natural products have long served as prime sources of inspiration.^38^ Beyond direct HDAC inhibitors, natural scaffolds with established anticancer activity contribute to structural innovation. Among those, vanilloid phytochemicals from *Capsicum* species – especially capsaicin (**2**, Figure 1C) – stand out. Capsaicin exhibits anti-inflammatory, metabolic, and broad anticancer properties across colorectal, breast, prostate, gastric, and hematological malignancies.^39–47^ Despite this versatility, its clinical use is limited by low solubility and adverse effects,^46^ prompting efforts to optimize this scaffold. A key advancement lies on the vanillyl group substitution with a 1,3-benzodioxole ring, markedly enhancing cytotoxicity in tumor cell lines.^48,49^ Our group further demonstrated that benzodioxole-containing capsaicin analogues show activity in mammary cancer, glioma, melanoma, prostate cancer, and hematological cancer cells, supporting its role as a privileged scaffold for anticancer drug design.^37,45,46,48^

Building on the therapeutic relevance of HDAC6 inhibitors and their frequent synergy with other anticancer agents, we pursued a hybridization strategy combining structural elements of nexturastat A (**1**) with those of capsaicin (**2**), incorporating the benzodioxole unit shared with potent agents such as podophyllotoxin derivatives (Figure 1C).^50–52^ Additional diversity was introduced via systematic variations of of alkyl side chains length and composition.^53^

The resulting hybrids were synthesized and evaluated for cytotoxic in tumorigenic and nontumorigenic cell lines, with emphasis on HDAC6 inhibition as the intended molecular target. Molecular modeling complemented the biological data, providing insights into the observed SAR patterns.

## 2 Results and Discussion

### 2.1 Chemistry

An efficient and simple three-step approach was employed to obtain the desired compounds. The synthetic route can be seen in Scheme 1.

**Scheme 1:**
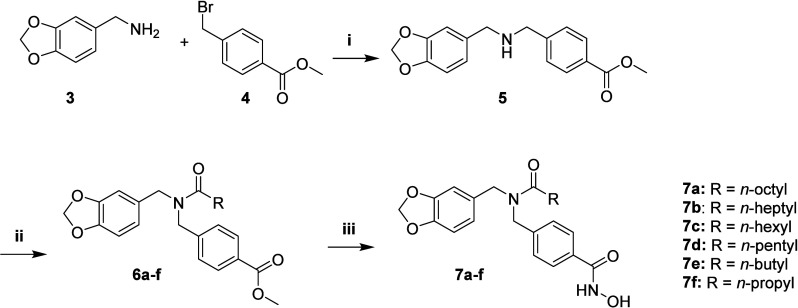
Synthetic Proposal for Compounds **7a**–**f** ^a^

a Reagents and conditions: (i) THF, reflux, N_2_.; (ii) RCOCl, Et_3_N, DCM, N_2_, r.t.; (iii) NH_2_OH, NaOH, THF/MeOH (1:1), 0°C > r.t

In the first step, the key intermediate **5** was synthesized through an alkylation reaction between piperonylamine (**3**) and methyl 4-(bromomethyl)­benzoate (**4**), affording a yield of 87%. This compound had previously been obtained by Nagaoka et al. (2006) in 33% yield,^54^ demonstrating the substantially higher efficiency of our protocol. Subsequently, acylation of intermediate **5** with *n*-alkyl acyl chlorides was carried out in dichloromethane using triethylamine as base. Reactions performed at room temperature favored the reactivity of the acyl chlorides, leading to intermediates **6a–f** in 62–97% yields. The lower yield observed for the butanoyl derivative (**6f**, 62%) is attributed to the higher volatility and greater susceptibility to hydrolysis of short-chain acyl chlorides, whereas longer-chain analogues exhibit improved solubility and reactivity under the same conditions, resulting in higher isolated yields. In the final step, hydroxylaminolysis of intermediates **6a–f** with aqueous hydroxylamine solution (50% w/v) at 0 °C furnished the new hydroxamic acid derivatives **7a–f** in 71–94% yields. Precipitation with cold water followed by recrystallization from ethanol afforded analytically pure products (≥95% HPLC).^55^

### 2.2 Capsaicin-Derived Hybrids Inhibited the Proliferation of Hematological Cancer Cell Lines

The cytotoxic potential of the final compounds **7a–f** was assessed by MTT assay.^56^ Because HDAC6 inhibitors often display enhanced activity in hematological malignancies, the hybrids were evaluated in clinically relevant leukemia models: Jurkat (T-cell acute lymphoblastic leukemia), Namalwa (Burkitt lymphoma, B-cell lineage), and K-562 (chronic myeloid leukemia). These models allow comparative assessment of compound activity across T-, B-, and myeloid leukemia backgrounds. To address selectivity, the compounds were also tested in nontumorigenic cell lines, including HS5 (human fibroblasts), CCD18CO (human colon), and HaCaT (skin keratinocytes).^17–20^ For comparative purposes, vorinostat (a pan-HDAC inhibitor), nexturastat A **1** (prototype and selective HDAC6 inhibitor), and capsaicin **2** (natural scaffold of the designed hybrids) were included as reference standards. At a fixed micromolar concentration, all compounds demonstrated measurable cytotoxicity in hematological models, with differential responses among the tested lineages.

Compounds **7a–f** exhibited cytotoxic activity in the micromolar range, with the lowest IC_50_ values observed in the Jurkat lineage (3.7–10.2 μM), followed by Namalwa cells (7.7–14.8 μM), while K-562 cells were comparatively less sensitive (18–34 μM) (Table 1). Notably, compound **7a** stood out for its strong activity and selectivity in K-562 cells relative to the other hybrids. Additionally, the three most active compounds in all studied cell lines were **7a**, **7c**, and **7e**, with compounds **7b** and **7f** showing lower IC_50_ values for two cell lines, Jurkat and Namalwa.

**Table 1:**
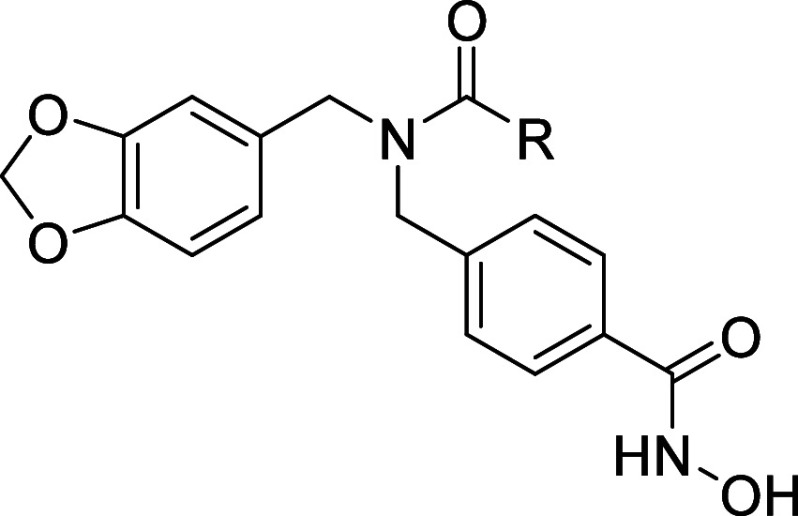

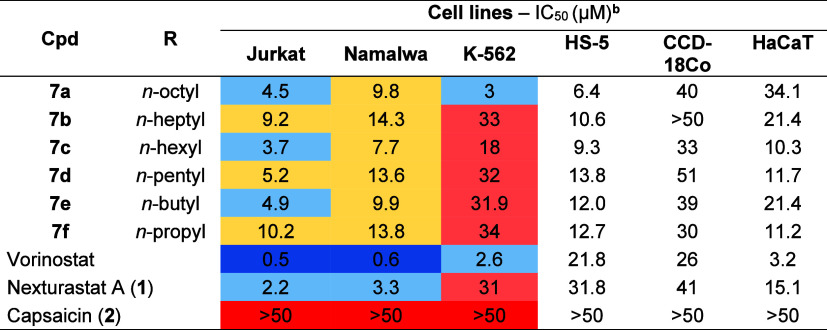
Cytotoxicity of Compounds **7a**–**f** against Hematological Tumor Cells^a^

a Values obtained by MTT bioreduction assay.

b *n* = 2, meaning two independent experiments performed in duplicates. The values are represented by a gradient from green to red to indicate compounds′ potency: very potent (IC_50_ ≤ 1 μM, dark blue), high potency (1 μM < IC_50_ ≤ 5 μM, light blue), moderate (5 μM < IC_50_ < 15 μM, yellow), low potency (15 μM ≤ IC_50_ < 50 μM, red), and inactive (IC_50_ ≥ 50 μM, dark red).

All synthesized compounds showed IC_50_ values above 10 μM in HaCaT cells, indicating preferential selectivity for malignant over nontumorigenic skin cells. In contrast, lower IC_50_ values were observed in HS5 fibroblasts, suggesting reduced selectivity in this lineage and highlighting the need for further optimization. For CCD18CO colon cells, the compounds consistently exhibited high IC_50_ values, with nexturastat A (**1**) serving as a reference (41 μM). Overall, the hybrids displayed higher IC_50_ values than vorinostat and slightly lower than nexturastat A (**1**), except for **7a** and **7d**, which showed values close to or higher than their parent compound. These results support that the synthesized compounds retain good cytotoxic activity in tumorigenic cells while maintaining favorable selectivity against most nontumorigenic models.

When compared with the pan-HDAC inhibitor vorinostat, compounds **7a–f** showed lower potency, which is consistent with the expected profile of isoform-selective inhibitors.^57^ Relative to nexturastat A (**1**), the hybrids generally displayed higher IC_50_ values, although some (notably **7a** and **7c**) approached their activity in specific cell lines. As anticipated, all hybrids were markedly more active than capsaicin **2**, since, although inspired by this natural scaffold, they were rationally designed to act as HDAC6 inhibitors and may therefore trigger cell death through a distinct mechanism.

Selectivity analysis highlighted a preferential activity against tumorigenic cells. Compounds **7a** and **7c**, the most active hybrids, showed particularly high selectivity indexes against CCD18Co (8.9 and 4.3, respectively), while maintaining moderate selectivity over HaCaT and HS5 cells. Importantly, compound **7a** combined potent activity in K-562 cells with superior selectivity compared to **7c**, reinforcing its potential as the most balanced candidate in the series.

Finally, no clear structure–activity relationship (SAR) was observed concerning acyl chain length or lipophilicity. Nevertheless, the data suggest that side chains containing five to seven carbon atoms tend to favor cytotoxicity. Notably, compound **7a** (n-octyl) exhibited lower IC_5__0_ values than **7c** (n-hexyl) in Jurkat, Namalwa, and K-562 cell lines, although with a slightly reduced selectivity profile, highlighting the delicate balance between potency and selectivity in this series.

### 2.3 Compounds **7a** and **7c** Promote Cell Cycle Arrest and Induce Apoptosis

To elucidate the cytotoxic mechanisms associated with HDAC inhibition in different hematological cell lines, compounds **7a** and **7c** were further evaluated for their ability to induce cell death in comparison with controls **1**, **2**, and vorinostat. To specifically investigate their impact on apoptosis, Annexin V-positive cells were quantified following treatment with full and half IC_50_ concentrations (Figure 2 and Supporting Information, Figures S1–S3).

**Figure 2:**
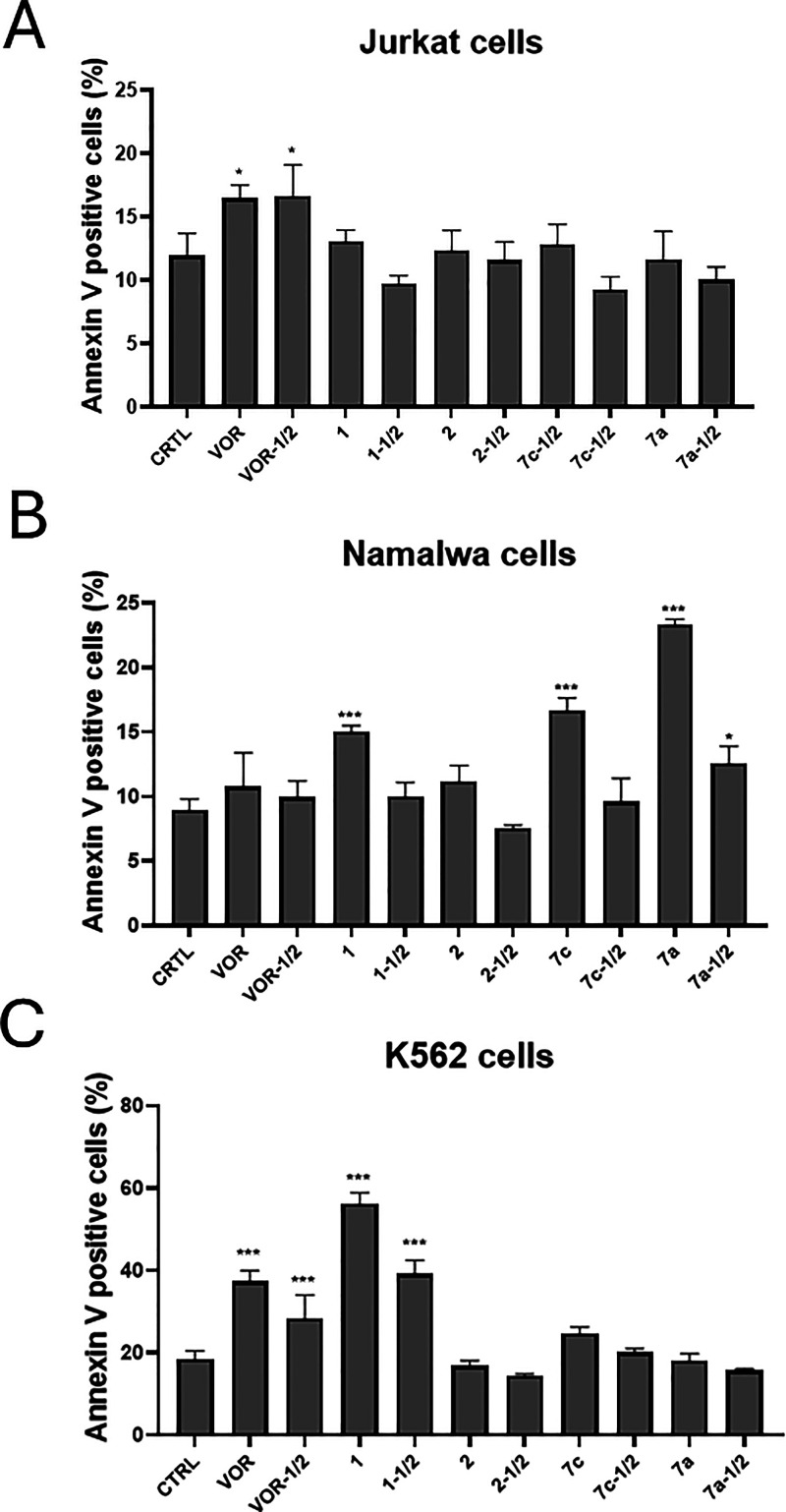
Quantification of Annexin V-positive Jurkat (**A**), Namalwa (**B**), and K-562 (**C**) cells under different treatment conditions. Bar graphs represent the mean ± SD of three independent experiments (*n* = 3). Statistical significance was determined by one-way ANOVA followed by Bonferroni post-test (*p < 0.05; **p < 0.01; ***p < 0.001). VOR: vorinostat.

In Jurkat cells, only vorinostat significantly increased Annexin V-positive cells, consistent with apoptosis induction (Supporting Information, Figure S1). In Namalwa cells, compounds **7a** and **7c** exhibited a cell death profile comparable to that of the selective inhibitor **1**, with apoptosis occurring predominantly through late apoptosis (Q2), followed by early apoptosis (Q3) (Supporting Information, Figure S2). At 50 μM, both compounds induced higher levels of apoptosis than standards **1**, **2**, and vorinostat, with **7a** nearly doubling the proportion of apoptotic cells. These results suggest greater efficacy and the possibility of a more favorable therapeutic window, although single-dose experiments do not allow definitive conclusions regarding dose–response and selectivity, warranting further investigation. In contrast, in K-562 cells, only compound **1** and vorinostat consistently induced apoptosis (Supporting Information, Figure S3). Compounds **7a** and **7c** produced apoptosis rates comparable to capsaicin **2** and below those of the controls, indicating that their cytotoxicity in this lineage may occur through nonapoptotic mechanisms.

**Figure 3:**
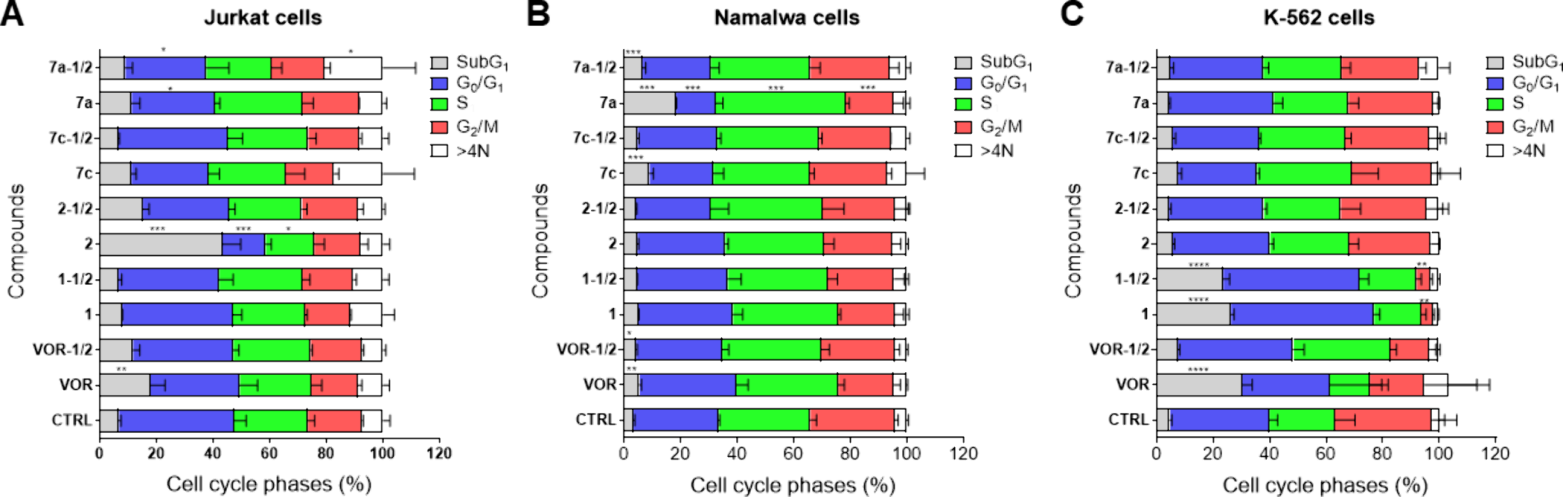
Cell cycle analysis of Jurkat (**A**), Namalwa (**B**), and K-562 (**C**) cells treated with compounds **7a**, **7c**, **1**, **2**, and vorinostat. Stacked bar graphs represent the distribution of cell populations in G0/G1 (blue), S (green), G2/M (red), and sub-G1 (gray) phases. Data are expressed as mean ± SD from at least four independent experiments. Statistical significance was determined by one-way ANOVA followed by Bonferroni post-test (*p < 0.05; **p < 0.01; ***p < 0.001).

Cell cycle distribution was evaluated in Jurkat, Namalwa, and K-562 cells treated with compounds **7a** and **7c**, compared with standards **1**, **2**, and vorinostat (Figure 3). In Jurkat cells (Figure 3A), compounds **1** and **7c** did not alter the cell cycle profile compared to the control. In contrast, compound 7**a** induced cell cycle arrest at the G0/G1 phase, consistent with a cytostatic effect by blocking progression into DNA synthesis. Vorinostat increased the sub-G1 population, typically associated with DNA fragmentation and apoptosis, whereas capsaicin **2** altered the distribution across sub-G1, G0/G1, and S phases, showing a profile more closely related to **7a** than to **1**. In Namalwa cells (Figure 3B), compounds **7a**, **7c**, and vorinostat promoted cell accumulation in the sub-G1 phase, while compounds **1** and **2** did not significantly affect the distribution. In K-562 cells (Figure 3C), cell cycle changes were observed only for the standard compounds **1** and vorinostat. Compound **1** increased sub-G1 and G2/M populations, suggesting cell death associated with these phases, whereas vorinostat mainly induced sub-G1 accumulation. Compounds **7a** and **7c** did not significantly modify the distribution in this lineage.

**Figure 4:**
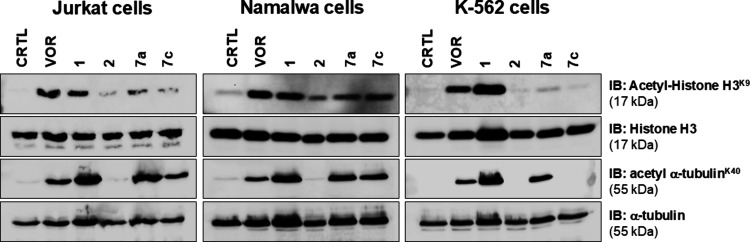
Effect of the HDAC inhibitors on specific acetylated substrates. Immunoblotting of acetyl-α-tubulin, α-tubulin, acetyl-histone H3, and histone H3 proteins in hematologic cancer cell lines (Jurkat, Namalwa, and K-562) treated for 24 h with vorinostat (VOR), Nexturastat A (1), capsaicin (2), 7a, and 7c at their respective 72-h TGI concentrations. For compounds with undetermined TGIs, a fixed concentration of 50 μM was used.

### 2.4 The Antiproliferative Effects of **7a** and **7c** are Linked to HDACs Inhibition

The biochemical evaluation of inhibitory activity against HDAC1, −6, and −8 was performed in comparison with our three reference compounds: vorinostat, nexturastat A (**1**), and capsaicin **2** (Table 2). This investigation aimed to assess the compounds’ selectivity or determine their potential as pan-HDAC inhibitors. It is noteworthy that assays on these three isoforms provide crucial data that can reveal activity and specificity patterns.

**Table 2:** Enzymatic Inhibition of HDACs^a^

	**Enzymatic Inhibition** – IC_50_ (μM ± SD)						
Compound	HDAC1		HDAC6		HDAC8	SI^a^	
**7a**	12.900 ± 0.001		0.040 ± 0.011		1.787 ± 0.253	322.5	
**7b**	14.497 ± 0.002		0.025 ± 0.003		1.070 ± 0.002	579.88	
**7c**	12.400 ± 0.001		0.007 ± 0.001		0.920 ± 0.046	1771.43	
**7d**	1.769 ± 0.002		0.005 ± 0.002		0.122 ± 0.002	93.10	
**7e**	7.276 ± 0.002		0.019 ± 0.003		0.927 ± 0.002	406.63	
**7f**	4.231 ± 0.002		0.033 ± 0.003		1.094 ± 0.002	128.21	
**VOR**	0.100 ± 0.007		0.033 ± 0.003		0.420 ± 0.080	3.03	
**1**	3.176 ± 0.131		0.001 ± 0.0001		0.553 ± 0.032	3176.00	
**2**	>20		>20		>20	-	

a Legend: The table presents the mean IC_50_ values (μM ± SD) obtained from *in vitro* enzymatic inhibition assays for compounds **7a–7f, VOR, 1,** and **2** against recombinant human histone deacetylase (HDAC) isoforms of classes I (HDAC1 and HDAC8) and IIb (HDAC6). IC_50_ (μM) represents the inhibitor concentration required to reduce enzyme activity by 50%, with lower values indicating greater inhibitory potency. Data are expressed as the mean ± standard deviation (SD) from at least three independent experiments. Values reported as “>20 μM” correspond to inhibition below the detection limit of the assay. ^*a*^ *SI* (HDAC1/HDAC6 Selectivity Index) denotes the ratio between the IC_50_ values for HDAC1 and HDAC6, where higher values indicate greater relative selectivity for HDAC6 over HDAC1.

Based on the IC_50_ values presented in Table 2, it is evident that the compounds **7a**–**f** exhibit moderate to high activity across the tested isoforms, with strong inhibitory capacity in the nanomolar range for HDAC6. In this isoform, the compounds displayed comparable potency to nexturastat A (**1**), a selective HDAC6 inhibitor, with compounds **7a, 7b,** and **7c** demonstrating the highest HDAC6-selectivity. Compound **7a** is approximately 322 times more selective for HDAC6 than HDAC1 and about 44 times more selective for HDAC6 than HDAC8. While compound **7b**, demonstrates approximately 580-fold selectivity over HDAC1 and 43-fold selectivity over HDAC8. Compound **7c**, on the other hand, is approximately 1771 times more selective for HDAC6 than HDAC1 and exhibits ∼ 131 times more selectivity for HDAC6 compared to HDAC8. This pattern of selectivity is pharmacologically meaningful, as avoiding inhibition of class I HDAC isoforms (HDAC 1/2/3) is essential to minimize the hematologic and gastrointestinal toxicities typically associated with pan-HDAC inhibitors, whereas selective targeting of HDAC6 is linked to improved tolerability and a more favorable therapeutic window. The results of the compounds **7a** and **7c** align with the data obtained from cytotoxicity assays, suggesting that inhibition of HDAC6 may contribute to cell death in hematologic tumor cell lines. This is particularly relevant considering that nexturastat A (**1**) itself induces apoptosis in human multiple myeloma cells.^58^

Nonetheless, when comparing the enzymatic inhibition results of compound **7b** with cytotoxicity assays, a slight discrepancy is observed. While it demonstrates good enzymatic inhibition activity, compound **7b** exhibits one of the highest IC_50_ values in the cellular cytotoxicity assay, trailing only compound **7f**. Therefore, despite its selectivity against HDAC6, it does not show as pronounced efficacy against tumorigenic activity in hematologic tumor cell lines compared to compounds **7a** and **7c**.

It is also important to evaluate the results obtained for compounds **7d**, **7e**, and **7f**. All of them showed good IC_50_ values for HDAC6 enzymatic inhibition; however, they did not exhibit high selectivity compared to other isoforms. This suggests that the cell death observed in the tested hematologic tumor cell lines may result from the inhibition of both HDAC6 and one other HDAC isoform. In particular, compound **7d** has the lowest IC_50_ value for HDAC6 enzymatic activity, but its lower IC_50_ values for other isoforms indicate it is not as selective as compounds **7a**, **7b**, and **7c.**

However, it is worth noting that among the three compounds that presented the best selectivity for HDAC6, **7a**–**c**, the latter was the most active across all three evaluated isoforms. Additionally, compound **7c** exhibited an IC_50_ of 7.7 nM, a value close to the IC_50_ of its standard and prototype, compound **1**. This result is consistent with the IC_50_ values obtained from cytotoxicity assays, where **7c** showed the best results compared to other compounds (except for the K-562 cell line). This reinforces the hypothesis that, in this study, cell death in hematologic cell lines may be related to HDAC6 inhibition.

However, cell death may not solely occur through HDAC6 inhibition, as there are studies on the mechanism of cell death induced by nexturastat A (**1**) in multiple myeloma cell lines, where compound **1** was found to suppress cell growth by arresting the cell cycle in the G1 phase through CDK2 activity reduction. Additionally, another mechanism of cell death induced by **1** involves the activation of caspases 3 and 9, which are crucial proteases for apoptosis activation.^34^

To link the antiproliferative effects with HDAC inhibition, the modulation of acetylated proteins was evaluated in three hematological cell lines exposed to the selected HDAC inhibitors **7a** and **7c** and to the respective controls, at their tumor growth inhibition (IC_50_, 72 h) concentrations, using Western blot (Figure 4). These compounds were chosen for mechanistic cellular assays because they exhibited the most promising enzymatic inhibition profiles in the biochemical screening, particularly toward HDAC1 and HDAC6, respectively. This rationale strengthens the connection between enzymatic data and the cellular validation, ensuring that the observed modulation of acetylated histone H3 and α-tubulin reflects the specific inhibitory properties of each compound rather than arbitrary selection. The accumulation of both acetylated-H3 and acetylated α-tubulin was determined as readout for HDAC activity. Assays were performed with acetyl-histone-H3 and acetyl-α-tubulin, with the former predominantly associated with HDAC1 and the latter with HDAC6. For inhibitors whose IC_50_ concentration could not be determined, a concentration of 50 μM was applied.^59,60^

**Figure 5:**
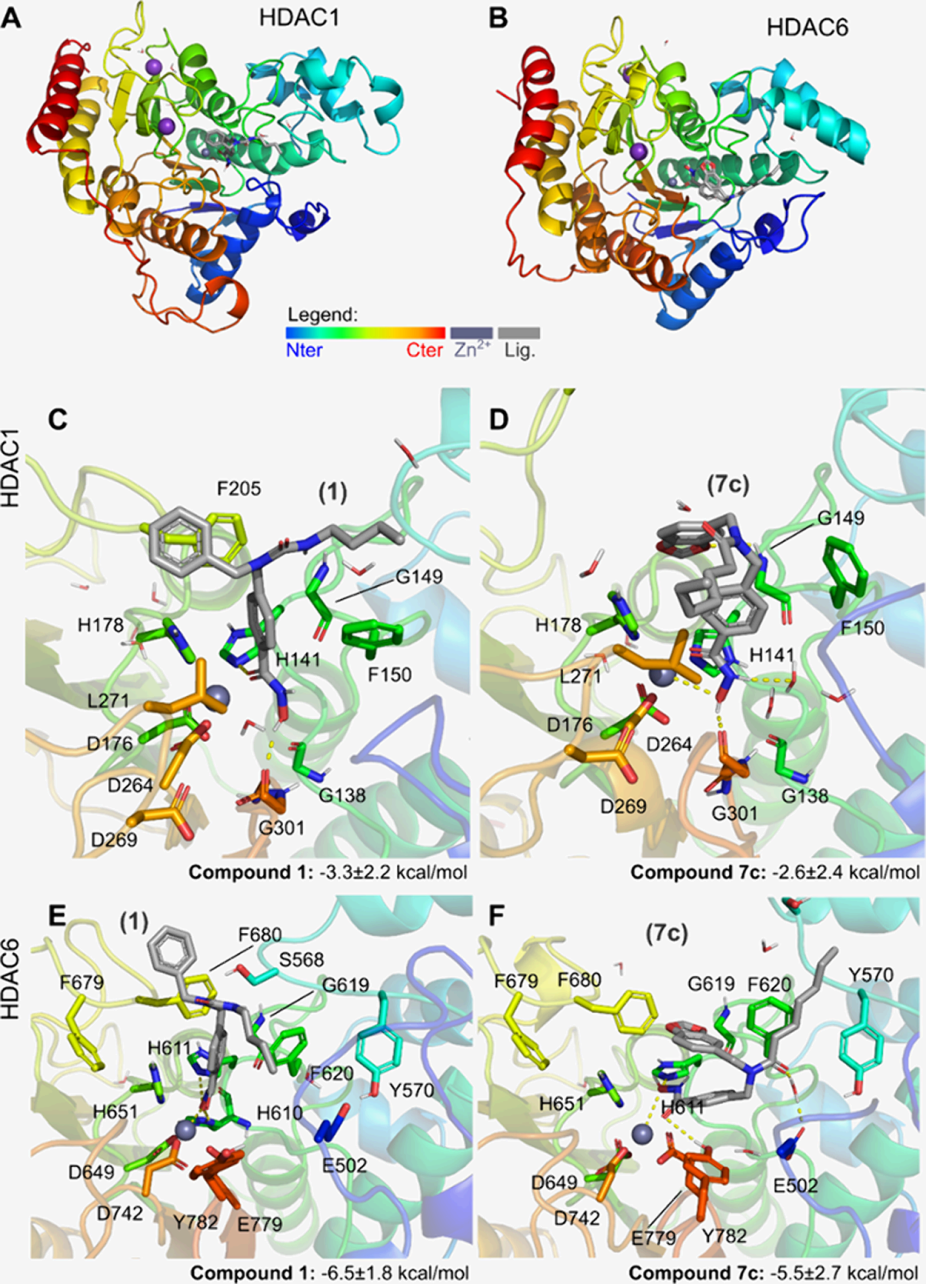
(A,B) overview of human HDAC1 (A, PDB 5ICN) and HDAC6 (B, modeled after PDB 5EDU). Potential binding mode for relevant HDAC inhibitors within this binding site for: compound **1** (C,E) and **7c** (D,F), retrieved by the most populated cluster during the simulation (see Supp. Information for the methods and information on other clusters). Predicted binding energies by the means of ligand efficiency, calculated along the simulation trajectories (5 × 500 ns) are depicted below the respective binding modes.

In this sense, when observing the Jurkat and Namalwa lineages, it is notable that compounds **7a** and **7c** exhibit low staining for acetyl-histone-H3, indicating that there is not a predominant inhibition of HDAC1 and that deacetylation of this protein continues to occur, unlike what is observed for standards vorinostat and **1**. Meanwhile, staining is observed in the wells for acetyl-α-tubulin, indicating that deacetylation of this protein is not occurring and that compounds **7a** and **7c** are likely inhibiting HDAC6. It is important to emphasize that HDAC6 is the main player of α-tubulin deacetylation,^61^ which is consistent with the potent HDAC6 inhibition by **7a** and **7c**.^62,63^

In the K-562 cell line, the absence of detectable staining for acetylated histone H3 indicates that histone acetylation levels remain very low. Since HDAC1 is the enzyme responsible for removing acetyl groups from histone H3, this result suggests that its deacetylase activity is largely preserved in these cells. Compound **7a** exhibits high staining for acetyl-α-tubulin, indicating strong inhibition of HDAC6. Similar to selective HDAC6 inhibitors such as tubacin, tubastatin A, and Nexturastat A (compound **1**), which markedly increase α-tubulin acetylation without significantly affecting histone acetylation, compound **7a** also induces an increase in α-tubulin acetylation.^64,65^ However, in the case of this latter protein, compound **7c** shows no staining, indicating that cell death caused by this compound likely does not occur through HDAC6 inhibition in this tumorigenic cell line. According to previous studies, compound **7c** probably exhibits a cell death profile similar to that of its parent compound, capsaicin (compound **2**).

Although compound **7c** exhibited the highest enzymatic potency and selectivity for HDAC6, it failed to promote α-tubulin acetylation or induce apoptosis in K-562 cells. Since all analogs share the same hydroxamate zinc-binding group, the difference likely arises from subtle variations in the *n*-alkyl side-chain length, which can modulate membrane partitioning, intracellular accumulation, and productive engagement within the HDAC6 channel. Similar discrepancies between *in vitro* inhibition and cellular efficacy have been reported for other HDAC6 inhibitors, where optimal chain length and physicochemical balance are relevant for converting enzymatic potency into biological response. Therefore, **7c** can be considered a highly potent yet less K-562 cell-active inhibitor, emphasizing the importance of fine-tuning lipophilicity and conformational flexibility during optimization.^66–68^

### 2.5 Compounds **7a** and **7c** Present Favorable Interactions in Their Target HDAC Isoforms

Docking simulations were carried out for all synthesized compounds in HDAC 1/6 and the complete results are available in the Supporting Information (Figures S4–S5, Table S1). Among the series, compounds **7a** and **7c** displayed distinct binding modes of the hydroxamate zinc-binding group (ZBG). Compound **7a** binds Zn^2+^ in a monodentate fashion, resembling the interaction profile reported for nexturastat A (1). In contrast, compound **7c** coordinates Zn^2+^ in a bidentate mode, stabilized by three hydrogen bonds involving residues in the catalytic pocket.^67,69^ For both compounds, the capping groups established hydrophobic contacts with residues His463, Ala641, and Pro464, which contributed to binding stabilization. The stronger interaction observed for **7c** likely explains its higher docking score and aligns with the experimental data, where **7c** showed the most potent enzymatic inhibition and superior selectivity within the series.

Molecular dynamics (MD) simulations were further performed to investigate the binding modes of compound **7c** and reference inhibitor **1** in HDAC1 and HDAC6 model enzymes (Figure 5 and Figure S6, and Table S2). To rationalize the differences in potency across enzymes, binding free energies were estimated using the MM/GBSA approach, and the frequency of protein–ligand interactions was analyzed along the simulation trajectories, as previously described.^70^ For consistency, both compounds were modeled assuming bidentate coordination of the hydroxamate group to the Zn^2+^ ion.

The proposed binding mode of compounds **1** and **7c** within HDAC1 yielded unstable simulations, with the ligands presenting high RMSD values in at least 20–40% of the simulation time (Table S2). This instability is associated with the dynamic rotation of the cap moiety, where the benzodioxole ring and acyl groups continuously flip and switch positions. The linker group is stabilized by transient interactions with His141, Phe150, His178 and Phe205, while the cap weakly interacts with Phe150 and Leu271. These transient interactions, indeed, reflect on a poorly predicted binding free energy of −11.4 kcal/mol for HDAC1-**7c** (Table S2). In contrast, binding of these compounds to HDAC6 is stable during the entire simulated time with the ZBG of **7c** strongly interacting with His611 (44% of the analyzed simulation time), its linker being supported by Phe620 and His651, and, most importantly, its cap meeting a shallow surface pocket with Tyr782 and Tyr570. Those interactions contribute to a stronger predicted binding energy ΔG of −24.2 kcal/mol, largely driven by hydrophobic contributions (Table S2)­.

### 2.6 In Silico ADMET and Pharmacokinetic Predictions

*In silico* ADMET analysis^71^ (Table S3, Supporting Information) revealed physicochemical differences between **7a** and **7c** that help rationalize their divergent cellular activities. Although both compounds display TPSA values appropriate for HDAC6 inhibition (∼88 Å^2^) and high predicted GI absorption, **7a** exhibited substantially higher lipophilicity (XLOGP3 = 4.85), lower aqueous solubility and markedly greater conformational flexibility (14 rotatable bonds). These features are consistent with reduced effective intracellular concentrations, increased nonspecific membrane partitioning, and a higher entropic penalty upon binding. PhaKinPro.^72^ also predicted lower hepatic stability and faster metabolic processing for **7a**, further limiting its biological performance (Table S4, Supporting Information).

In contrast, **7c** combines moderate lipophilicity (XLOGP3 = 3.76), improved solubility, and reduced conformational freedom (12 rotatable bonds), along with longer predicted plasma half-life and superior hepatic stability. These properties favor improved cellular accumulation and metabolic resilience.

Importantly, the MD simulations support these ADMET differences. Compound **7a** sampled more extensive conformational space, exhibiting larger fluctuations in the cap region and less persistent interaction with HDAC6, consistent with its higher rotatable-bond count. Conversely, **7c** maintained a more stable binding pose, lower ligand RMSD and a more coherent hydrogen-bonding pattern throughout the simulation. Consistent with these observations, the marked drop-off between enzymatic potency (IC_5__0_ = 7 nM for HDAC6) and cellular antiproliferative activity (∼3 μM) for **7c** can be attributed to permeability- and exposure-related factors combined with conformational stabilization within the HDAC6 pocket.

Together, these computational findings provide a coherent hypothesis for the enzymatic-to-cellular IC_50_ drop-off observed experimentally: although both compounds bind HDAC6 with high affinity, **7c** possesses a more balanced physicochemical and pharmacokinetic profile, enabling more efficient translation of target engagement into cellular antiproliferative activity.

## 3 Conclusions

In this work, a novel series of benzodioxol-benzyl-hydroxamates hybrids was successfully designed and synthesized, affording compounds **7a–f** with excellent purities (99–100%) and high overall yields (71–94%). To the best of our knowledge, all compounds reported here are unprecedented. Biological evaluation demonstrated consistent cytotoxicity against hematological cancer cell lines, with compounds **7a** and **7c** standing out as the most promising candidates. Compound **7a** combined strong activity in K-562 cells (IC_50_ = 3.0 μM) with a favorable selectivity profile, while **7c** exhibited remarkable HDAC6 inhibition in the nanomolar range (∼7 nM) and high selectivity over HDAC1 and HDAC8. These findings were supported by enzymatic assays, Western blot analysis confirming HDAC6 modulation, and molecular modeling studies, which provided structural insights consistent with experimental data. Combined ADMET predictions provided additional mechanistic support, helping to rationalize the different cellular responses observed for **7a** and **7c.**

Altogether, the integration of synthetic, biochemical, cellular, and computational approaches validates the hybridization strategy employed and highlights **7a** and **7c** as novel and selective HDAC6 inhibitors with anticancer potential. Beyond contributing new chemical entities, this work provides a mechanistic understanding and a clear framework for further optimization, reinforcing the therapeutic relevance of HDAC6 as a target in hematological malignancies.

## 4 Experimental Section

### 4.1 Synthesis Overview and Basic Materials

The experimental procedures involved standard laboratory glassware and equipment commonly employed in organic synthesis laboratories. Reagents and solvents were commercially sourced from Synth, Merck, Sigma-Aldrich-Merck, and Oakwood Chemicals. Reaction progress was monitored by thin-layer chromatography (TLC) using aluminum-backed silica gel 60 GF_254_ plates (Merck) with a thickness of 0.20 mm. Chromatograms were visualized under UV light at wavelengths of λ = 254 and 366 nm, followed by visualization using iodine and ninhydrin solutions. Extraction utilized saturated solutions of K_2_CO_3_ and NaCl. Purification was achieved through column chromatography using silica gel 60 (230–400 mesh) or automated liquid chromatography with an Isolera Prime automated column purifier (Biotage). Anhydrous solvents such as THF and DCM were treated and purified by distillation according to established literature protocols.^73^ Reactions sensitive to air and moisture were conducted under an inert atmosphere of N_2_.

### 4.2 Nuclear Magnetic Resonance Spectroscopy

The synthesis intermediates and final synthesis compounds were characterized by proton nuclear magnetic resonance (^1^H NMR) and carbon-13 nuclear magnetic resonance (^13^C NMR) spectroscopy using a Bruker Advanced-DPX-300 spectrometer at frequencies of 300 and 75 MHz, respectively. The compounds were dissolved in deuterated chloroform (CDCl_3_, Sigma-Aldrich) or deuterated dimethyl sulfoxide (DMSO-d_6_, Cambridge Isotope Laboratories). Chemical shifts (δ) were expressed in parts per million (ppm). Signal multiplicities were reported as singlet (s), broad singlet (bs), apparent singlet (as), doublet (d), triplet (t), apparent triplet (at), doublet of doublets (dd), quartet (q), and multiplet (m). Coupling constants (J) of the signals were expressed in Hertz (Hz).

### 4.3 Purity Content Analysis

The purity analyses of the synthesized molecules were conducted at the Analytical Center of the Department of Pharmaceutical Sciences at USP. High-performance liquid chromatography (HPLC) was employed using a liquid–solid column system, utilizing the Shimadzu Proeminence chromatograph equipped with a Promenex C-18 110 Å analytical column (5 μm, 150 × 4.6 mm). Samples were diluted in DMSO to a concentration of 0.25 mg/mL. Detection was performed using ultraviolet (UV) light at 254 nm, with an injection volume of 20 μL. The eluent system consisted of deionized water and acetonitrile (ACN), each containing 0.1% trifluoroacetic acid (TFA), with a flow rate set at 1 mL/min. The chromatographic run initiated with 5% ACN/TFA mixture, reaching 100% after 20 min.

### 4.4 Synthetic Methods

#### 4.4.1 Synthesis of Compound 5

Compound **5** was synthesized as shown in Scheme 2.

**Scheme 2:**
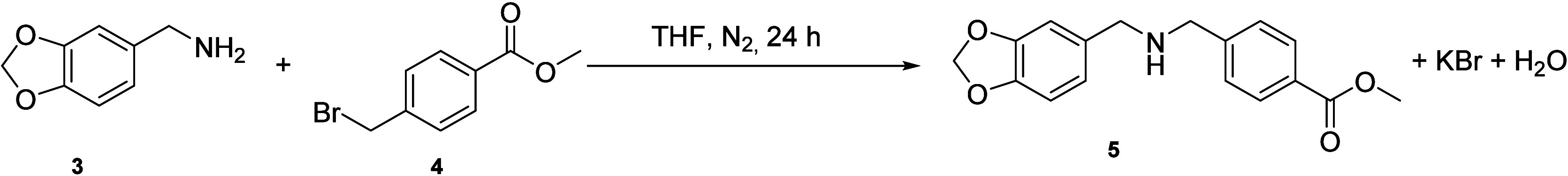
Synthesis of Intermediate **5**

In a 25 mL round-bottom flask equipped with a reflux condenser, under a nitrogen atmosphere and magnetic stirring, piperonylamine **3** (0.5 mmol -75.3 mg) in THF (5 mL) and 4-bromomethyl–methyl benzoate **4** (0.4 mmol -98 mg) were added. The reaction mixture was heated under reflux in a nitrogen atmosphere for 24 h. After this period, the reaction solvent (THF) was removed using a rotary evaporator, and to the resulting solid, 15 mL of saturated aqueous K_2_CO_3_ solution was added, and the mixture was stirred for 15 min. The product was extracted with ethyl acetate (3 × 15 mL), the organic phase was combined and dried over anhydrous Na_2_SO_4_. The solvent was removed under reduced pressure, and the residue was purified by column chromatography (CC) using a hexane: ethyl acetate gradient from 3:7 to 1:9 as eluent.

##### 4.4.1.1 4-Methyl-(((benzo­[1,3]­dioxol-5-ylmethyl)­amino)­methyl)­benzoate) 5

The product was obtained with a 87% yield as a yellow solid. ^1^H NMR (300 MHz, CDCl_3_, δ = ppm): 8.01 (d, J = 8.1 Hz, 2H, CH-Ar-4); 7.42 (d, J = 8.1 Hz, 2H, CH-Ar-3), 6.86 (1H, s, CH-Ar-12); 6.76 (2H, s, CH-Ar-9, 10); 5.94 (2H, s, OCH2O-13); 3.91 (3H, s, CH3O-14); 3.84 (2H, s, CH2–7); 3.71 (2H, s, CH2–6). ^13^C NMR (75 MHz, CDCl_3_, δ = ppm): 167.1; 147.9; 146.8; 145.7; 134.0; 129.8; 129.0; 128.1; 121.4; 108.8; 108.2; 101.0; 53.0; 52.6; 52.1.

#### 4.4.2 Synthesis of Acylated Intermediates

Compounds **6a–f** were synthesized as shown in Scheme 3.

**Scheme 3:**
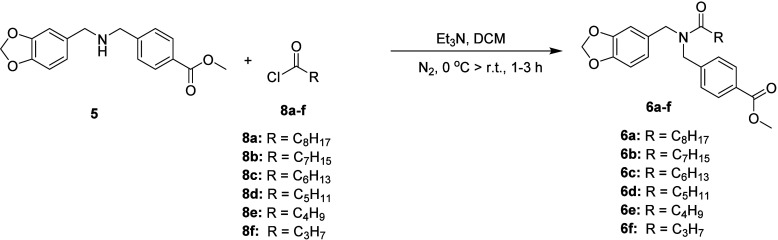
Synthesis of Compounds **6a–f**

In a 25 mL round-bottom flask containing intermediate **5** (1.2 mmol – 359.18 mg), under a nitrogen atmosphere, 10 mL of anhydrous dichloromethane was added. The mixture was stirred until intermediate **5** dissolved completely. The solution was then cooled in an ice bath at 0 °C, followed by the addition of 2 equiv of anhydrous triethylamine (2.0 mmol – 0.28 mL). The mixture was stirred for 5 min, and then the respective acyl chloride (1 mmol) was added dropwise. The reaction mixture was stirred continuously under a nitrogen atmosphere at room temperature for 1 to 3 h. After complete consumption of intermediate **5**, an aqueous solution of K_2_CO_3_ was added to the reaction mixture, and extraction was performed with dichloromethane (3 × 20 mL). The organic phases were combined and dried with anhydrous Na_2_SO_4_. The solvent was removed under reduced pressure to yield a viscous oil, which was purified by column chromatography (CC) using isocratic elution with hexane:ethyl acetate (7:3).

##### 4.4.2.1 Methyl 4-((N-(benzo­[d]­[1,3]­dioxol-5-ylmethyl)­nonanamide)­methyl)­benzoate 6a

The product was obtained from nonanoyl chloride (**8a**) (1.0 mmol) as a yellow oil in 93% yield. ^1^H NMR (300 MHz, CDCl_3_, δ = ppm, signal duplication): 8.04, 8.01, 7.98, 7.95 (2H, m, CH-Ar-4); 7.27, 7.23, 7.21, 7.19 (2H, m, CH-Ar-3), 6.79, 6.76, 6.74, 6.70, 6.63, 6.60, 6.56 (3H, m, CH-Ar-09, 10, 12); 5.96, 5.93 (2H, s, OCH2O-13); 4.61, 4.50 (2H, s, CH2–6); 4.48, 4.34 (2H, s, CH2–7); 3.92, 3.91 (3H, s, CH3–23); 2.46, 2.44, 2.41, 2.37, 2.35, 2.33 (2H, m, C15); 1.73, 1.71, 1.68, 1.66 (2H, m, C16); 1.36, 1.30, 1.26 (8H, m, C17,C18, C19, C20, C21) 0.89, 0.87, 0.84 (3H, m, C22). ^13^C NMR (75 MHz, CDCl_3_, δ = ppm): 173.4, 166.5, 166.5, 148.1, 147.7, 142.6, 130.8, 129.9, 129.8, 129.6, 129.4, 127.7, 126.0, 121.4, 119.4, 108.5, 108.2, 107.8, 106.6, 100.9, 100.7, 53.1, 51.8, 51.7, 49.8, 49.4, 47.8, 47.4, 33.0, 31.5, 28.8, 25.1, 25.0, 22.3, 13.7.

##### 4.4.2.2 Methyl 4-((N-(benzo­[d]­[1,3]­dioxol-5-ylmethyl-octanamide)­methyl)­benzoate – Intermediate 6b

The product was obtained from octanoyl chloride (**8b**) (1.0 mmol) as a yellow oil in 97% yield. ^1^H NMR (300 MHz, CDCl_3_, δ = ppm, signal duplication): 8.03, 8.01, 7.98, 7.95 (2H, m, CH-Ar-4); 7.26, 7.24, 7.21, 7.19 (2H, m, CH-Ar-3), 6.78, 6.76, 6.74, 6.70 (3H, m, CH-Ar-09, 10, 12); 5.96, 5.93 (2H, s, OCH2O-13); 4.61, 4.50 (2H, s, CH2–6); 4.48, 4.34 (2H, s, CH2–7); 3.91, 3.90 (3H, s, CH3–22); 2.45, 2.43, 2.40, 2.37, 2.34, 2.33 (2H, m, C15); 1.73, 1.681 (2H, m, C16); 1.36, 1.331, 1.30, 1.27, 1.25, 1.22 (8H, m, C17,C18, C19,C20) 0.89, 0.87, 0.85 (3H, m, C21). ^13^C NMR (75 MHz, CDCl_3_, δ = ppm): 174.2, 167.4, 167.2, 148.9, 148.5, 147.8, 147.6, 143.4, 142.6, 131.7, 130.8, 130.6, 130.4, 130.2, 129.8, 128.6, 126.8, 122.3, 120.3, 109.4, 109.1, 108.7, 107.5, 101.8, 101.6, 52.7, 52.6, 50.6, 50.2, 48.7, 48.3, 33.9, 33.8, 32.2, 29.9, 29.6, 26.03, 25.9, 23.1, 14.6.

##### 4.4.2.3 Methyl 4-((N-(benzo­[d]­[1,3]­dioxol-5-ylmethyl-heptanamide)­methyl)­benzoate 6c

The product was obtained from heptanoyl chloride (**8c**) (1.0 mmol) as a yellow oil in 80% yield. ^1^H NMR (300 MHz, CDCl_3_, δ = ppm, signal duplication): 8.04, 8.01, 7.98, 7.96 (2H, m, CH-Ar-4); 7.27, 7.24, 7.22, 7.19 (2H, m, CH-Ar-3), 6.79, 6.76, 6.73, 6.70 (3H, m, CH-Ar-09, 10, 12); 5.9, 5.93 (2H, s, OCH2O-13); 4.61, 4.50 (2H, s, CH2–6); 4.48, 4.35 (2H, s, CH2–7); 3.93, 3.91 (3H, s, CH3–21); 2.47, 2.44, 2.42, 2.38, 2.36, 2.33 (2H, m, C15); 1.71, 1.68, 1.66 (2H, m, C16); 1.37, 1.29, 1.27, 1.25 (6H, m, C17,C18, C19) 0.90, 0.87 (3H, m, C20). ^13^C NMR (75 MHz, CDCl_3_, δ = ppm): 174.2, 167.4, 167.2, 148.9, 148.5, 147.8, 147.6, 143.4, 142.6, 130.7, 131.7, 130.8, 130.4, 130.2, 129.8, 128.6, 126.8, 122.3, 120.3, 109.4, 108.7, 107.5, 101.8, 101.5, 52.7, 52.6, 50.6, 50.2, 48.7, 48.3, 33.8, 32.1, 25.9, 25.9, 23.0, 14.5.

##### 4.4.2.4 Methyl 4-((N-(benzo­[d]­[1,3]­dioxol-5-ylmethyl-hexanamide)­methyl)­benzoate 6d

The product was obtained from hexanoyl chloride (**8d**) (1.0 mmol) as a yellow oil in 97% yield. ^1^H NMR (300 MHz, CDCl_3_, δ = ppm, signal duplication): 8.03, 8.01, 7.98, 7.95 (2H, m, CH-Ar-4); 7.26, 7.23, 7.21, 7.18 (2H, m, CH-Ar-3), 6.78, 6.76, 6.73, 6.70, 6.69, 6.60 (3H, m, CH-Ar-09, 10, 12); 5.96, 5.93 (2H, s, OCH2O-13); 4.60, 4.50 (2H, s, CH2–6); 4.48, 4.34 (2H, s, CH2–7); 3.92, 3.90 (3H, s, CH3–20); 2.46, 2.43, 2.41, 2.37, 2.34, 2.32 (2H, m, C15); 1.70 (2H, m, C16); 1.31 (4H, m, C17,C18) 0.89, 0.87 (3H, m, C19). ^13^C NMR (75 MHz, CDCl_3_, δ = ppm): 174.2, 167.4, 167.2, 148.9, 148.5, 147.6, 143.4, 142.6, 130.8, 130.6, 130.4, 128.6, 126.8, 122.3, 120.3, 109.3, 109.1, 108.7, 107.4, 101.8, 52.7, 52.6, 50.6, 50.2, 48.7, 48.3, 33.8, 32.1, 25.7, 25.6, 23.0, 14.4.

##### 4.4.2.5 4-((N-(benzo­[d]­[1,3]­dioxol-5-ylmethyl)­pentanamide)­methyl)­benzoate 6e

The product was obtained from pentanoyl chloride (**8e**) (1.0 mmol) as a yellow oil in 72% yield. ^1^H NMR (300 MHz, CDCl_3_, δ = ppm, signal duplication): 8.03, 8.00, 7.97, 7.95 (2H, m, CH-Ar-4); 7.26, 7.23, 7.21, 7.18 (2H, m, CH-Ar-3), 6.78, 6.75, 6.73, 6.69, 6.62, 6.59, 6.56 (3H, m, CH-Ar-09, 10, 12); 5.95, 5.92 (2H, s, OCH2O-13); 4.51, 4.50 (2H, s, CH2–6); 4.49, 4.35 (2H, s, CH2–7); 3.91, 3.92 (3H, s, CH3–19); 2.47, 2.44, 2.42 (2H, t, J = 9 Hz, C15); 1.72, 1.69, 1.67, 1.64 (2H, m, C16); 1.40, 1.38, 1.36, 1.33, 1.32, 1.27, 1.25, 1.24, 1.22 (2H, m, C17); 0.94, 0.91, 0.89, 0.86 (3H, m, C18). ^13^C NMR (75 MHz, CDCl_3_, δ = ppm): 173.4, 166.56, 166.3, 148.1, 147.7, 146.9, 142.6, 141.7, 130.84, 129.9, 129.7, 129.6, 129.3, 128.9, 127.7, 126.0, 121.4, 119.4, 108.5, 107.8, 106.6, 100.96, 100.7, 51.8, 51.7, 49.8, 49.38, 47.8, 47.4, 32.7, 27.2, 27.1, 22.2, 13.5.

##### 4.4.2.6 4-((N-(benzo­[d]­[1,3]­dioxol-5-ylmethyl)­butanamide)­methyl)­benzoate 6f

The product was obtained from butanoyl chloride (**8f**) (1.0 mmol) as a yellow oil in 62% yield. ^1^H NMR (300 MHz, CDCl_3_, δ = ppm, signal duplication): 8.03, 8.00, 7.98, 7.95 (2H, m, CH-Ar-4); 7.26, 7.23, 7.21, 7.19 (2H, m, CH-Ar-3), 6.78, 6.76, 6.73, 6.70 (3H, m, CH-Ar-09, 10, 12); 5.96, 5.92 (2H, s, OCH2O-13); 4.60, 4.50 (2H, s, CH2–6); 4.48, 4.34 (2H, s, CH2–7); 3.91, 3.90 (3H, s, CH3–18); 2.45, 2.42, 2.40, 2.36, 2.34, 2.31 (2H, m, C15); 1.78, 1.76, 1.73, 1.70, 1.68 (2H, m, C16); 1.00, 0.97, 0.93, 0.91 (3H, m, C17). ^13^C NMR (75 MHz, CDCl_3_, δ = ppm): 173.8, 167.0, 166.7, 148.5, 148.1, 147.4, 147.2, 143.0, 142.2, 131.2, 130.3, 130.0, 129.8, 129.4, 128.2, 126.4, 121.8, 119.8, 108.9, 108.7, 108.2, 107.0, 101.4, 101.1, 52.2, 50.2, 49.8, 48.3, 47.9, 33.4, 31.7, 29.2, 22.6, 14.1.

#### 4.4.3 Preparation of Hydroxamic Acid Derivatives

Hydroxamic acid derivatives **7a–f** were prepared as shown in Scheme 4.

**Scheme 4:**
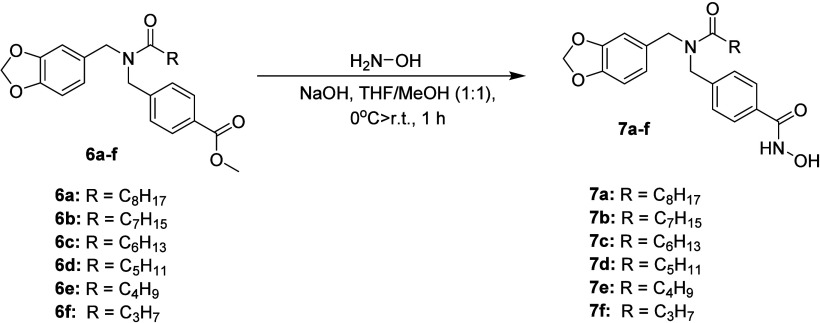
Synthesis of Hydroxamic Acid Derivatives **7a–f**

In a 10 mL round-bottom flask under magnetic stirring, 0.1599 g of sodium hydroxide (NaOH, 4 mmol, 8 equiv) was dissolved in 1.65 mL of aqueous hydroxylamine solution (50% w/v, 25 mmol, 50 equiv) at 0 °C. The solution was kept stirring at 0 °C for 5 min, after which a solution containing the respective intermediate **6a**–**f** (0.5 mmol, 1 equiv) in tetrahydrofuran (THF) and methanol (1:1, 1 mL) was added dropwise. The mixture was stirred continuously at room temperature for 1 h. After this period, 15 mL of distilled water was added, and the resulting solution was extracted 3 times with 15 mL of ethyl acetate. The organic phases were combined, washed with saturated aqueous NaCl solution, and dried with Na_2_SO_4_. After filtration, the solvent was removed under reduced pressure to yield the desired product.

##### 4.4.3.1 4-((N-(benzo­[d]­[1,3]­dioxol-5-ylmethyl)­octanamido)­methyl)-*N*-hydroxybenzamide 7a

The product was obtained from intermediate (**6a**) (0.5 mmol) as a light-brown solid in a yield of 75%. ^1^H NMR (300 MHz, DMSO-d6, δ = ppm, signal duplication): 11.16 (1H, bs, OH); 9.00 (1H, bs, NH); 7.75, 7.73, 7.71, 7.70, 7.67, 7.66 (2H, m, CH-Ar-4); 7.26, 7.25, 7.23, 7.21, 7.19 (2H, m, CH-Ar-3), 6.89, 6.87, 6.86, 6.85, 6.84, 6.82, 6.81, 6.80, 6.77, 6.70, 6.66, 6.63 (3H, m, CH-Ar-9, 10, 12); 6.00, 5.99, 5.97, 5.96 (2H, s, OCH2O-13); 4.54, 4.50 (2H, s, CH2–6); 4.43, 4.40 (2H, s, CH2–7); 2.41, 2.38, 2.36, 2.32, 2.30, 2.29, 2.27, 2.26 (2H, m, C15); 1.51 (2H, m, C16); 1.21, 1.20, 1.19 (10H, m, C17, C18, C19, C20, C21); 0.86, 0.85, 0.83, 0.82, 0.81 (3H, m, C22). ^13^C NMR (75 MHz, DMSO-d6, δ = ppm): 172.7, 163.8, 147.6, 147.3, 146.4, 146.3, 141.2, 140.8, 131.6, 131.4, 131.1, 127.3, 127.2, 126.9, 126.2, 121.1, 119.6, 108.3, 108.2, 108.3, 108.2, 108.0, 107.0, 100.9, 100.8, 50.0, 49.5, 48.0, 47.9, 32.1, 32.0, 31.1, 28.7, 28.6, 28.53, 24.76, 22.0, 13.8. Purity: 95.3% (254 nm).

##### 4.4.3.2 4-((N-(benzo­[d]­[1,3]­dioxol-5-ylmethyl)­octanamido)­methyl)-*N*-hydroxybenzamide 7b

The product was obtained from intermediate (**6b**) (0.5 mmol) as a beige solid in a yield of 98%. ^1^H NMR (300 MHz, DMSO-d6, δ = ppm, signal duplication): 11.15 (1H, bs, OH); 8.98 (1H, bs, NH); 7.75, 7.73, 7.71, 7.70, 7.67, 7.66 (2H, m, CH-Ar-4); 7.25, 7.23, 7.21, 7.19 (2H, m, CH-Ar-3), 6.89, 6.87, 6.86, 6.85, 6.84, 6.82, 6.81, 6.80, 6.77, 6.76, 6.73, 6.70, 6.67, 6.65, 6.62 (3H, m, CH-Ar-9, 10, 12); 5.97, 5.96 (2H, s, OCH2O-13); 4.54, 4.51­(2H, s, CH2–6); 4.43, 4.41 (2H, s, CH2–7); 2.41, 2.38, 2.36, 2.32, 2.30 2.27 (2H, m, C15); 1.52, 1.50, 1.48 (2H, m, C16); 1.21, 1.19 (8H, m, C17, C18, C19, C20); 0.86, 0.84, 0.83, 0.82, 0.80 (3H, m, C21). ^13^C NMR (75 MHz, DMSO-d6, δ = ppm): 172.7, 163.9, 147.6, 147.3, 146.4, 146.3 141.2, 140.8, 131.6, 131.4, 131.1, 127.3, 127.2, 126.9, 126.2, 121.1, 119.6, 108.3, 108.2, 108.3, 108.2, 108.0, 107.0, 100.9, 100.8, 50.0, 49.5, 48.0, 47.9, 32.0, 31.1, 28.5, 28.4, 24.7, 21.9, 13.8. Purity: 95.4% (254 nm).

##### 4.4.3.3 4-((N-(benzo­[d]­[1,3]­dioxol-5-ylmethyl)­heptanamido)­methyl)-*N*-hydroxybenzamide 7c

The product was obtained from intermediate (**6c**) (0.5 mmol) as a light-brown solid in a yield of 98%. ^1^H NMR (300 MHz, DMSO-d6, δ = ppm, signal duplication): 11.16 (1H, bs, OH); 8.99 (1H, bs, NH); 7.75, 7.73, 7.70, 7.67 (2H, m, CH-Ar-4); 7.25, 7.23, 7.21 (2H, m, CH-Ar-3), 6.89, 6.86, 6.84, 6.81, 6.77, 6.73, 6.70, 6.67, 6.65, 6.63 (3H, m, CH-Ar-9, 10, 12); 5.99, 5.97 (2H, s, OCH2O-13); 4.54, 4.51­(2H, s, CH2–6); 4.43, 4.42 (2H, s, CH2–7); 2.41, 2.38, 2.36, 2.32, 2.30, 2.28 (2H, m, C15); 1.55, 1.52, 1.50 (2H, m, C16); 1.25, 1.23, 1.21, 1.19 (6H, m, C17, C18, C19); 0.86, 0.84, 0.81, 0.79 (3H, m, C20). ^13^C NMR (75 MHz, DMSO-d6, δ = ppm): 172.8, 163.8, 147.6, 147.3, 146.4, 146.3, 141.2, 140.8, 131.6, 131.4, 131.1, 127.3, 127.2, 126.9, 126.3, 121.1, 119.7, 108.3, 108.2, 108.0, 107.1, 101.0, 100.8, 50.0, 49.6, 48.0, 47.9, 32.1, 32.1, 31.0, 24.7, 21.9, 13.7. Purity: 100% (254 nm).

##### 4.4.3.4 4-((N-(benzo­[d]­[1,3]­dioxol-5-ylmethyl)­hexanamido)­methyl)-*N*-hydroxybenzamide 7d

The product was obtained from intermediate (**6d**) (0.5 mmol) as a light-brown solid in a yield of 97%. ^1^H NMR (300 MHz, DMSO-d6, δ = ppm, signal duplication): 11.15 (1H, bs, OH); 8.98 (1H, bs, NH); 7.75, 7.72, 7.70, 7.67 (2H, m, CH-Ar-5); 7.25, 7.23, 7.21 (2H, m, CH-Ar-4), 6.89, 6.86, 6.84, 6.81, 6.77, 6.73, 6.70, 6.67, 6.65, 6.62 (3H, m, CH-Ar-9, 10, 12); 5.99, 5.97 (2H, s, OCH2O-14); 4.54, 4.51­(2H, s, CH2–6); 4.43, 4.41 (2H, s, CH2–7); 2.38, 2.36, 2.30, 2.27 (2H, m, C15); 1.25, 1.23, 1.22, 1.20 (6H, m, C16, C17, C18); 0.85, 0.83, 0.81, 0.79 (3H, m, C19). ^13^C NMR (75 MHz, DMSO-d6, δ = ppm): 172.7, 164.0, 147.6, 147.3, 146.4, 146.2, 141.2, 140.8, 131.6, 131.4, 131.1, 127.3, 127.2, 126.9, 126.2, 121.1, 119.6, 108.3–108.2, 108.0, 107.0, 100.9, 100.8, 50.0, 49.5, 48.0, 47.8, 32.1, 30.8, 24.4, 13.7. Purity: 95.9% (254 nm).

##### 4.4.3.5 4-((N-(benzo­[d]­[1,3]­dioxol-5-ylmethyl)­butanamido)­methyl)-*N*-hydroxybenzamide 7e

The product was obtained from intermediate (**6e**) (0.5 mmol) as a light-brown solid in a yield of 83%. ^1^H NMR (300 MHz, DMSO-d6, δ = ppm, signal duplication): 11.15 (1H, bs, OH); 8.98 (1H, bs, NH); 7.76, 7.74, 7.71, 7.69 (2H, m, CH-Ar-4); 7.36, 7.26, 7.24, 7.22 (2H, m, CH-Ar-3), 6.90, 6.87, 6.85, 6.82, 6.78, 6.74, 6.70, 6.68, 6.66, 6.63 (3H, m, CH-Ar-9, 10, 12); 6.00,5.98 (2H, s, OCH2O-13); 4.54,4.51 (2H, s, CH2–6); 4.44, 4.43 (2H, s, CH2–7); 2.41, 2.38, 2.36, 2.32, 2.30, 2.27 (2H, m, C15); 1.57, 1.55, 1.53, 1.50, 1.45 (2H, m, C16); 1.31, 1.29, 1.26, 1.23, 1.21 (2H, m, C17); 0.87, 0.84, 0.81, 0.79 (3H, m, C18). ^13^C NMR (75 MHz, DMSO-d6, δ = ppm): 172.5; 147.6; 147.3; 146.4; 146.2; 141.2; 140.8; 131.6; 131.4; 131.1; 127.7; 126.9; 126.2; 121.0; 119.6; 108.3; 108.2; 108.0; 107.1; 100.9; 100.8; 59.7; 49.9; 49.5; 47.9; 47.7; 20.7; 18.2; 18.1; 14.0; 13.6. Purity: 98.4% (254 nm).

##### 4.4.3.6 4-((N-(benzo­[d]­[1,3]­dioxol-5-ylmethyl)­pentanamido)­methyl)-*N*-hydroxybenzamide 7f

The product was obtained from intermediate (**6f**) (0.5 mmol) as a beige solid in a yield of 64%. ^1^H NMR (300 MHz, DMSO-d6, δ = ppm, signal duplication): 10.36 (1H, bs, OH); 8.18 (1H, bs, NH); 7.75; 7.72; 7.70; 7.67 (2H, m, CH-Ar-4); 7.25; 7.23; 7.21 (2H, m, CH-Ar-3), 6.90; 6.87; 6.84; 6.82; 6.78; 6.74; 6.70; 6.66; 6.63­(3H, m, CH-Ar-9, 10, 12); 6.00; 5.98 (2H, s, OCH2O-13); 4.54; 4.50 (2H, s, CH2–6); 4.43; 4.41 (2H, s, CH2–7); 2.41–2.28 (2H, m, C15); 1.58–1.51 (2H, m, C16); 0.89–0.82 (3H, m, C17). ^13^C NMR (75 MHz, DMSO-d6, δ = ppm): 172.6; 147.6; 147.3; 146.3; 146.2; 141.2; 140.8; 131.6; 131.4; 131.1; 130.8; 127.3; 127.3; 126.9; 126.2; 121.0; 119.6; 108.2; 108.1; 108.0; 107.0; 100.9; 100.8; 49.9; 49.5; 47.9; 47.8; 26.9; 26.9; 21.7; 13.7. Purity: 100% (254 nm).

### 4.5 Biological Assays

All Biological assays were conducted in collaboration with Professor João Agostinho Machado-Neto, Professor Letícia Veras Costa-Lotufo, and the PhD’s student Jorge Antonio Elias Godoy Carlos, affiliated with the Institute of Biomedical Sciences at the University of São Paulo (ICB-USP). Jurkat, Namalwa, K-562, HS-5, CCD-18Co, and HaCaT cells were cultured according to the American Type Culture Collection (ATCC) or Deutsche Sammlung von Mikroorganismen and Zellkulturen (DMSZ) recommendations.

### 4.6 Cell Viability Assays (Cytotoxicity)

The MTT (3-[4,5-dimethylthiazol-2-yl]-2,5-diphenyl-tetrazolium bromide) assay was performed to assess cell viability and proliferation. Briefly, cells in the logarithmic growth phase were seeded in 96-well plates at a density of 2 × 10^4^ cells/mL for suspension cells or 5 × 10^3^ cells/mL for adherent cells (preseeded for 18 h). Cells were then treated with capsaicin, nexturastat A, tubastatin A, or compound **7a**–**f** at concentrations ranging from 0.0032 to 50 μM for 72 h. After treatment, 10 μL of MTT solution (5 mg/mL) was added to each well and incubated for an additional 4 h under the same conditions. The reaction was stopped by adding 100 μL of 0.1 N HCl in isopropanol, and absorbance was measured at 540 nm using a microplate reader (Thermoplate TP Reader, Tokai Hit, Japan). The percentage of growth inhibition was calculated as [1 – (OD_treated/OD_control)] × 100.

### 4.7 Cell Death Mechanism

Cells were treated with the compounds of interest or vehicle for 48 h, collected, and resuspended in binding buffer containing 1 μg/mL of 7AAD or propidium iodide (PI) and 1 μg/mL of APC annexin V (BD Bioscience). After incubation shielded from light for 15 min at room temperature, the samples were then analyzed on a FACSCalibur (BD Bioscience), with subsequent data collection and analysis performed using FlowJo software (Treestar, Inc., San Carlos, CA, USA) and statistical analysis conducted using GraphPad Prism software. Ten thousand events were acquired for each sample.

### 4.8 Cell Cycle Analysis

A total of 6 × 10^5^ cells per well were seeded in 6-well plates in the presence of the compounds of interest or vehicle for 48 h. The cells were then fixed in 70% ethanol for at least 2 h at 4 °C before analysis and incubated with 20 μg/mL propidium iodide (PI) containing 10 μg/mL RNase A for 30 min at room temperature. Cellular fluorescence analyses will be conducted using a FACSCalibur, and cell cycle phase distributions determined by FlowJo software (Treestar, Inc., San Carlos, CA, USA).

### 4.9 Western Blot

Cells treated with the compounds of interest or vehicle for 24 h were subjected to total protein extraction using a protein extraction buffer containing 100 mM Tris (pH 7.6), 1% Triton X-100, 150 mM NaCl, 2 mM PMSF, 10 μM Na_3_VO_4_, 100 mM NaF, 10 mM Na_4_P_2_O_7_, and 4 mM EDTA. After 30 min of incubation on ice, samples were centrifuged to remove cellular debris. The protein samples were then subjected to SDS-PAGE polyacrylamide gel electrophoresis using an electrophoresis apparatus, followed by protein transfer from the gel to a nitrocellulose membrane. The membrane was blocked with 5% milk and incubated with specific primary antibodies diluted in blocking buffer, followed by incubation with HRP-conjugated secondary antibodies. Antibodies against acetyl-histone H3 Lys9 (#9649), histone H3 (#4499), acetyl-α-tubulin Lys40 (#5335), and α-tubulin (#2144) were obtained from Cell Signaling Technology (Danvers, MA, USA). The detection system was performed according to the instructions of the SuperSignal West Dura Extended Duration Substrate System (Thermo Fisher Scientific, San Jose, CA, USA) using the G:BOX Chemi XX6 gel doc system (Syngene, Cambridge, UK).

### 4.10 Enzymatic Assays

*In vitro* enzymatic inhibition assays were performed using recombinant human HDAC1 and HDAC6 acquired from ENZO Life Sciences AG (Lausen, CH), while HDAC8 was produced as previously described.^74^ Evaluation of inhibitors in an enzymatic assay was conducted as described in previous publications.^74,75^ For HDAC1, a fluorogenic peptide derived from p53 (Ac-RHKK­(Acetyl)-AMC) was utilized. For HDAC6, the substrate (Abz-SRGGK­(thio-TFA)­FFRR-NH2) was used as previously described.^74^ Enzymatic inhibition of HDAC8 was determined with a homogeneous fluorescence assay using the fluorogenic substrate ZMAL (Z­(Ac)­Lys-AMC), as previously described.^75^ All measurements were conducted in an assay buffer (50 mM HEPES, 150 mM NaCl, 5 mM MgCl_2_, 1 mM TCEP, and 0.2 mg/mL BSA, pH 7.4 adjusted with NaOH) at 37 °C. An Envision 2104 Multilabel plate reader (PerkinElmer, Waltham, MA), with an excitation wavelength of 380 ± 8 nm and an emission wavelength of 430 ± 8 nm, was used to measure fluorescence intensity.

### 4.11 Molecular Modeling

Modeling methods are provided in detail in the Supporting Information. Docking was performed according to^70^ and poses were visually inspected prior sending to molecular dynamics (MD) simulations. Prior to MD simulations, all protein structures were prepared using the Protein Wizard Preparation tool, with standard options, and the homology model was further refined to remove sterical clashes. In parallel, we compared against the proposed AlphaFold3 model, however, the conserved ligand binding pocket was collapsed. MD simulations were carried out by using the Desmond engine^76^ with the OPLS4 force-field.^77^ We have used the standard Zn^2+^ treatment provided by the OPLS4 force field, with the hydroxamate bound in the monodentate conformation to the Zinc ion by a single restriction with the carbonyl group. This allows us to evaluate and compare the effects of the different cap substitutions in the different HDACs.

The system encompassed the protein–ligand/cofactor complex, a predefined water model (TIP3P) as a solvent and counterions (Na^+^ or Cl^–^ adjusted to neutralize the overall system charge).^78^ The system was treated in a cubic box (10 Å) with a periodic boundary condition specifying the size of the box from the box edges to any atom of the protein. Short-range Coulombic interactions were calculated using 1 fs time steps and 9.0 Å cutoff value, whereas long-range Coulombic interactions were estimated using the Smooth Particle Mesh Ewald method.^79^

Each HDAC+Ligand system was subjected to at least 2.5 μs simulations (split into five replicas of 500 ns, each) with random seeds. Those simulations are long enough to evaluate protein–ligand binding and 5 replicas are indeed sufficient by the ACS guidelines for MD simulation reports (which recommends at least 3 independent replicas,^80^ REF). Even though some of the protein RMSD values do change between replicas (Figure 6), overall, the ligand’s RMSD remains relatively stable (<3Å for most replicas). Representative frames of the simulations were retrieved using hierarchical clustering analyses (trj_cluster.py, implemented in Maestro 2024.3, Schrödinger LCC) according to the RMSD of ligand’s heavy atoms (1 Å as cutoff). All the trajectory and interaction data are available on the Zenodo repository (code: 10.5281/zenodo.16679743, made available upon publication). MD trajectories were visualized, and figures were generated using PyMOL v.3.1 (Schrödinger LCC, New York, NY, USA).

**Figure 6:**
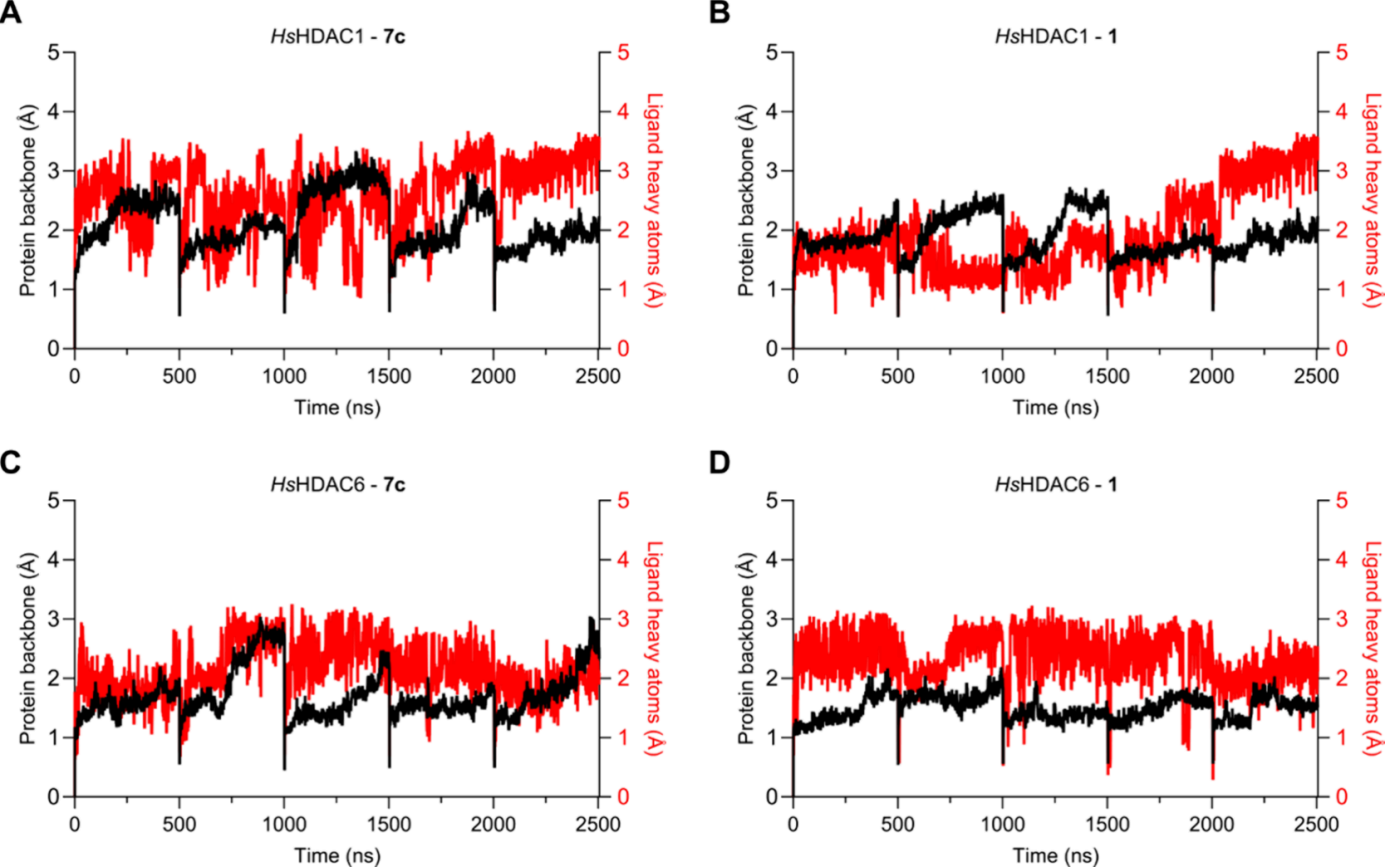
Root-mean square deviation (RMSD) for protein backbone (black) and ligand’s heavy atoms (red line) for HDAC1 (A,B) and HDAC6 (C,D) simulations (5x 500 ns).

### 4.12 *In silico* ADMET and Pharmacokinetic Predictions

#### 4.12.1 Pharmacokinetic Predictions (PhaKinPro)

The pharmacokinetic properties of the test compounds (Table S3, Supporting Information) and their associated fragment-contribution maps were assessed using Pharmacokinetics Profiler (PhaKinPro) web tool, available at https://phakinpro.mml.unc.edu/. The detailed description of the model’s development and validation are described in the literature.^72^

#### 4.12.2 In silico ADMET Evaluation (SwissADME)

Physicochemical and ADMET parameters were predicted using the SwissADME web platform (http://www.swissadme.ch). The canonical SMILES of compounds **7a**, **7c** and **1** were generated in ChemDraw 22.0 and submitted individually to the SwissADME interface using default parameters. The following descriptors were extracted: molecular weight, topological polar surface area (TPSA), hydrogen-bond acceptors/donors, number of rotatable bonds, lipophilicity (iLOGP, XLOGP3, WLOGP, MLOGP and consensus LogP), aqueous solubility (ESOL, Ali and SILICOS-IT models), gastrointestinal (GI) absorption, blood–brain barrier permeability, P-gp substrate prediction, cytochrome-P450 inhibition profile, bioavailability score and drug-likeness filters (Lipinski, Ghose, Veber, Egan and Muegge). All values are summarized in Table S4, Supporting Information).

## Supplementary Material



## Notes

^1^H and ^13^C NMR spectra for all compounds; HPLC purity profiles; additional experimental procedures and supplementary biological data (PDF)
