# Supplementary material for: Capsaicin-Inspired Hydroxamate Hybrids as Selective HDAC6 Inhibitors with Antiproliferative Activity in Hematological Malignancies
Source: ACS Omega. 2026 Jan 28;11(5):8415–30. doi: 10.1021/acsomega.5c11286 (PMC12903172; doi:10.1021/acsomega.5c11286)
Supplement: Supplementary file 1 [file ao5c11286_si_001.pdf]

Supporting Information

Capsaicin-Inspired Hydroxamate Hybrids as Selective HDAC6 Inhibitors with Antiproliferative Activity in Hematological Malignancies

Lara Gimenez Borges [a], Thais Nascimento de Oliveira Alves [a], Sandra Valeria Vassiliades [a], Jorge Antonio Elias Godoy Carlos [b], Karoline de Barros Waitman [a], Sebastian Hilscher [c], Mike Schutkowski [c], Wolfgang Sippl [c], Maurício Temotheo Tavares [d,e], Monica Franco Zannini Junqueira Toledo [a], Letícia Veras Costa-Lotufo [b], Thales Kronenberger [f,g,h], João Agostinho Machado-Neto [b], Roberto Parise-Filho [a]*

[a] Department of Pharmacy, Faculty of Pharmaceutical Sciences, University of São Paulo, São Paulo, 05508-900, Brazil.
[b] Department of Pharmacology, Institute of Biomedical Sciences, University of São Paulo, São Paulo, 05508-900, Brazil.
[c] Faculty of Biosciences, Martin-Luther-University of Halle-Wittenberg, 06120 Halle/Saale, Germany.
[d] Department of Cancer Biology, Dana-Farber Cancer Institute, Boston, MA 02215, United States.
[e] Department of Biological Chemistry and Molecular Pharmacology, Harvard Medical School, Boston, MA 02215, United States.
[f] Interfaculty Institute of Microbiology and Infection Medicine (IMIT), University of Tübingen, 72076 Tübingen, Germany.
[g] German Center for Infection Research (DZIF), 72076, Tübingen, Germany.
[h] School of Pharmacy, Faculty of Health Sciences, University of Eastern Finland, P.O. Box 1627, FI-70211 Kuopio, Finland.

*Email: roberto.parise@usp.br

Table of contents

Figure S1–S3. Apoptosis detection (Jurkat, Namalwa, K-562) — Annexin V/PI, IC_{50} (24 h). **S1–S3**

Molecular Modelling Protocols (structure optimization; docking setup; validation). **S4–S5**

Table S1. Docking scores for **7a–7f** (hHDAC6/DrHDAC6). **S5**

Figures S4–S5. Docking poses (**7a–7f**) in HDAC6 homologs. **S6–S7**

Molecular Dynamics Simulations (setup; analyses; MM/GBSA; visualization; data availability). **S8**

Figure S6. MD-derived binding modes (compounds **1** and **7c**) in HsHDAC1/HsHDAC6. **S9**

Table S2. Interaction frequencies and binding energies (5 × 500 ns). **S10**

Table S3. Key physicochemical descriptors and ADMET predictions (SwissADME) for compounds **1**, **7a** and **7c.** **S11**

Table S4. Preliminary pharmacokinetic properties predicted for **1**, **7a**, and **7c**. **S11**

References (Supporting Information Methods for molecular modeling protocols). **S12**

NMR Spectra (intermediates **5**, **6a–6f**; compounds **7a–7f**). **S13–S27**

Purity Analysis (HPLC chromatograms **7a–7f** and blank). **S27–S31**

Supporting Information

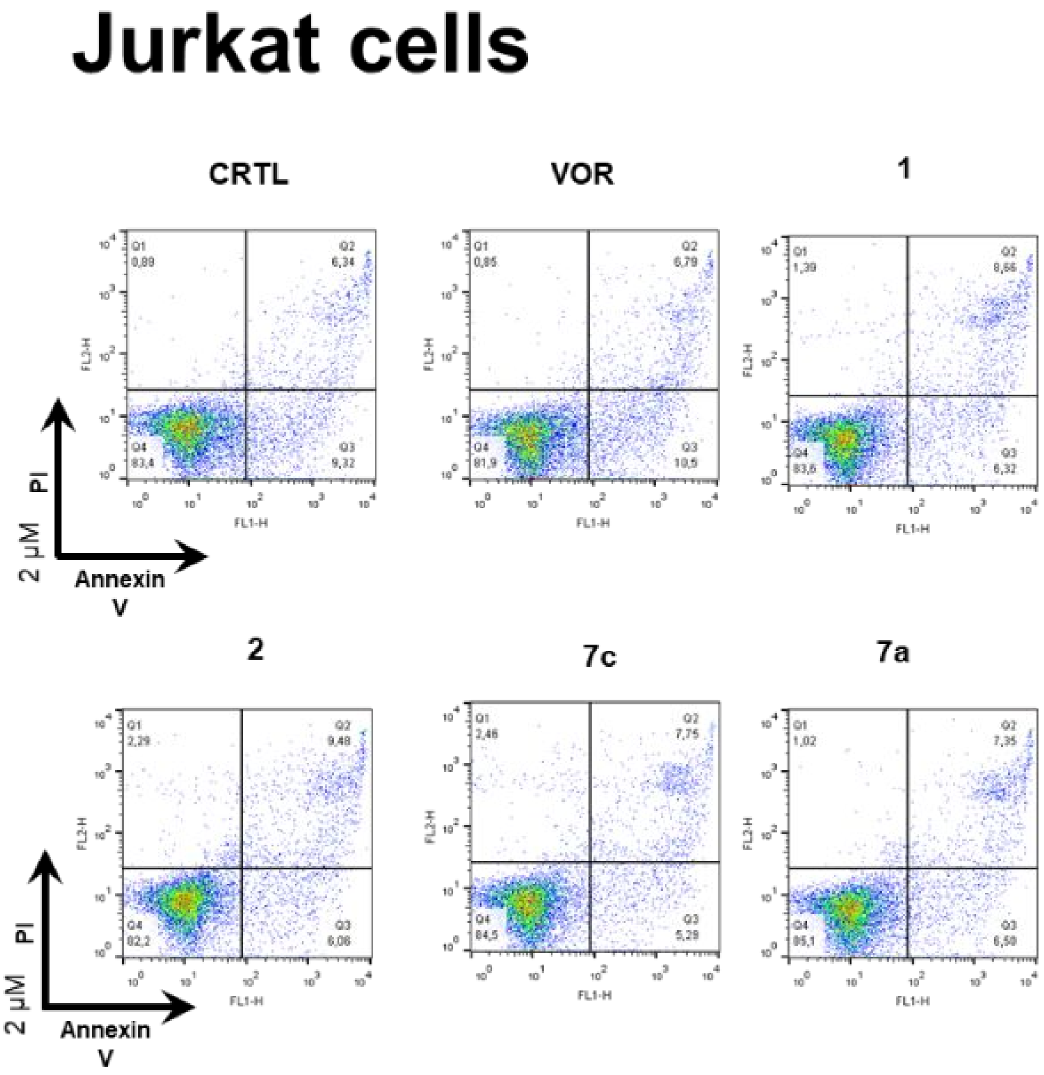

Figure S1. Apoptosis detection in Jurkat cells after treatment with vorinostat, **7a**, or **7c** at their respective IC_{50} concentrations for 24 h, followed by Annexin V/propidium iodide (PI) staining and flow cytometry analysis. Representative plots show Annexin V–positive apoptotic cells in quadrants Q2 and Q3.

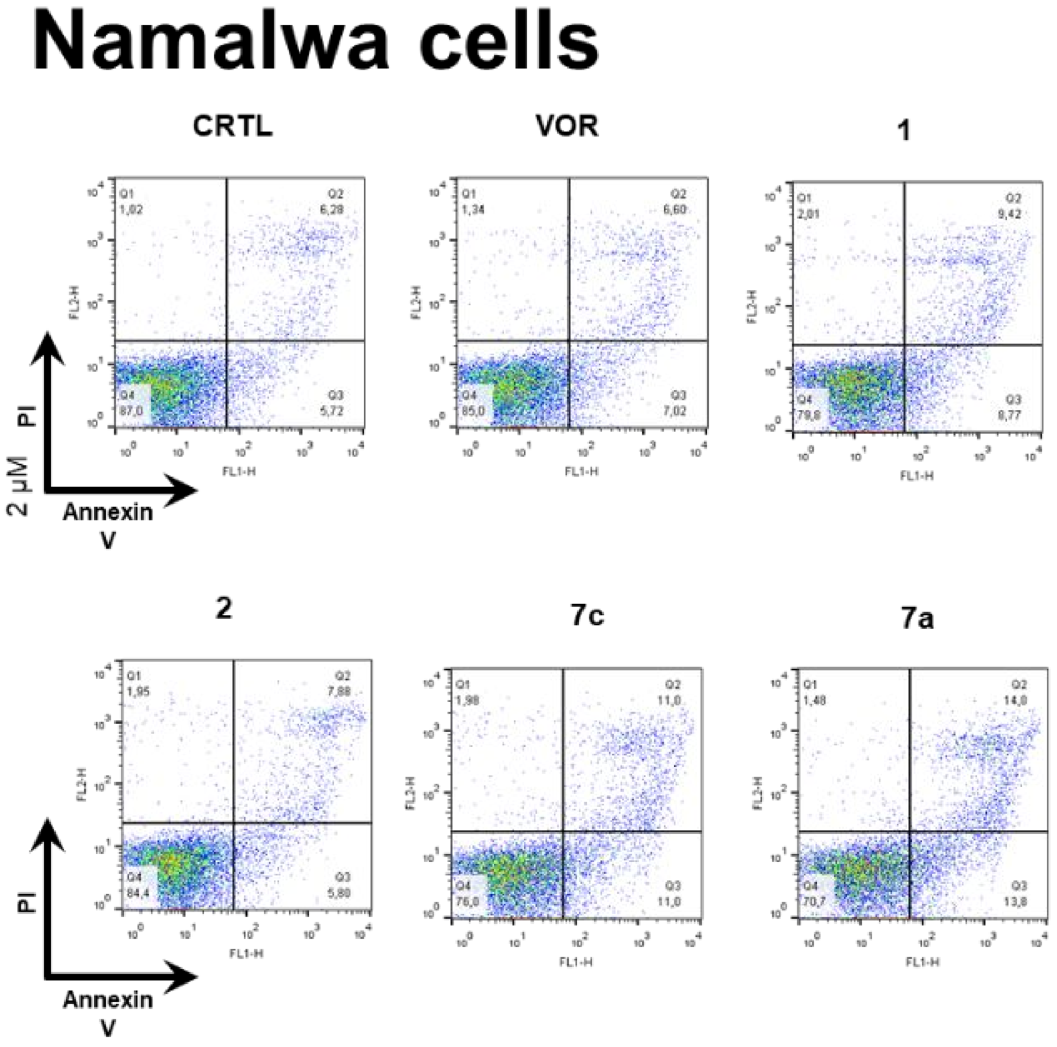

Figure S2. Apoptosis detection in Namalwa cells after treatment with vorinostat, **7a**, or **7c** at their respective IC_{50} concentrations for 24 h, followed by Annexin V/PI staining and flow cytometry analysis. Representative plots show Annexin V–positive apoptotic cells in quadrants Q2 and Q3.

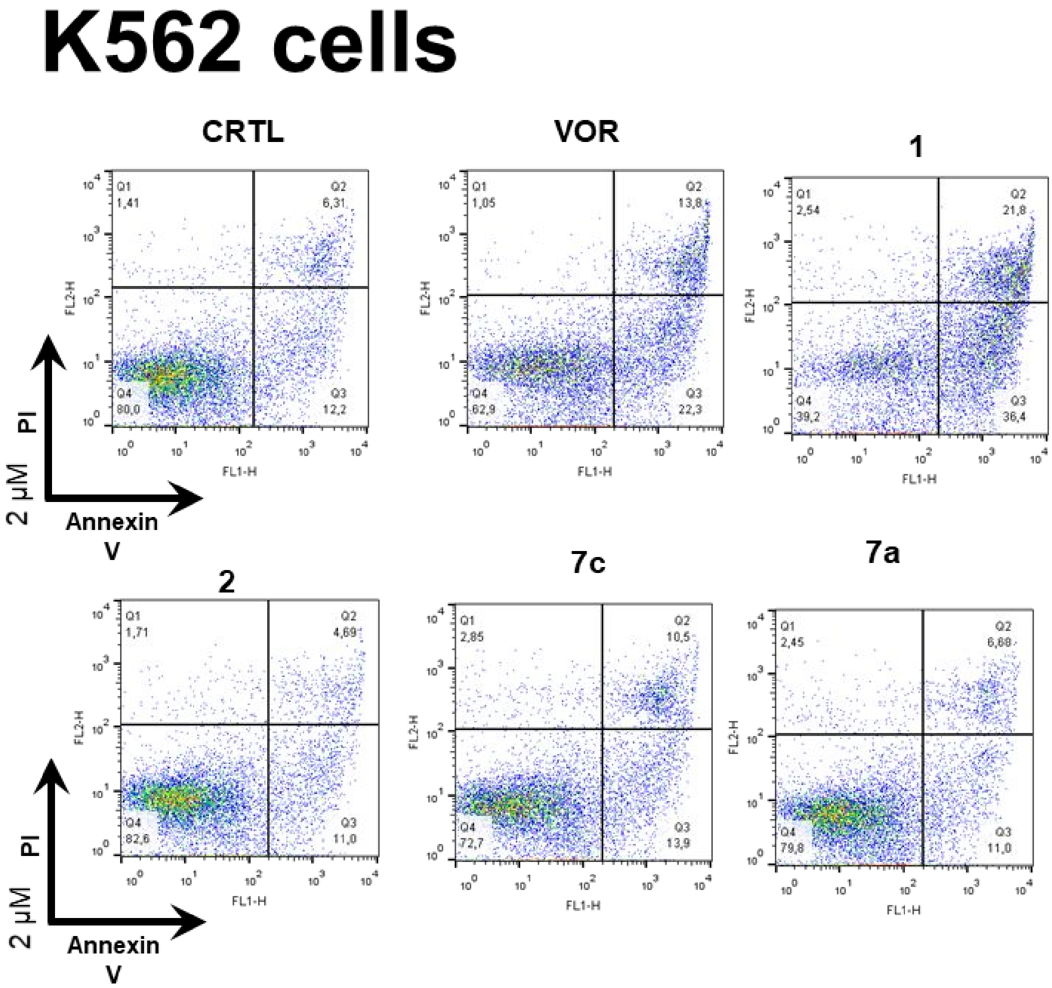

Figure S3. Apoptosis detection in K-562 cells after treatment with vorinostat, **7a**, or **7c** at their respective IC_{50} concentrations for 24 h, followed by Annexin V/PI staining and flow cytometry analysis. Representative plots show Annexin V–positive apoptotic cells in quadrants Q2 and Q3.

Supporting Information

Molecular modelling protocols

1.1. Molecular Structure Optimization

The molecular structures of the compounds were built using ChemBioDraw Ultra 20.1.1 (PerkinElmer) and subsequently optimized using Spartan'18 (WaveFunction, INC). The optimization was performed by energy minimization through molecular mechanics using the Merck Molecular Force Field (MMFF), the semi-empirical PM6 method, and the Hartree-Fock 3-21G method in vacuum.

1.2. Docking Simulations

The docking simulation protocols for the HDAC6 enzyme were selected based on redocking simulations performed in sextuplicate. The co-crystals of zebrafish and human HDAC6 enzymes were available in the Protein Data Bank (PDB). Redocking simulations were conducted using HDAC6 (PDB code: 5G0I, resolution 1.99 Å) and HDAC6 (PDB code: 5EDU, resolution 1.80 Å), where the co-crystallized ligands Nexturastat A and Trichostatin A were used, respectively (HAI; CHRISTIANSON, 2016; MIYAKE et al., 2016). For the redocking procedure, the binding site of the co-crystallized ligands was used as the docking origin, with a 10 Å radius. The crystallographic water molecules were removed. The four scoring functions available in GOLD 5.4 (CCDC) were tested: ASP, ChemPLP, GoldScore, and ChemScore. The method demonstrating the lowest root mean square deviation (RMSD) value was selected. The chosen scoring function for HDAC6 (5G0I) was ChemPLP, while for HDAC6 (5EDU), ASP was selected. Docking simulations were performed in sextuplicate, and the poses with the highest scores were selected for final visualization. The molecular docking images were generated using BIOVIA Discovery Studio 2021 (Dassault Systemes).

Molecular docking results and validation

Molecular docking is a computer-assisted drug design (CADD) strategy widely used in compound optimization, hit identification, and studies on interactions between ligands and targets. It is a method that predicts or defines the interactions between the ligand and the macromolecular target [1]. To analyze the potential interactions between the hybrids planned in this research and HDAC6, and to select those that present the best poses to proceed with the synthetic stage, a molecular docking study was conducted, using as targets the HDAC6 enzymes from *Danio rerio* (DrHDAC6; PDB code: 5G0I), complexed with nexturastat A [2], and human HDAC6 (hHDAC6; PDB code: 5EDU), crystallized with trichostatin A [3]. **Table S1** presents the score values in the docking simulations for the 11 planned compounds against the hHDAC6 and DrHDAC6 targets. From the obtained results, it can be observed that the compounds highlighted in green, **7a-c**, exhibited the highest average score results compared to the other compounds in the series, which were synthesized and experimentally evaluated against HDACs, indicating a superior fit within the enzyme's active site and, consequently, greater affinity for the target enzyme. According to the results, it can be inferred that compounds **7a-c** have a good fit with the DrHDAC6, and the hHDAC6. However, compound **7f** showed the lowest score value in both enzymes, suggesting that it does not fit properly into both isoforms. The results indicate that the longer the alkyl chain of the compound, the higher its docking score and the stronger its interaction with the enzyme's catalytic pocket. The

findings from molecular docking corroborate the experimental data, as compounds **7a-c** demonstrated superior HDAC6 inhibition and higher selectivity when tested against other isoforms. In contrast, compound **7f** exhibited the lowest inhibition values in both theoretical and experimental analyses.

The inhibition of the HDAC6 enzyme occurs through the binding between the inhibitor and the catalytic site, involving the chelation of the zinc ion (Zn^{2+}), often mediated by the ZBG present in the compounds (hydroxamic acid) and the enzyme's metal itself. Depending on the number of bonds between the ZBG and the Zn^{2+}, these interactions can be mono-, bi-, or tridentate [4–6]. However, other standard regions of HDAC inhibitors, such as the linker and cap, are also crucial for the binding between the compound and the enzyme. The linker is responsible for interactions in the narrow tunnel leading to the bottom of the cavity, while the cap explores interactions on the surface of the catalytic cavity [7]. Upon comparing the results of molecular docking studies, it was found that compound **7a** exhibited the most promising results in terms of score and interaction poses with DrHDAC6. This observation is significant, especially in contrast to the enzymatic inhibition activity of HDAC6, where compound **7a** did not stand out as much as **7c**. Analysis of the structures of compounds **7a** and **7c,** about the results obtained, reveals that while HDAC6 inhibitors need to have a larger "cap" portion, it is not feasible for them to possess a very long acyclic chain. On the contrary, it seems more advantageous to maintain a medium-sized and acyclic chain between five and seven carbon atoms. Additionally, although it was not the objective of this study, it is believed that the use of polar groups in the acyclic chain may favor additional interactions of the "cap" in the surface region of the HDAC6 enzyme [8].

Table S1. Mean of docking score values from molecular docking performed on HDAC6 homologs**.**

**Compounds**	**Ranking values in docking simulations**						
	***Dockings* in DrHDAC6 (PDB: 5G0I)**						
	**1**	**2**	**3**	**4**	**5**	**6**	**Mean**
**7a**	100.98	106.74	103.29	94.28	94.85	100.63	100.13
**7b**	100.12	102.59	101.48	101.72	102.64	100.68	101.54
**7c**	96.79	102.32	106.16	100.57	98.13	105.56	101.59
**7d**	102.89	99.65	96.27	98.36	98.8	101.66	99.61
**7e**	101.26	100.41	102.45	98.46	87.03	103.9	98.92
**7f**	84.08	83.85	88.09	86.79	84.04	84.22	85.18
**Compounds**	***Dockings* in hHDAC6 (PDB: 5EDU)**						
	**1**	**2**	**3**	**4**	**5**	**6**	**Mean**
**7a**	58.59	59.56	57.97	61.25	61.33	61.48	60.03
**7b**	60.90	57.29	61.78	60.82	60.00	57.76	59.76
**7c**	57.42	59.43	59.48	57.00	58.17	59.32	58.47
**7d**	56.94	57.53	57.61	56.29	58.36	56.35	57.18
**7e**	56.21	57.78	57.37	55.45	56.50	57.69	56.83
**7f**	56.17	54.64	55.54	55.07	55.47	56.78	55.61

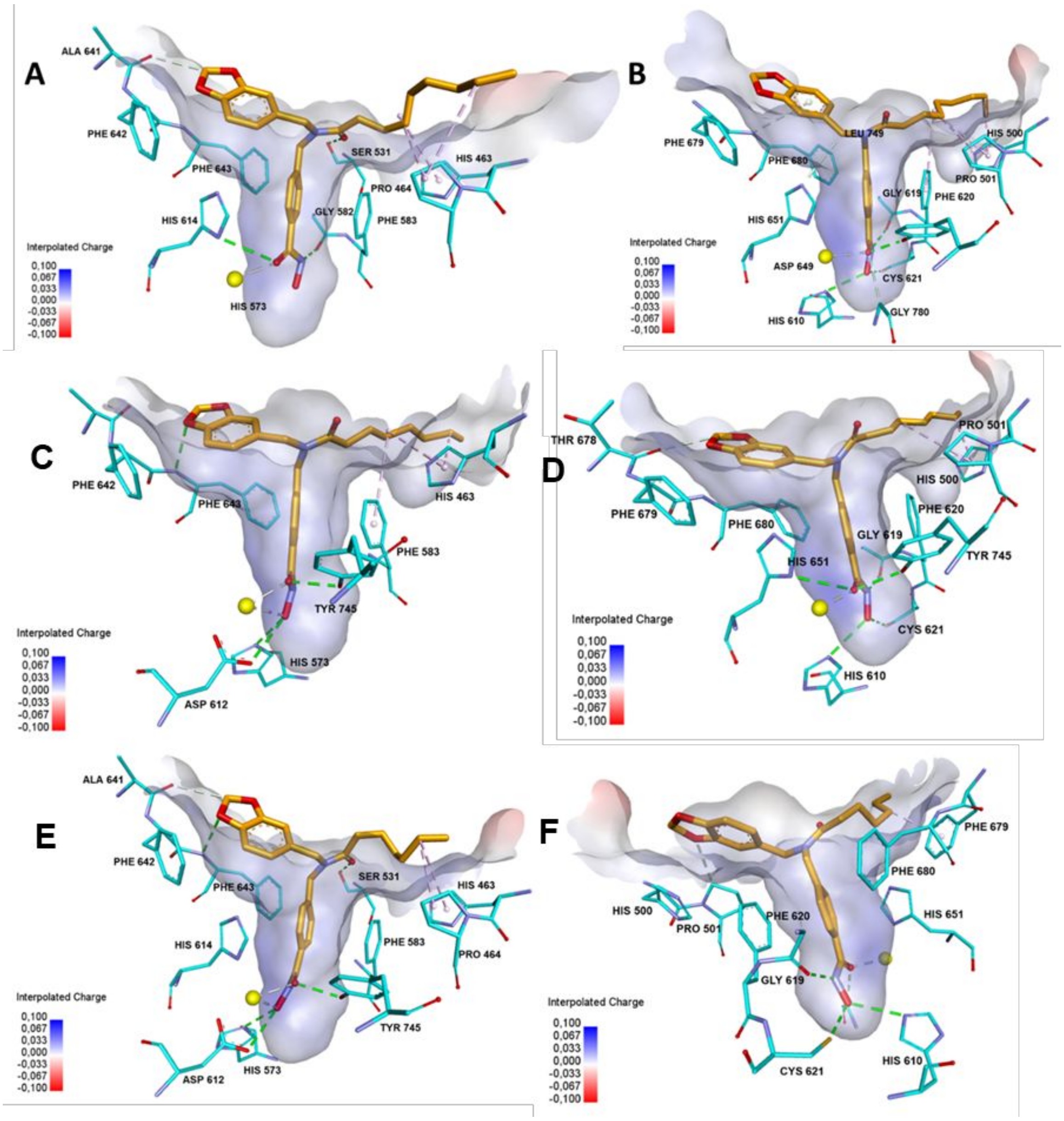

Figure S4. Best docking poses obtained for compound **7a** in interaction with DrHDAC6 (PDB code: 5G0I) (**A**) and hHDAC6 (PDB code: 5EDU) (**B**). Best docking poses obtained for compound **7b** in interaction with DrHDAC6 (PDB code: 5G0I) (**C**) and hHDAC6 (PDB code: 5EDU) (**D**).Best docking poses obtained for compound **7c** in interaction with DrHDAC6 (PDB code: 5G0I) (**E**) and hHDAC6 (PDB code: 5EDU) (**F**). Carbon atoms are represented in orange, oxygen in red, and nitrogen in blue. The enzyme surface corresponds to the interpolated charge of the catalytic site amino acids.

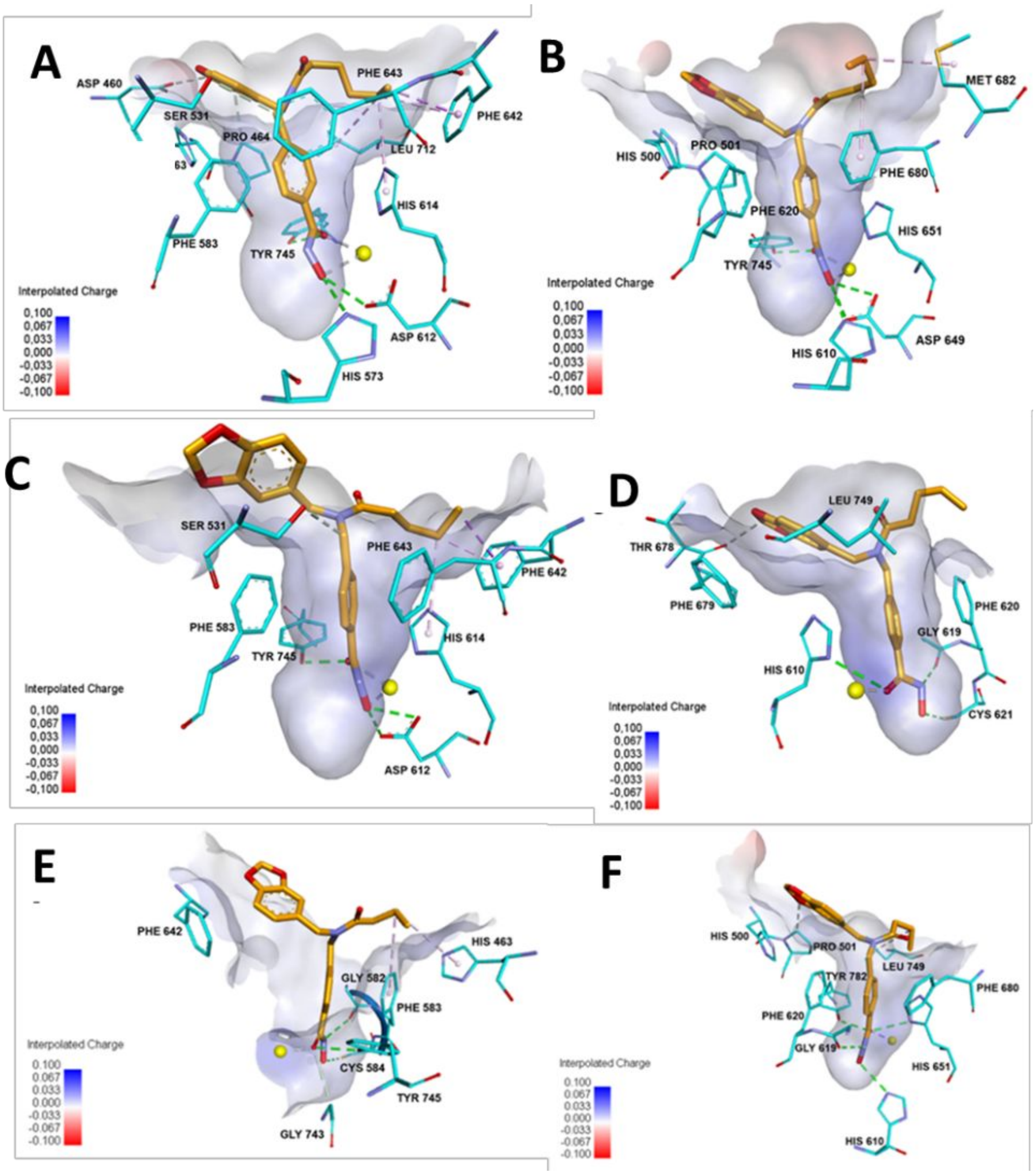

Figure S5. Best docking poses obtained for compound **7d** in interaction with DrHDAC6 (PDB code: 5G0I) (**A**) and hHDAC6 (PDB code: 5EDU) (**B**). Best docking poses obtained for compound **7e** in interaction with DrHDAC6 (PDB code: 5G0I) (**C**) and hHDAC6 (PDB code: 5EDU) (**D**). Best docking poses obtained for compound **7f** in interaction with DrHDAC6 (PDB code: 5G0I) (**E**) and hHDAC6 (PDB code: 5EDU) (**F**). Carbon atoms are represented in orange, oxygen in red, and nitrogen in blue. The enzyme surface corresponds to the interpolated charge of the catalytic site amino acids.

1.3. Molecular dynamics simulations.

Prior to MD simulations, all protein structures were prepared using the Protein Wizard Preparation tool, with standard options, and the homology model was further refined to remove sterical clashes. In parallel, we compared against the proposed AlphaFold3 model, however, the conserved ligand binding pocket was collapsed. MD simulations were carried out by using the Desmond engine [9] with the OPLS4 force-field [10]. The system encompassed the protein-ligand/cofactor complex, a predefined water model (TIP3P) as a solvent and counterions (Na^+ or Cl^- adjusted to neutralize the overall system charge) [11]. The system was treated in a cubic box (10 Å) with a periodic boundary condition specifying the size of the box from the box edges to any atom of the protein. Short-range coulombic interactions were calculated using 1 fs time steps and 9.0 Å cut-off value, whereas long-range coulombic interactions were estimated using the Smooth Particle Mesh Ewald method [12]. Each HDAC+Ligand system was subjected to at least 2.5 µs simulations (split into five replicas of 500 ns, each) with random seeds. Representative frames of the simulations were retrieved using hierarchical clustering analyses (trj_cluster.py, implemented in Maestro 2024.3, Schrödinger LCC) according to the RMSD of ligand's heavy atoms (1 Å as cut-off). All the trajectory and interaction data are available on the Zenodo repository (code: 10.5281/zenodo.16679743, made available upon publication). MD trajectories were visualized, and figures were generated using PyMOL v.3.1 (Schrödinger LCC, New York, NY, USA).

MD simulation trajectory analyses.

Protein-ligand interactions and atomic distances were calculated using the Simulation Interaction Diagram analysis pipeline (Maestro 2024.3, Schrödinger LCC). RMSD values of the protein backbone were used to monitor simulation equilibration and protein folding changes (all raw data is available in the repository).

MM/GBSA binding energy calculations. Molecular mechanics with generalized Born and surface area (MM/GBSA) predicts the binding free energy of protein-ligand complexes and the ranking of ligands based on the free energy could be correlated to the experimental binding affinities especially in a congeneric series. Every 20th frame from the simulations was considered for energy calculations with thermal_mmgbsa.py script. Calculated free-binding energies were normalized by the number of heavy atoms (HAC), according to the following formula: Ligand Efficiency = (Binding Energy)/(1 + ln(HAC)).

Visualization and plotting**.** Structural data visualization was conducted with PyMOL v.3.1 (Schrödinger LLC, New York, NY, USA). Data visualization was also completed by Python 3.7, seaborn (v0.12.2), matplotlib, and GraphPad Prism (v. 10.3 for Windows, GraphPad Software, San Diego, CA, USA).

Data and Software Availability Statement. All prepared structures, molecular dynamics (MD) trajectories, simulation configuration and parameter files, as well as raw and processed data related to HDAC–ligand interactions are available through the Zenodo repository under the DOI: 10.5281/zenodo.16679743 (accessible upon publication). Third-party software used in this study includes: GraphPad Prism version 10.2 (https://www.graphpad.com/), Schrödinger Suite 2024.3–2025.1 (https://www.schrodinger.com), and PyMOL version 2.5.2–3.1 (https://pymol.org/), each distributed under their respective licenses

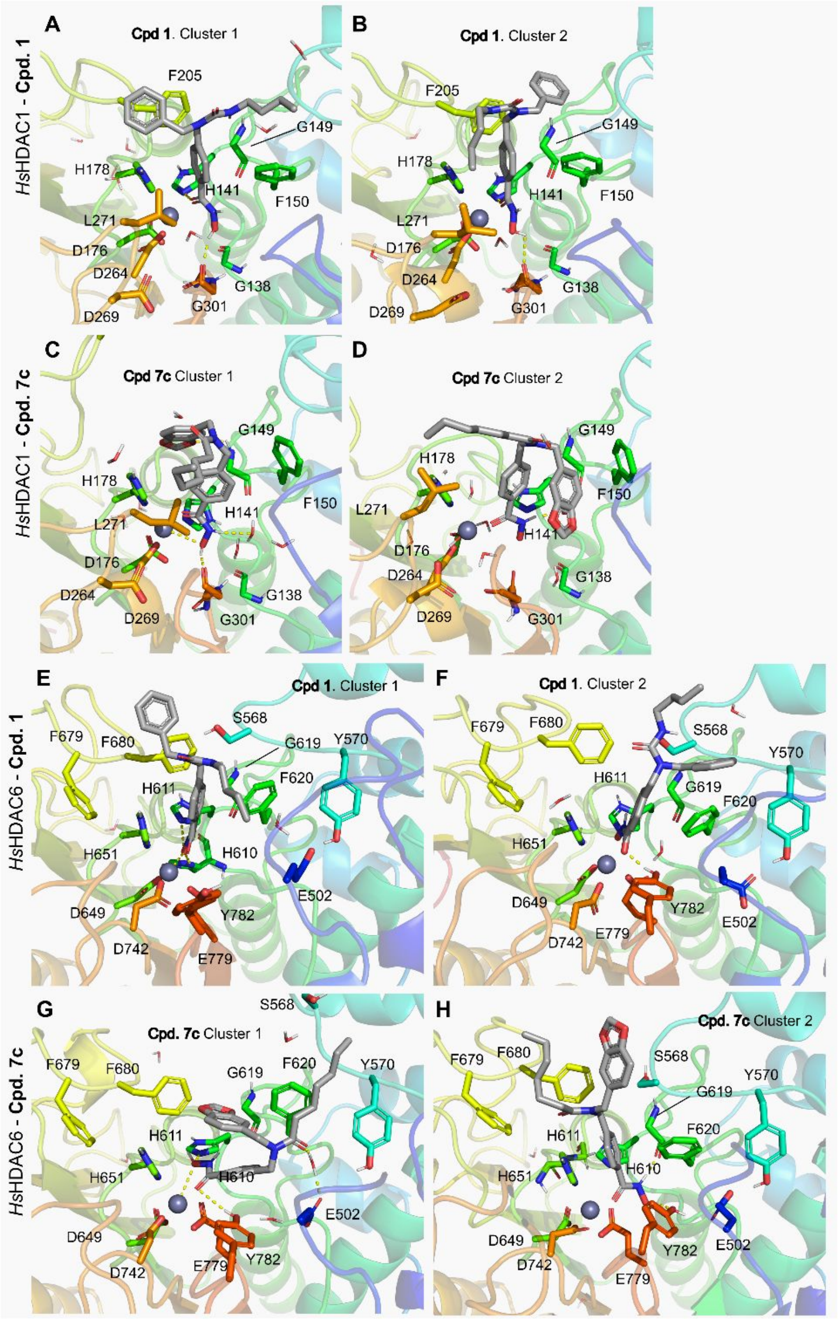

Figure S6. Potential binding mode for compounds 1 (A,B and E,F) and 7c (C,D and G,H) retrieved from relevant frames of the MD simulation for HsHDAC1 (A-D) and HsHDAC6 (E-H). Relevant frames were selected by hierarchical clustering (cutoff of 1 Å of ligand's heavy atoms RMSD) from the full trajectory of 5x500 ns. Most populated clusters (1st, left and 2nd, right) are displayed.

Table S2. Protein-ligand interaction frequency along the analysed simulation trajectories (5x500 ns). Energy terms are expressed in kcal/mol and ligand efficiency (LN) in kcal/mol.HAC

		**HsHDAC1**			**HsHDAC6**	
		**7c**	**1**		**7c**	**1**
**Stable reps**		**3/5**	**4/5**		**5/5**	**5/5**
**Zn**	E176	100	100	E649	100	100
	E264	100	100	E742	100	100
	H178	100	100	D779	100	100
	H141	45	35	H651	100	100
**ZBG**	G138		14	H611	44	
	G149	19	24	G619	38	59
	E269	13	19			
	G300	13				
	G301	12				
	Y303	14	26			
**Linker**	H141	19	13	F620	30	69
	F150	23	25	H651	35	52
	H178	20	24	F680	20	16
	F205		21	Y782	18	
**Cap**	F150		12	S568		11
	L271	10	10	Y570	10	
				D502	23	10
				F679		23
**Energy**	dG	-11.4	-13.9	dG	-24.2	-27.8
	Cou	-23.5	-23.3	Cou	-23.5	-22.8
	Hbond	-0.8	-0.8	Hbond	-0.9	-1.0
	Lipo	-15.0	-16.8	Lipo	-19.9	-19.6
	LN	-2.6	-3.3	LN	-5.5	-6.5

Table S3. Key physicochemical descriptors and ADMET predictions (SwissADME) for compounds **1**, **7a** and **7c**

Descriptor	7a	7c	Nexturastat A (1)
Molecular weight (g/mol)	440.53	412.48	341.40
TPSA (Å²)	88.10	88.10	81.67
H-bond acceptors	5	5	3
H-bond donors	2	2	3
Rotatable bonds	14	12	10
XlogP3	4.85	3.76	2.64
Consensus LogP	4.07	3.41	2.61
Solubility class (ESOL)	Moderately/poorly soluble	Moderately soluble	Soluble
Log S (ESOL)	–4.98	–4.27	–3.32
GI absorption	High	High	High
BBB permeation	No	No	No
P-gp substrate	No	No	No
CYP inhibition	2C9, 2D6	2C19, 2C9, 2D6	2C9
Bioavailability score	0.55	0.55	0.55
Lipinski violations	0	0	0
Veber violations	1 (rotors >10)	1 (rotors >10)	0

Table S4: Preliminary pharmacokinetic properties predicted for **1**, **7a**, and **7c**

Compound / Property	7a	7c	Nexturastat A (1)
**Hepatic Stability**	Between 30-60 min	60 min	60 min
Confidence	0,68	0,516	0,5533
**$t_{1/2}$ Sub-cellular**	> 30 min	> 30 min	> 30 min
Confidence	0,67	0,67	0,68
**$t_{1/2}$ Tissue**	<= 30 min	<= 30 min	<= 30 min
Confidence	0,59	0,63	0,68
**$t_{1/2}$ Plasma**	No prediction	>12 h	Between 1-6 h
Confidence	Out of AD	0,507	0,5
**Renal Clearance**	> 1.00 ml/min/kg	> 1.00 ml/min/kg	> 1.00 ml/min/kg
Confidence	0,56	0,564	0,576
**Microsomal Intrinsic Clearance**	>= 12 uL/min/mg	>= 12 uL/min/mg	< 12 uL/min/mg
Confidence	0,516	0,516	0,512
**Oral Bioavailability**	Above 0.8 F	Above 0.8 F	No prediction
Confidence	0,728	0,74	Out of AD
**BBB* Permeability**	Yes	Yes	Yes
Confidence	0,556	0,56	0,58
**CNS** Activity**	Yes	Yes	Yes
Confidence	0,884	0,884	0,908
**CACO2 Permeability**	Yes	Yes	Yes
Confidence	0,68	0,684	0,648
**Plasma Protein Binding**	Yes	Yes	Yes
Confidence	0.692	0.7	0.856

*BBB: blood brain barrier, **CNS: central nervous system

References for supporting information methods

(1) Sant'Anna, C. M. R. Molecular Modeling Methods in the Study and Design of Bioactive Compounds: An Introduction. *Revista Virtual de Química* **2009**, *1* (1). https://doi.org/10.5935/1984-6835.20090007.
(2) Miyake, Y.; Keusch, J. J.; Wang, L.; Saito, M.; Hess, D.; Wang, X.; Melancon, B. J.; Helquist, P.; Gut, H.; Matthias, P. Structural Insights into HDAC6 Tubulin Deacetylation and Its Selective Inhibition. *Nature Chemical Biology* **2016**, *12* (9), 748–754. https://doi.org/10.1038/nchembio.2140.
(3) Hai, Y.; Christianson, D. W. Histone Deacetylase 6 Structure and Molecular Basis of Catalysis and Inhibition. *Nature Chemical Biology* **2016**, *12* (9), 741–747. https://doi.org/10.1038/nchembio.2134.
(4) Raper, E. Complexes of Heterocyclic Thionates. Part 1. Complexes of Monodentate and Chelating Ligands. *Coordination Chemistry Reviews* **1996**, *153*, 199–255. https://doi.org/10.1016/0010-8545(95)01233-8.
(5) Wu, R.; Lu, Z.; Cao, Z.; Zhang, Y. Zinc Chelation with Hydroxamate in Histone Deacetylases Modulated by Water Access to the Linker Binding Channel. *Journal of the American Chemical Society* **2011**, *133* (16), 6110–6113. https://doi.org/10.1021/ja111104p.
(6) Zhang, L.; Zhang, J.; Jiang, Q.; Zhang, L.; Song, W. Zinc Binding Groups for Histone Deacetylase Inhibitors. *Journal of Enzyme Inhibition and Medicinal Chemistry* **2018**, *33* (1), 714–721. https://doi.org/10.1080/14756366.2017.1417274.
(7) Wang, X. X.; Wan, R. Z.; Liu, Z. P. Recent Advances in the Discovery of Potent and Selective HDAC6 Inhibitors. *European Journal of Medicinal Chemistry* **2018**, *143*, 1406–1418. https://doi.org/10.1016/j.ejmech.2017.10.040.
(8) Pina, A. S.; Roque, A. C. A. Studies on the Molecular Recognition between Bioactive Peptides and Angiotensin-Converting Enzyme. *Journal of Molecular Recognition* **2009**, *22* (2), 162–168. https://doi.org/10.1002/jmr.905.
(9) Bowers, K. J.; Chow, E.; Xu, H.; Dror, R. O.; Eastwood, M. P.; Gregersen, B. A.; Klepeis, J. L.; Kolossvary, I.; Moraes, M. A.; Sacerdoti, F. D.; Salmon, J. K.; Shan, Y.; Shaw, D. E. Scalable Algorithms for Molecular Dynamics Simulations on Commodity Clusters.
(10) Lu, C.; Wu, C.; Ghoreishi, D.; Chen, W.; Wang, L.; Damm, W.; Ross, G. A.; Dahlgren, M. K.; Russell, E.; Bargen, C. D. Von; Abel, R.; Friesner, R. A.; Harder, E. D. OPLS4 : Improving Force Field Accuracy on Challenging Regimes of Chemical Space. **2021**. https://doi.org/10.1021/acs.jctc.1c00302.
(11) Madura, J. D. Comparison of Simple Potential Functions for Simulating Liquid Water. **2014**, No. May. https://doi.org/10.1063/1.445869.
(12) Darden, T.; York, D.; Pedersen, L.; Darden, T.; York, D.; Pedersen, L. Particle Mesh Ewald : An Nlog (N) Method for Ewald Sums in Large Systems Particle Mesh Ewald : An N -Log (N) Method for Ewald Sums in Large Systems. **1993**, *10089*. https://doi.org/10.1063/1.464397.

Supporting Information

NMR Spectra

The synthetic intermediates and final compounds were characterized by proton and carbon-13 nuclear magnetic resonance (^1H and ^{13}C NMR) spectroscopy using a Bruker Advanced-DPX-300 spectrometer operating at 300 MHz and 75 MHz, respectively. The compounds were dissolved in deuterated chloroform ($CDCl_3$, Sigma-Aldrich) or deuterated dimethyl sulfoxide (DMSO-d6, Cambridge Isotope Laboratories). Chemical shifts (δ) were reported in parts per million (ppm). Signal multiplicities were assigned as singlet (s), broad singlet (bs), apparent singlet (as), doublet (d), triplet (t), apparent triplet (at), double doublet (dd), quartet (q), and multiplet (m). Coupling constants (J) were reported in Hertz (Hz).

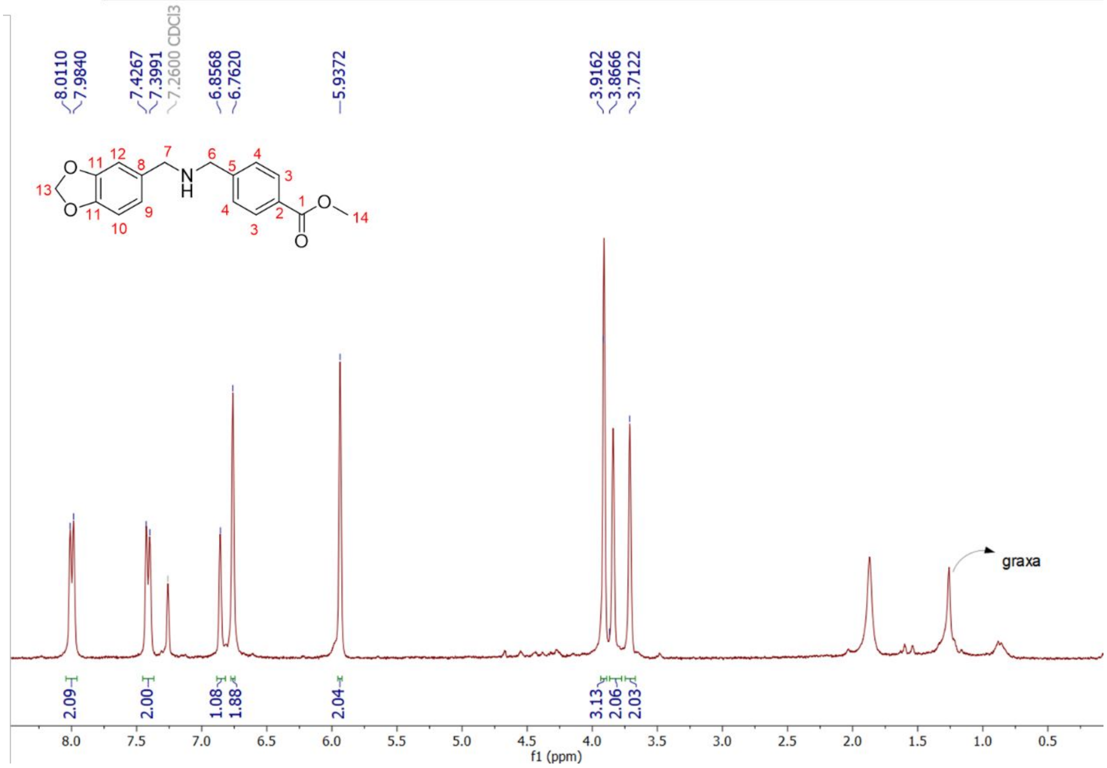

Figure S7.^1H NMR Spectrum of Intermediate **5** (300 MHz, $CDCl_3$, δ = ppm).

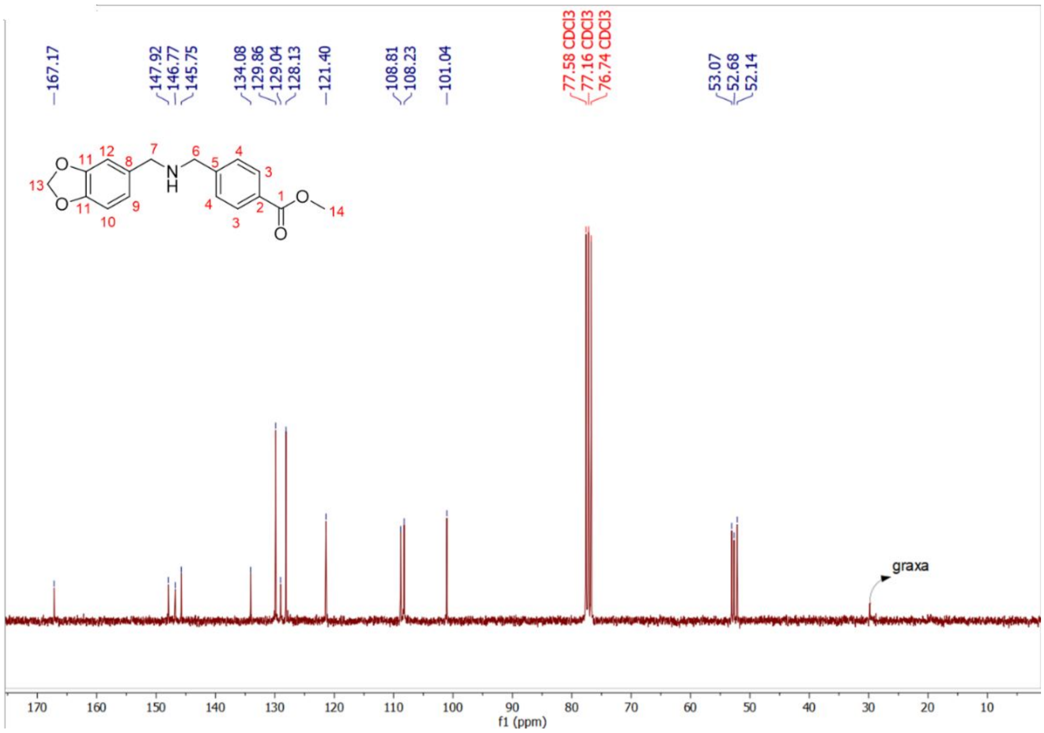

Figure S8.^{13}C NMR Spectrum of Intermediate **5** (75 MHz, $CDCl_3$, δ = ppm).

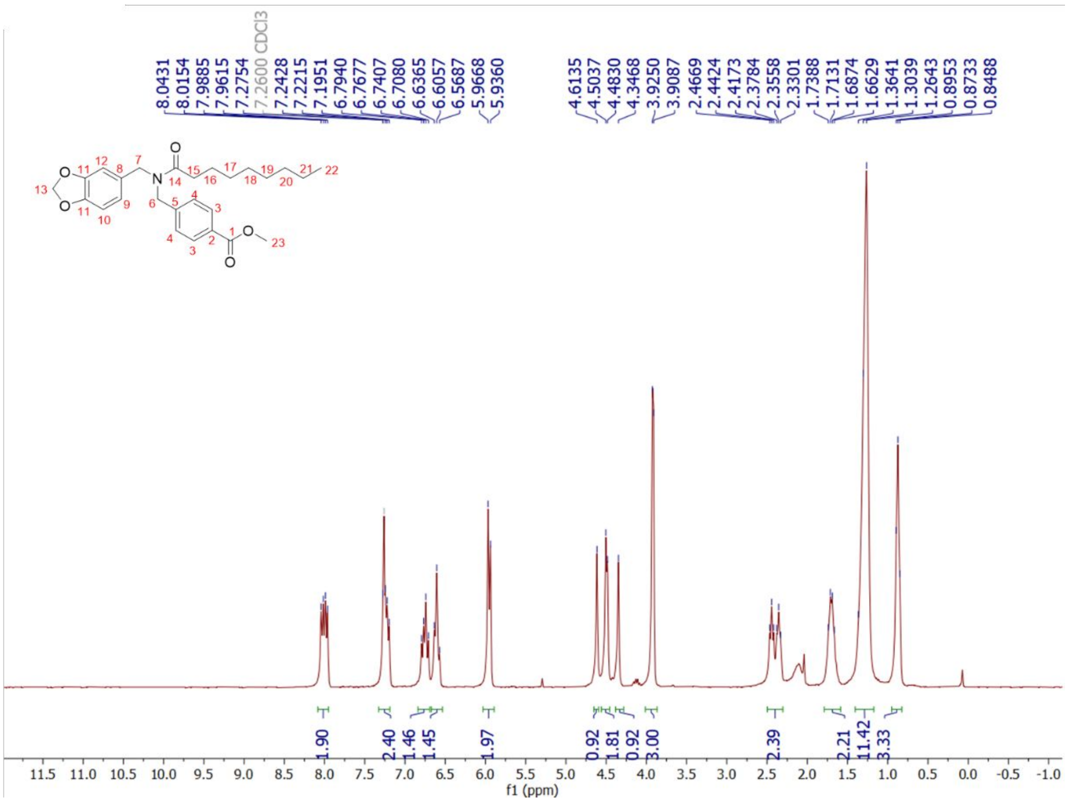

Figure S9. ^{1}H NMR Spectrum of Intermediate **6a** (300 MHz, $CDCl_3$, δ = ppm).

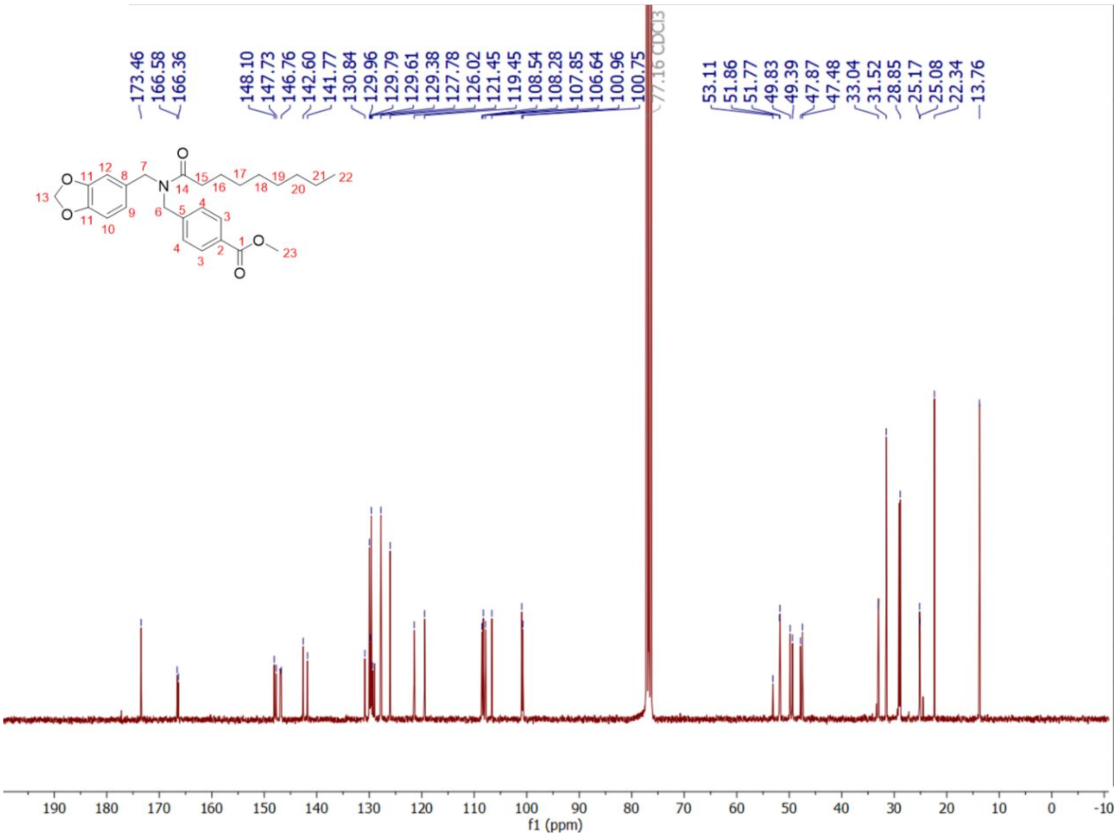

Figure S10. ^{13}C NMR Spectrum of Intermediate **6a** (75 MHz, $CDCl_3$, δ = ppm).

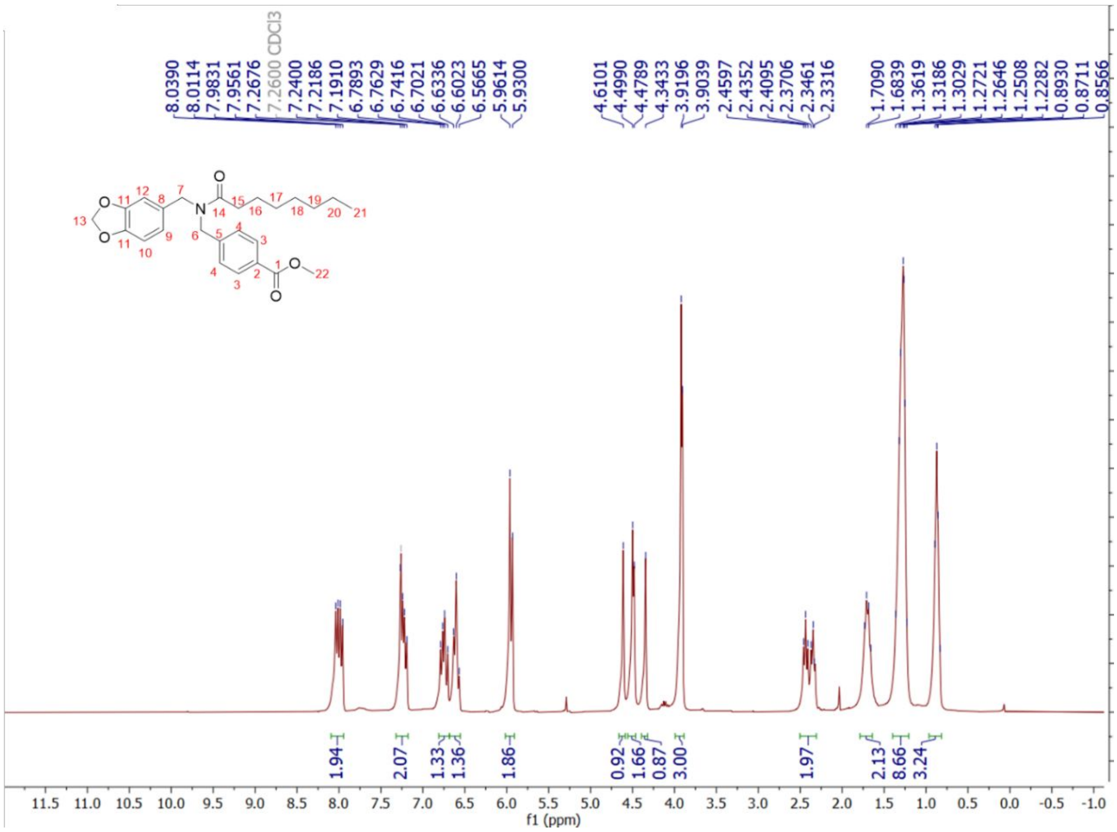

Figure S11. ^{1}H NMR Spectrum of Intermediate **6b** (300 MHz, $CDCl_3$, δ = ppm).

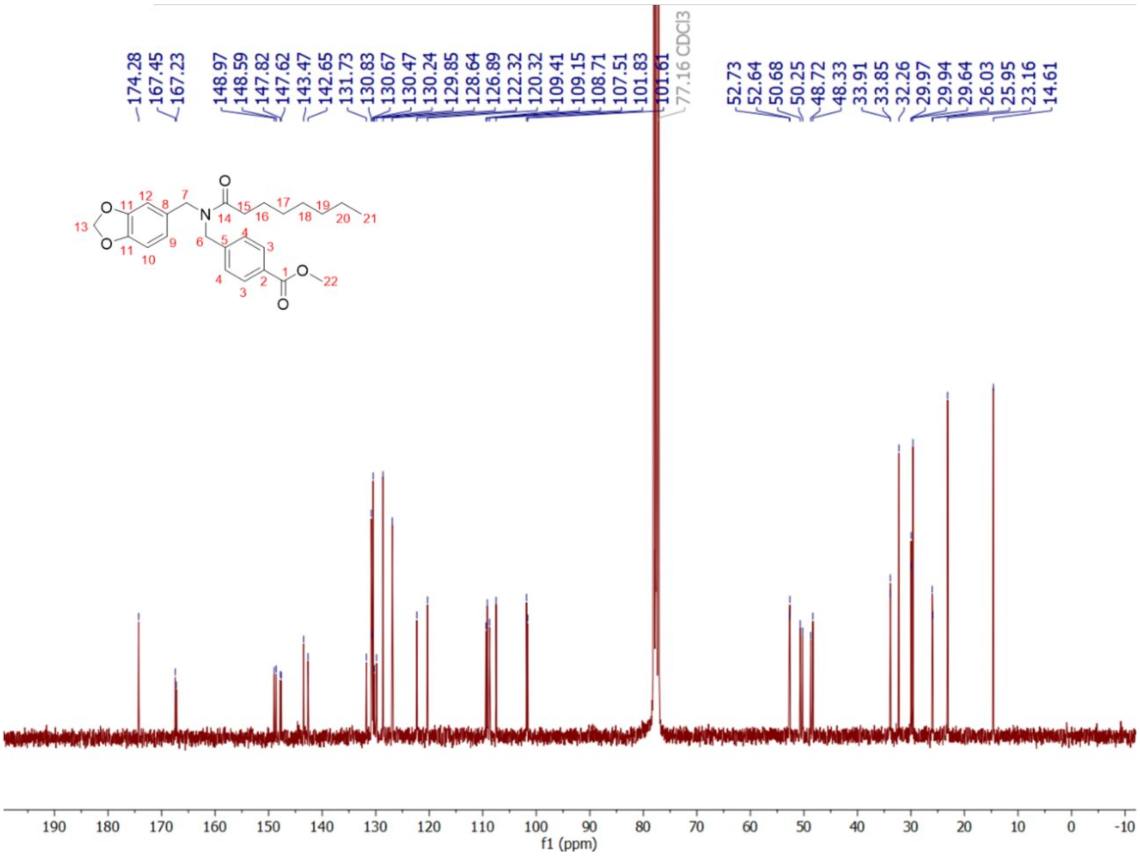

Figure S12. ^{13}C NMR Spectrum of Intermediate **6b** (75 MHz, $CDCl_3$, δ = ppm).

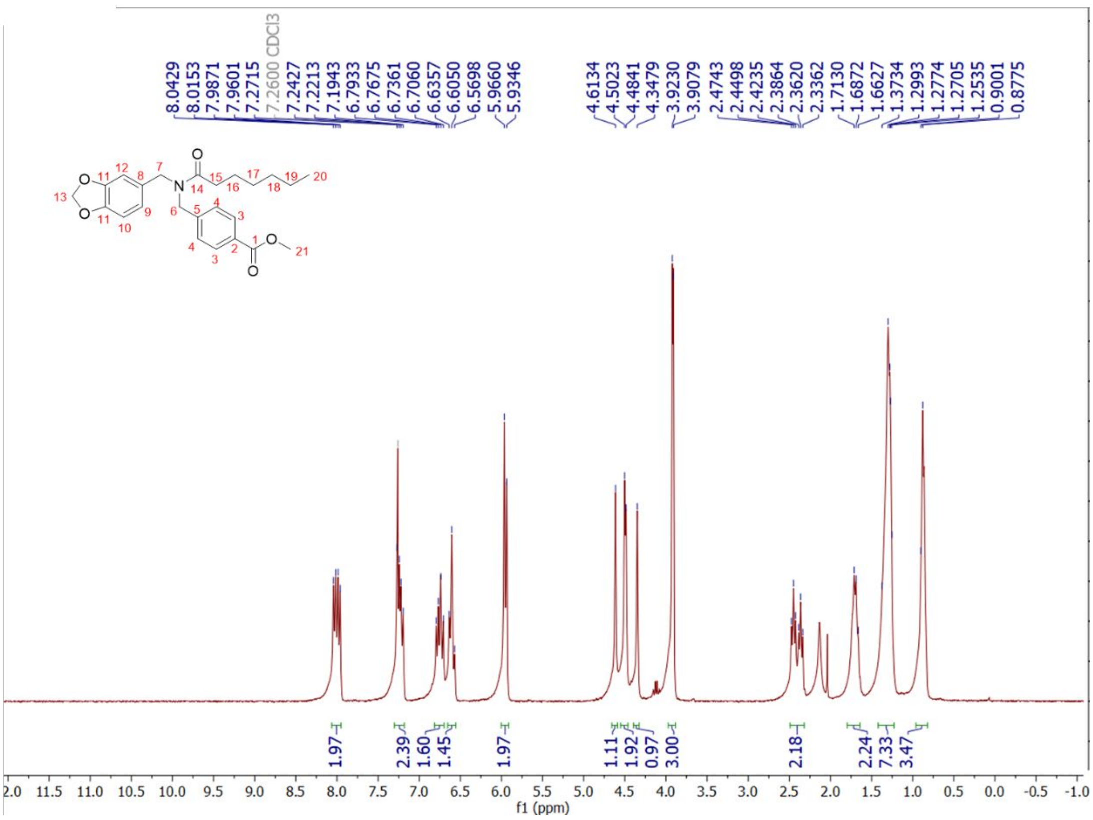

Figure S13. ^{1}H NMR Spectrum of Intermediate **6c** (300 MHz, $CDCl_3$, δ = ppm).

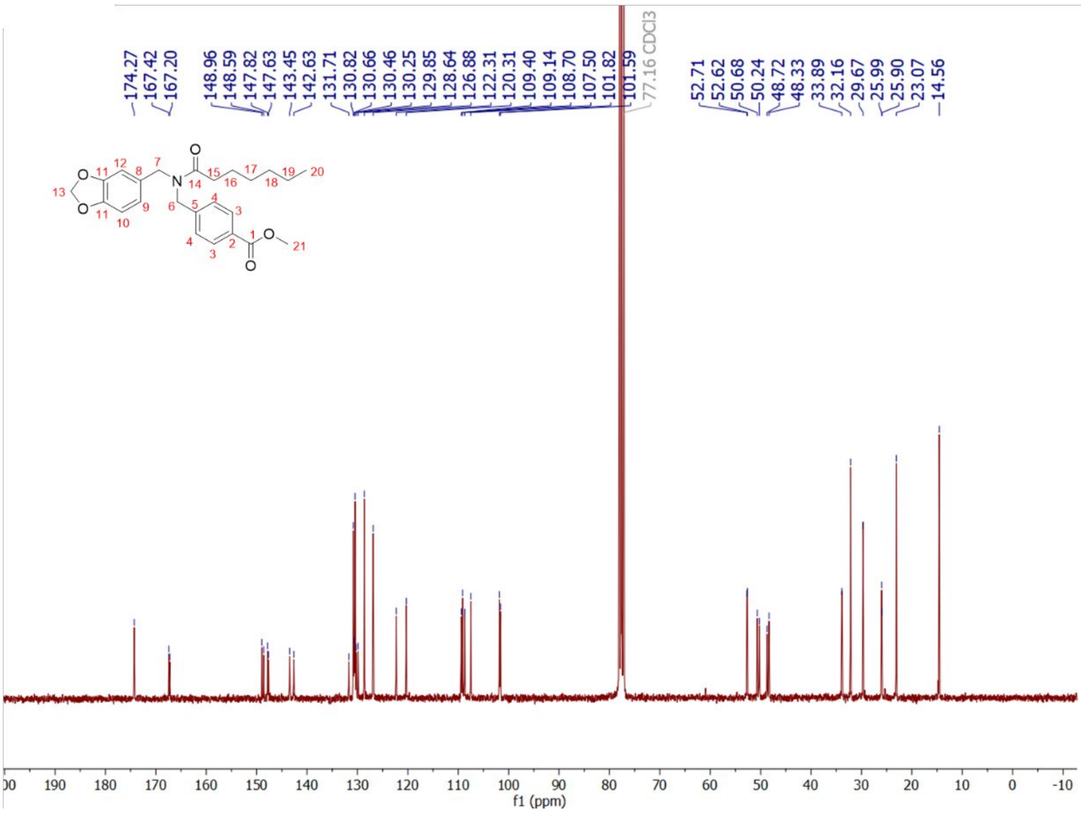

Figure S14. ^{13}C NMR Spectrum of Intermediate **6c** (75 MHz, $CDCl_3$, δ = ppm).

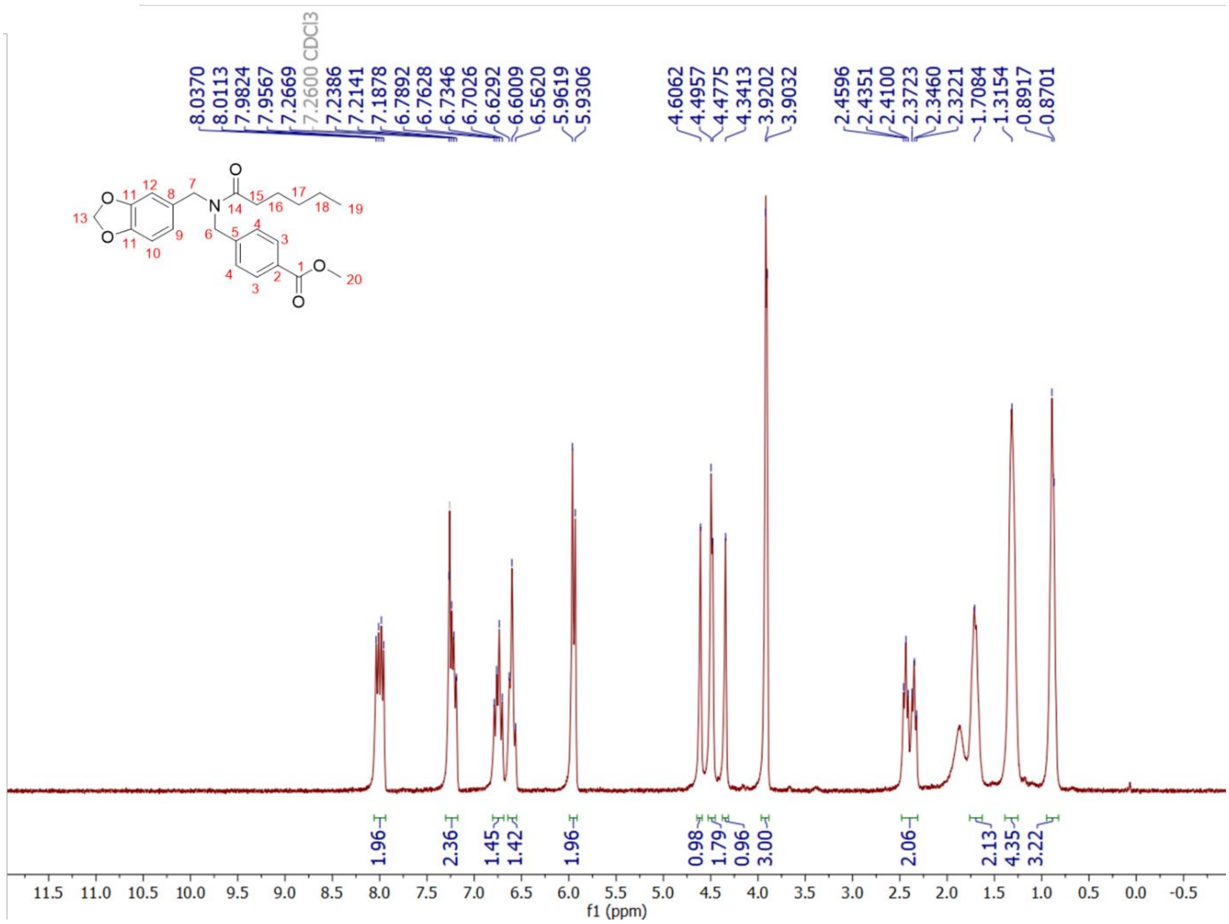

Figure S15. ^{1}H NMR Spectrum of Intermediate **6d** (300 MHz, $CDCl_3$, δ = ppm).

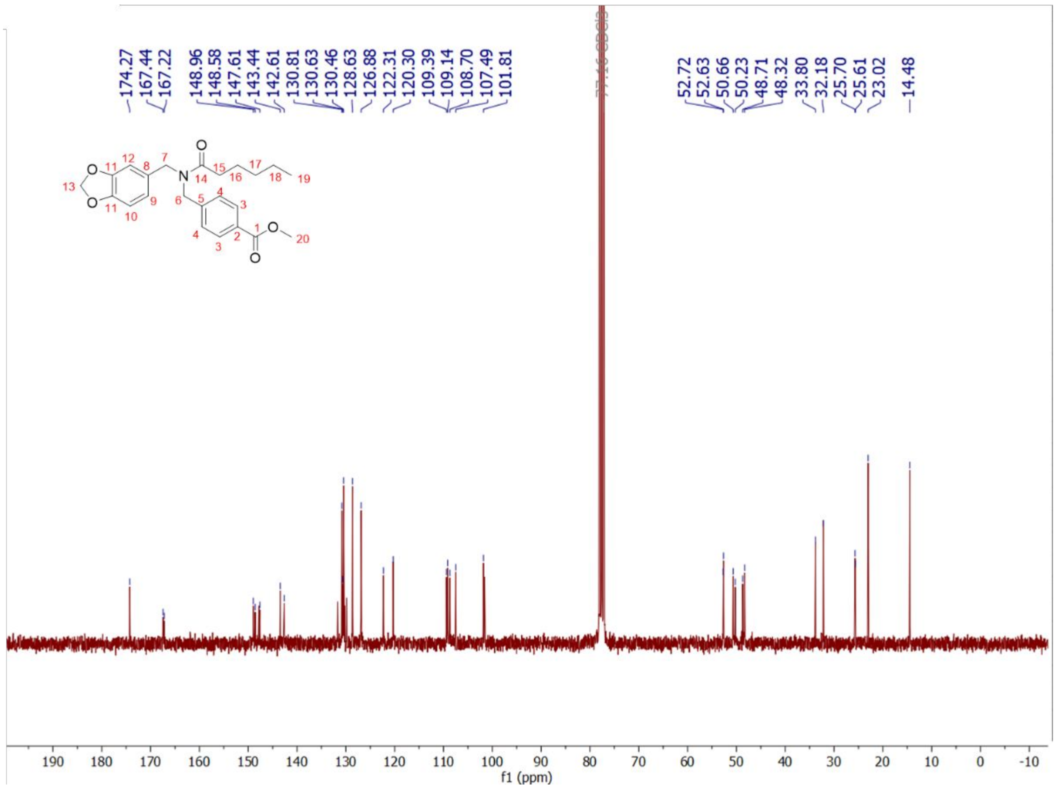

Figure S16. ^{13}C NMR Spectrum of Intermediate **6d** (75 MHz, $CDCl_3$, δ = ppm).

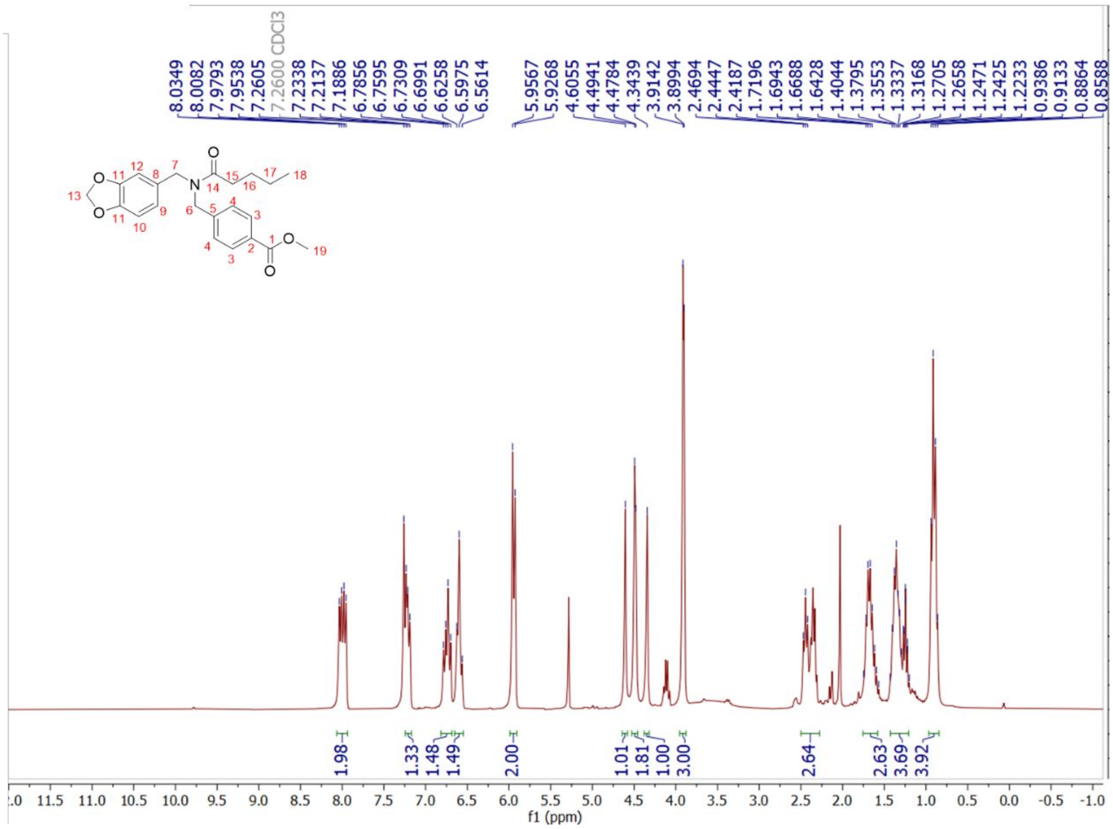

Figure S17. ^{1}H NMR Spectrum of Intermediate **6e** (300 MHz, $CDCl_3$, δ = ppm).

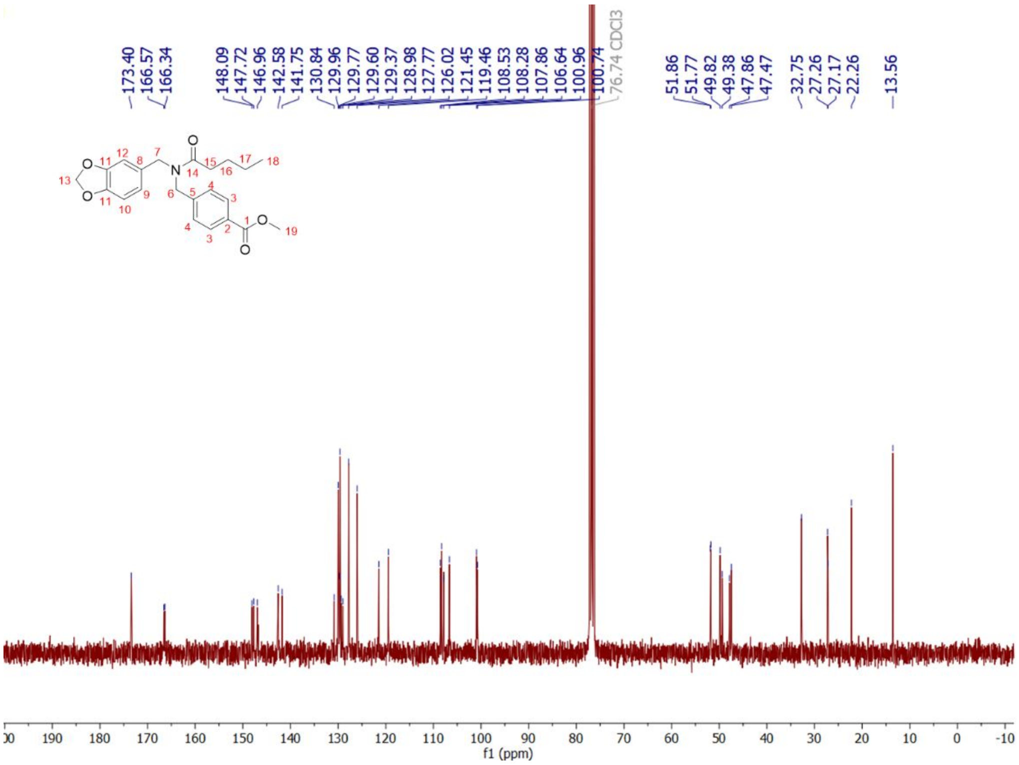

Figure S 18. ^{13}C NMR Spectrum of Intermediate **6e** (75 MHz, $CDCl_3$, δ = ppm).

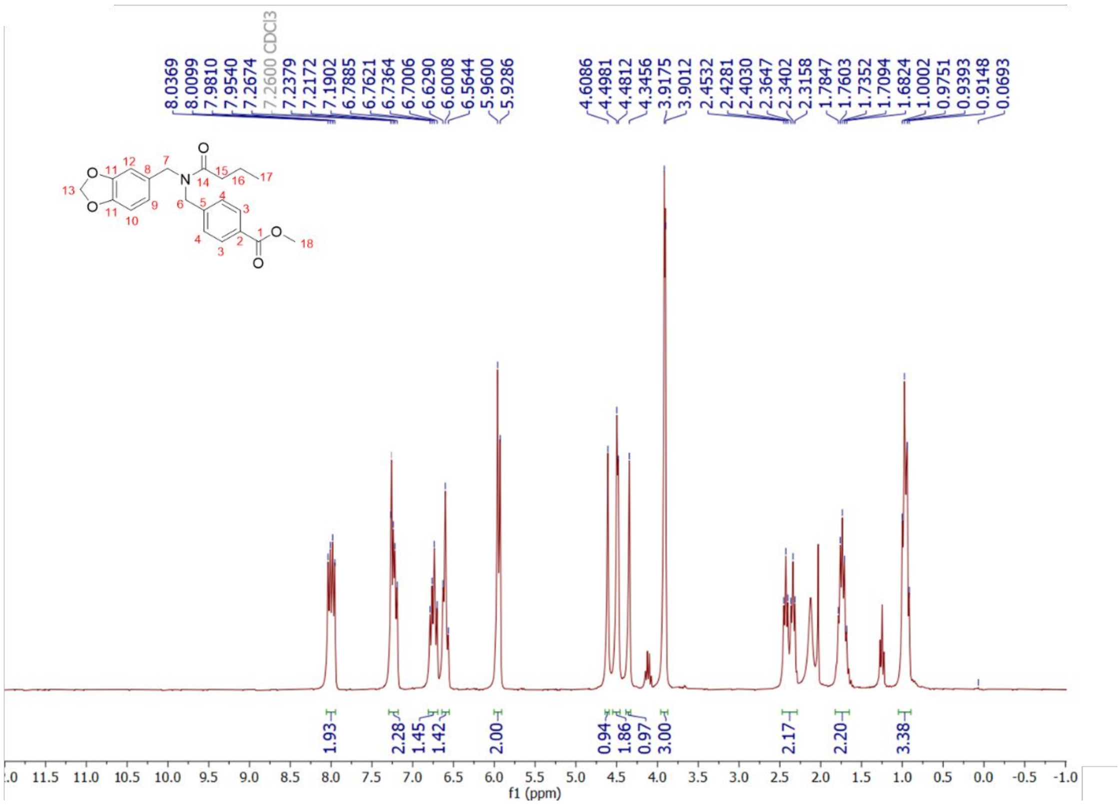

Figure S19. ^{1}H NMR Spectrum of Intermediate **6f** (300 MHz, $CDCl_3$, δ = ppm).

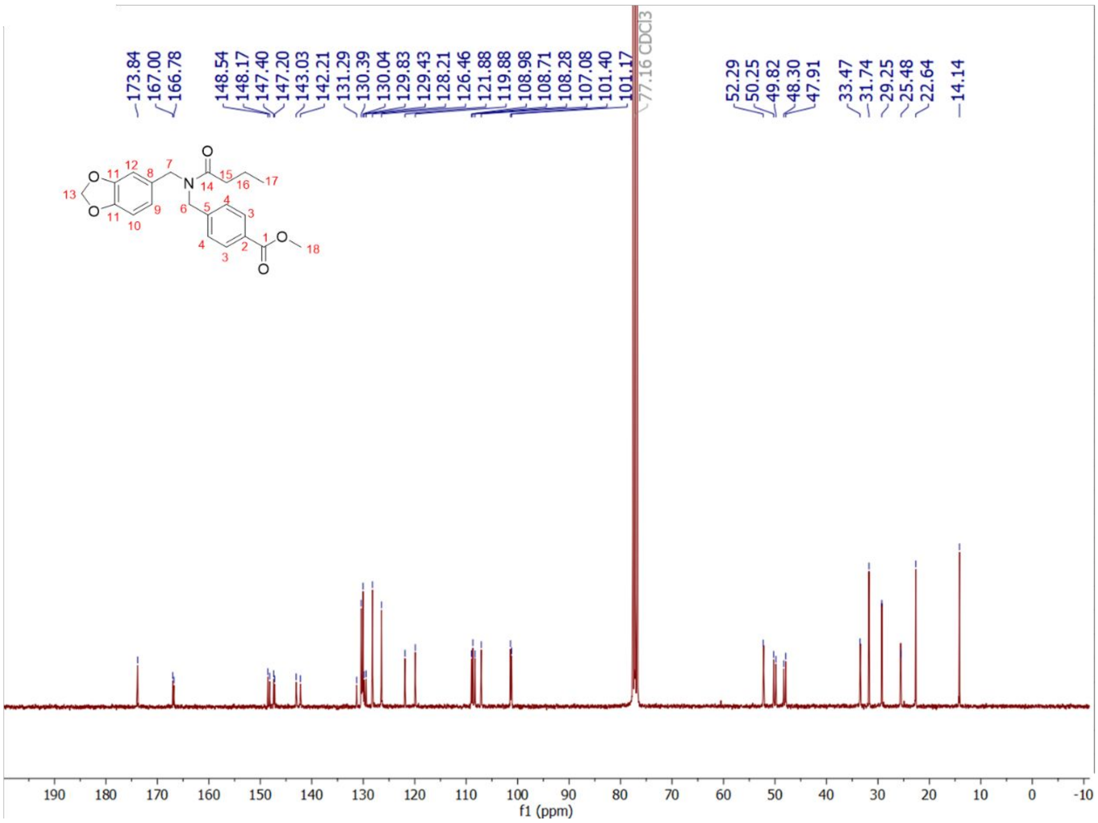

Figure S20. ^{13}C NMR Spectrum of Intermediate **6f** (75 MHz, $CDCl_3$, δ = ppm).

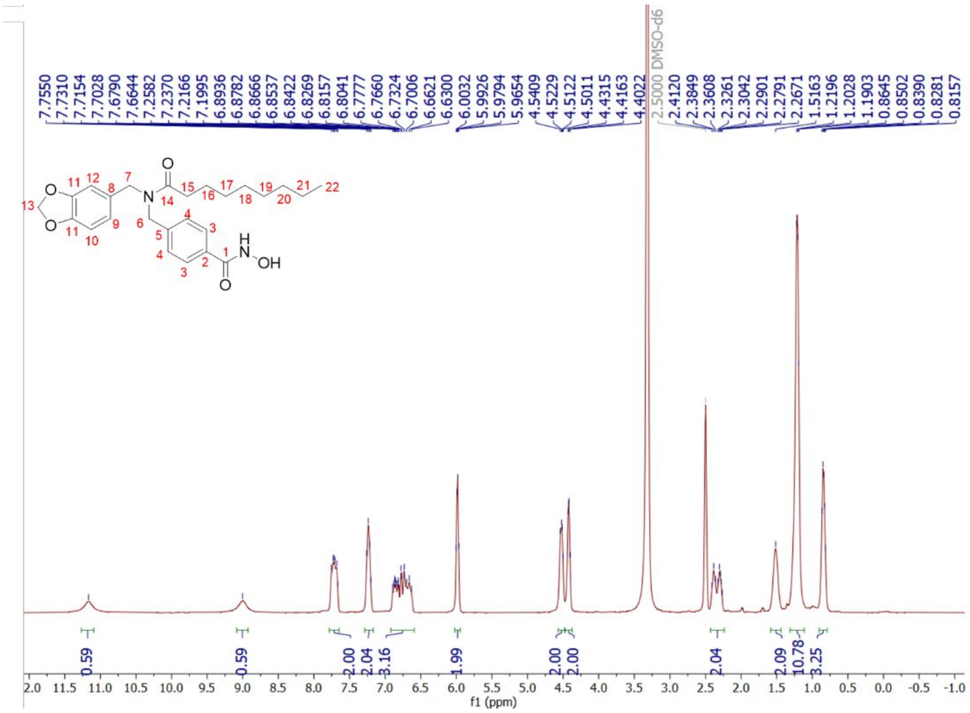

Figure S21. ^{1}H NMR Spectrum of Intermediate **7a** (300MHz, DMSO-d6, δ = ppm).

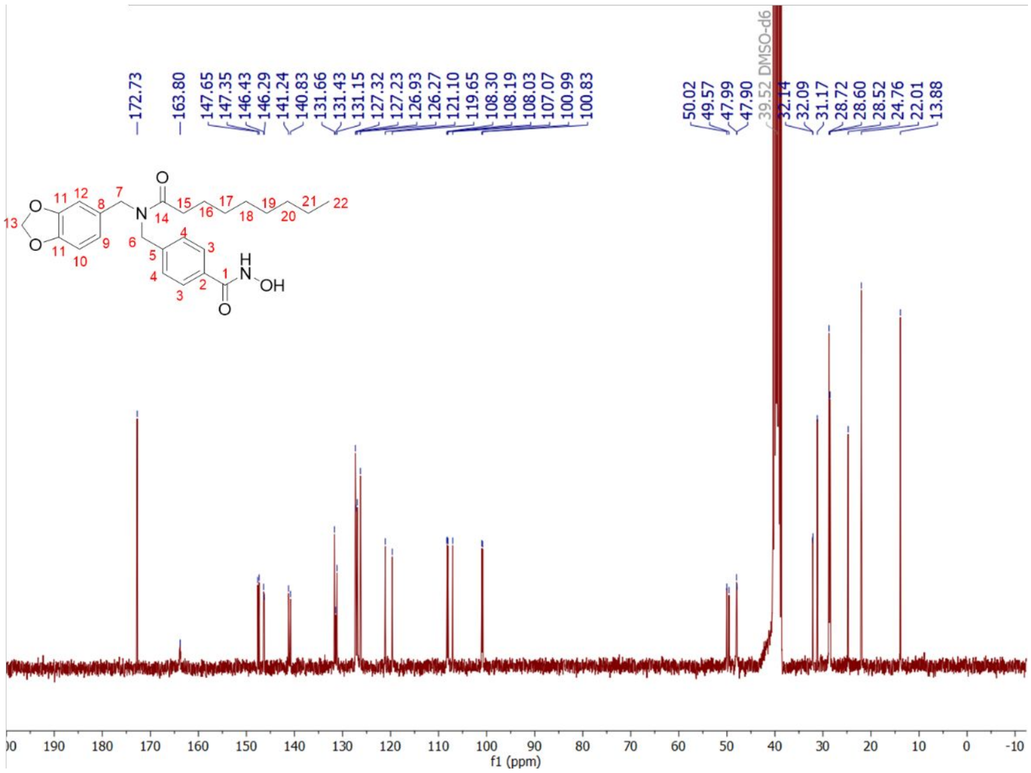

Figure S22. ^{13}C NMR Spectrum of Intermediate **7a** (75 MHz, DMSO-d6, δ = ppm).

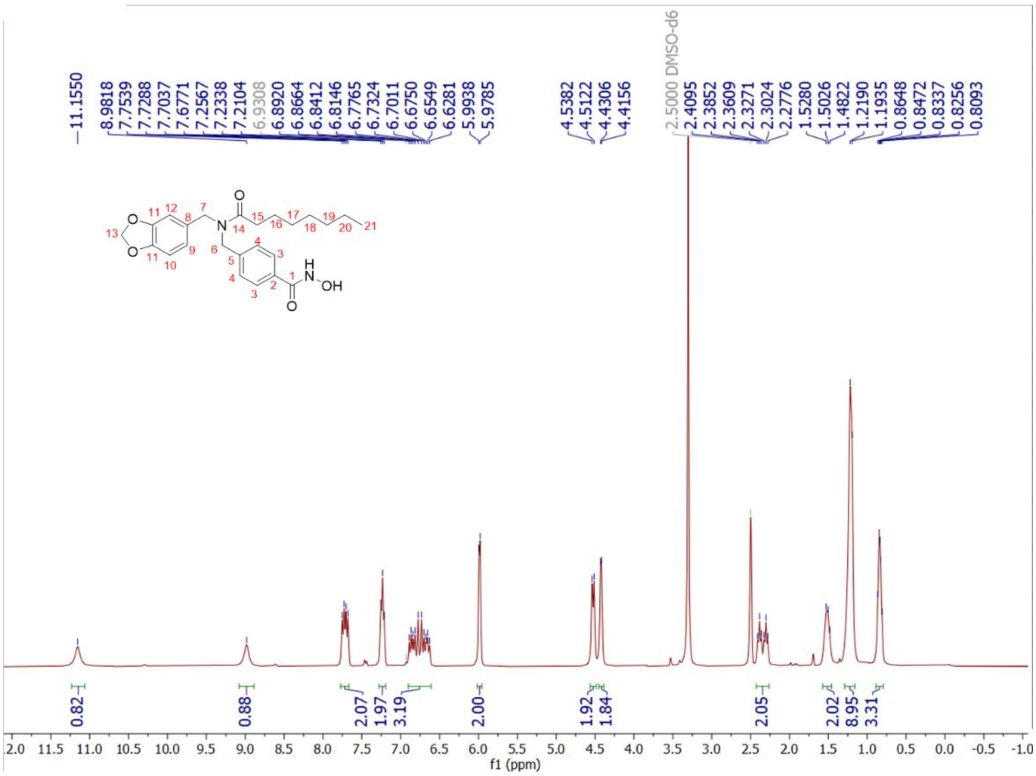

Figure S23. ^{1}H NMR Spectrum of Intermediate **7b** (300 MHz, DMSO-d6, δ = ppm).

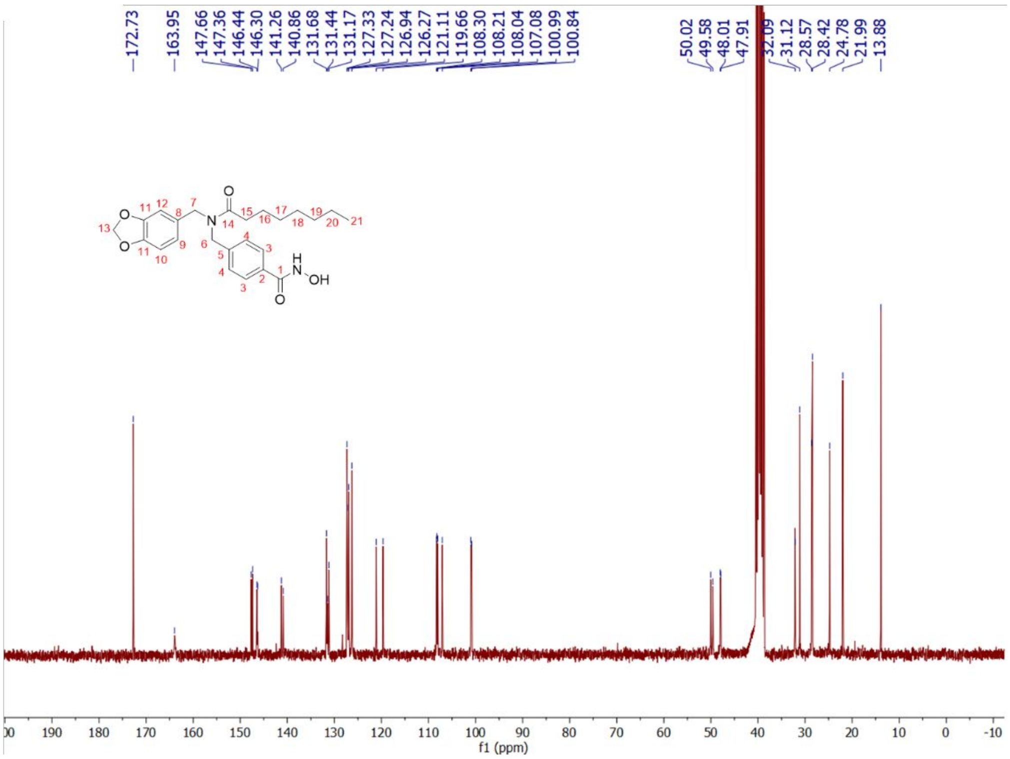

Figure S24. ^{13}C NMR Spectrum of Intermediate **7b** (75 MHz, DMSO-d6, δ = ppm).

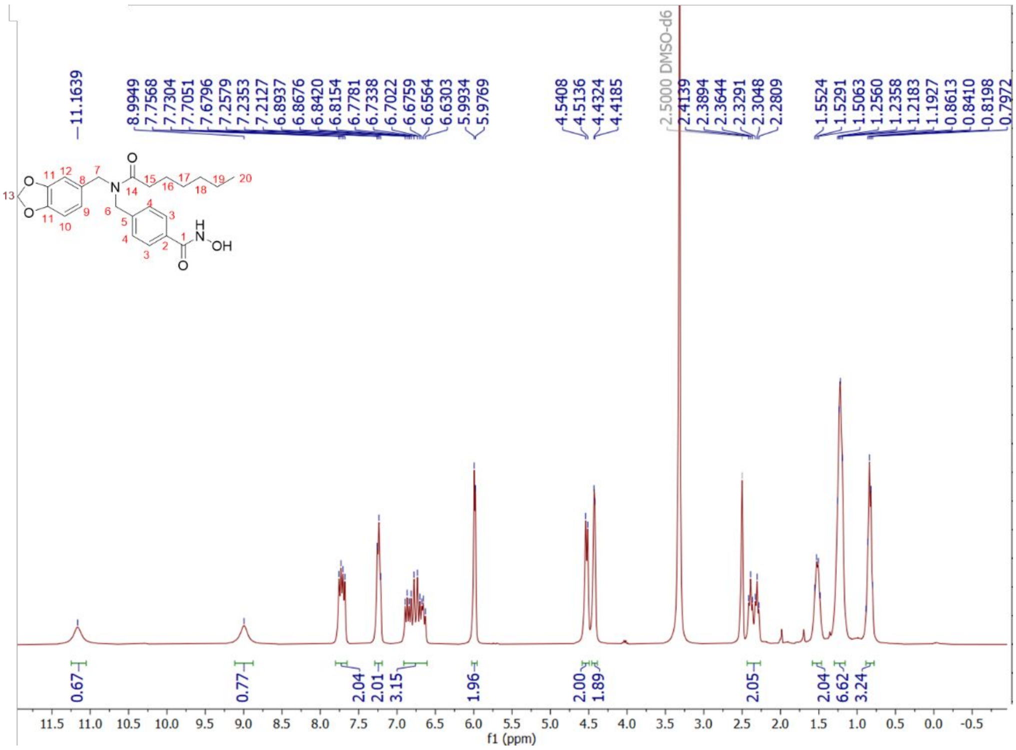

Figure S25. ^{1}H NMR Spectrum of Intermediate **7c** (300 MHz, DMSO-d6, δ = ppm).

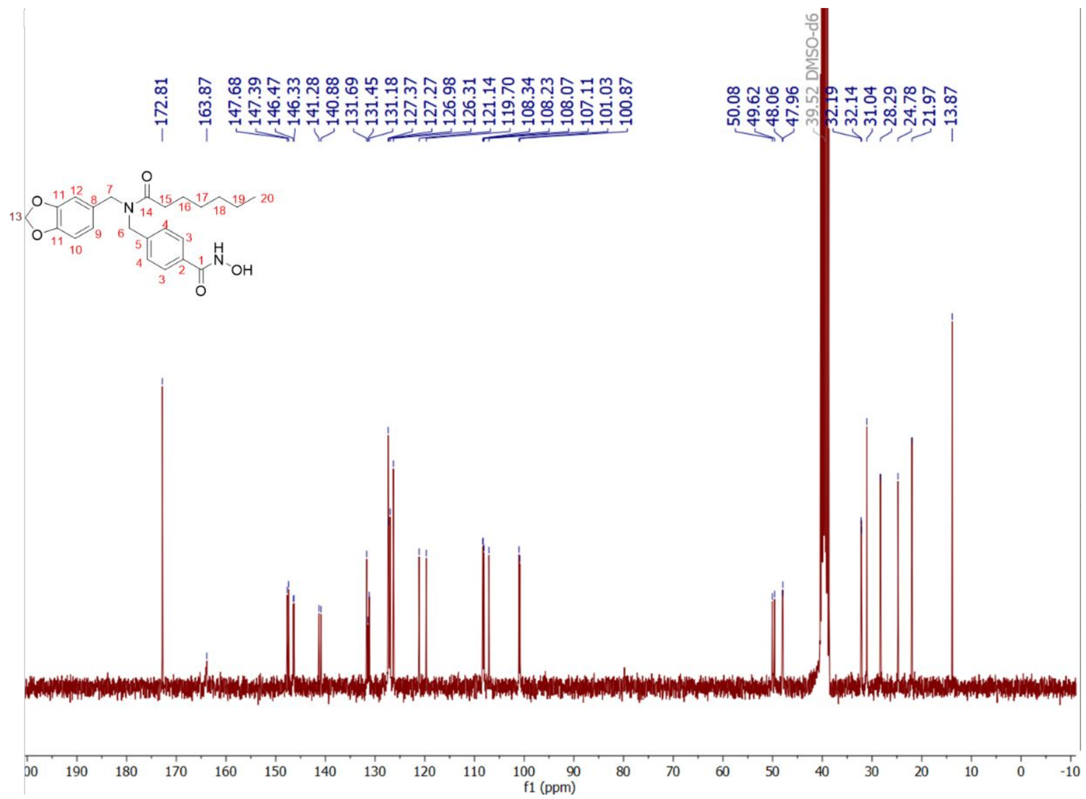

Figure S26. ^{13}C NMR Spectrum of Intermediate **7c** (75 MHz, DMSO-d6, δ = ppm).

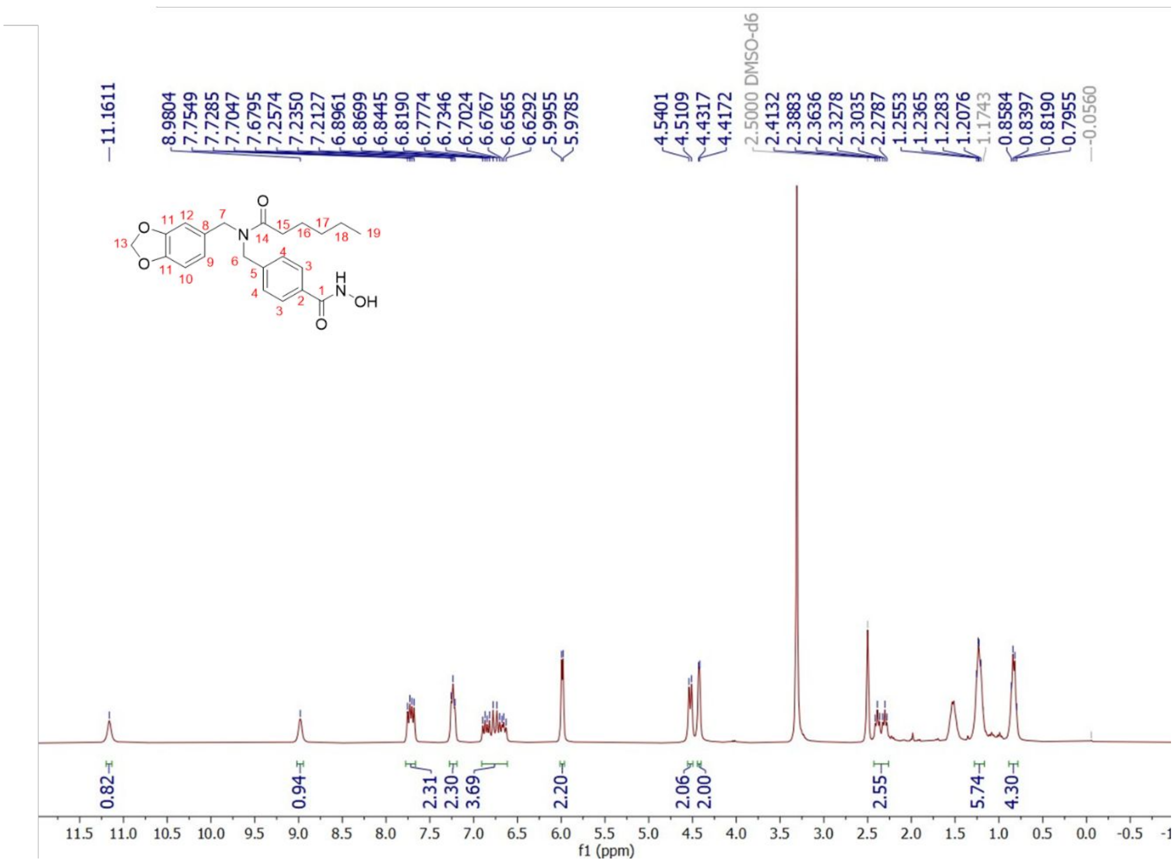

Figure S27. ^{1}H NMR Spectrum of Intermediate **7d** (300 MHz, DMSO-d6, δ = ppm).

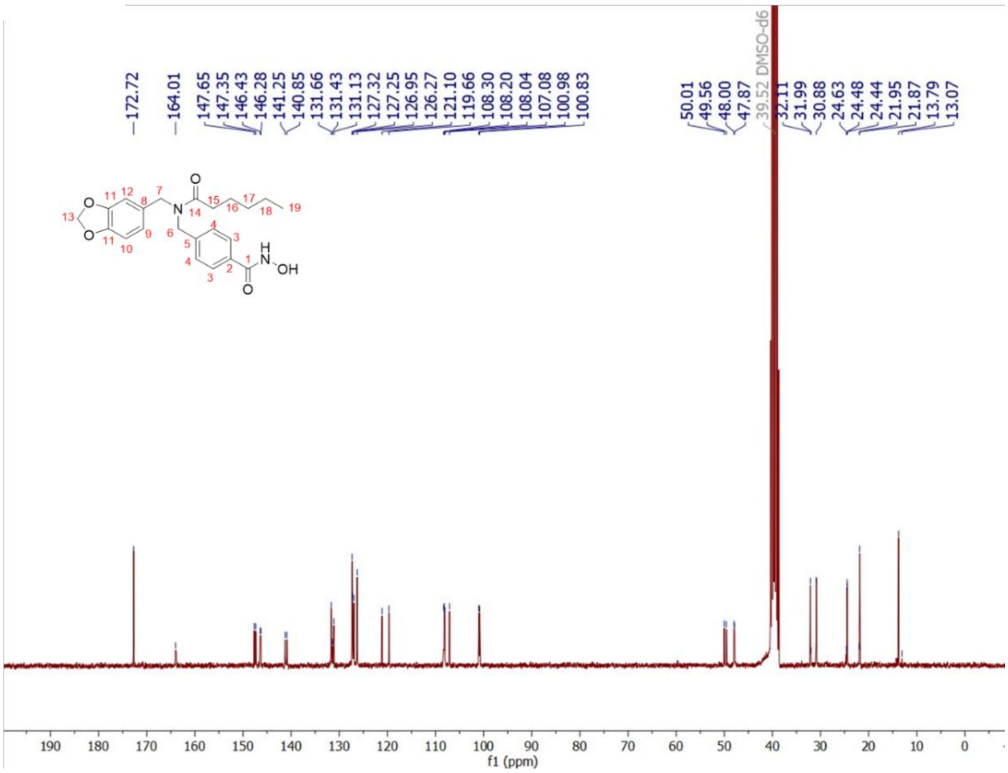

Figure S28. ^{13}C NMR Spectrum of Intermediate **7d** (75 MHz, DMSO-d6, δ = ppm).

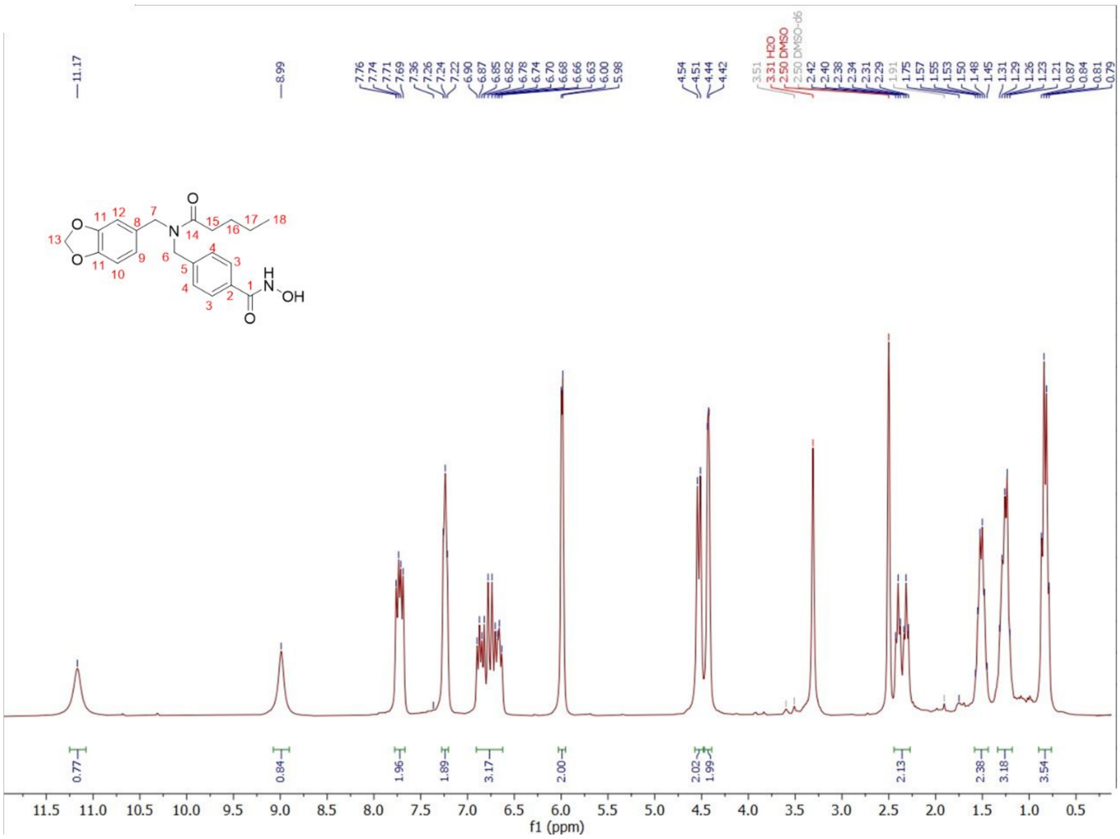

Figure S29. ^{1}H NMR Spectrum of Intermediate **7e** (300 MHz, DMSO-d6, δ = ppm).

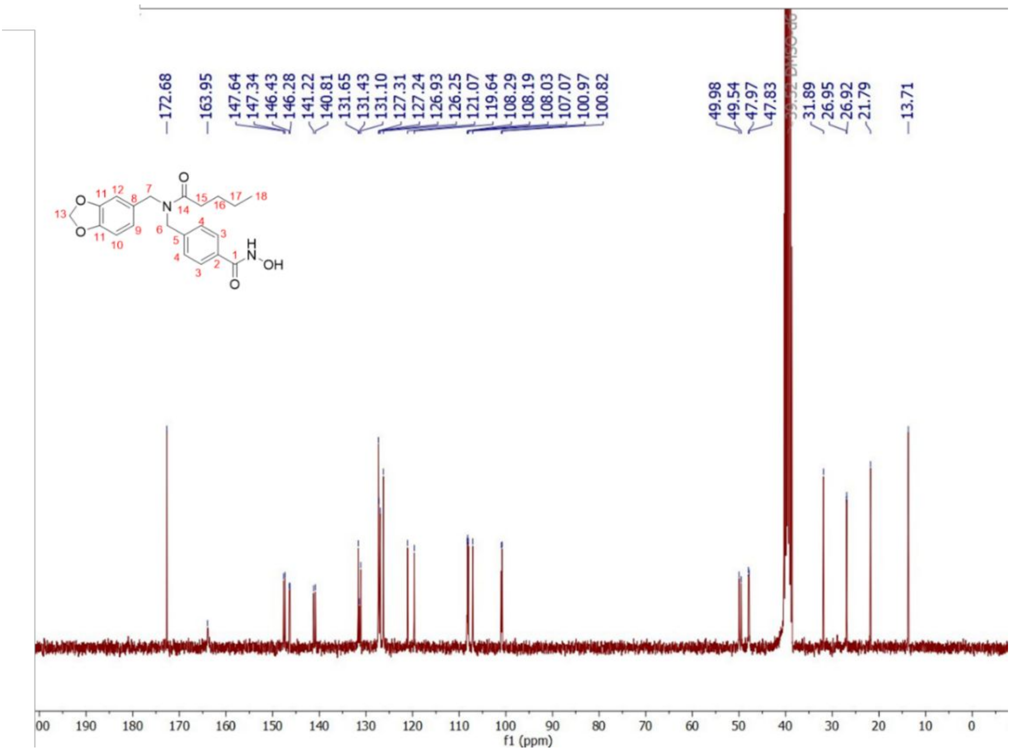

Figure S30. ^{13}C NMR Spectrum of Intermediate **7e** (75 MHz, DMSO-d6, δ = ppm).

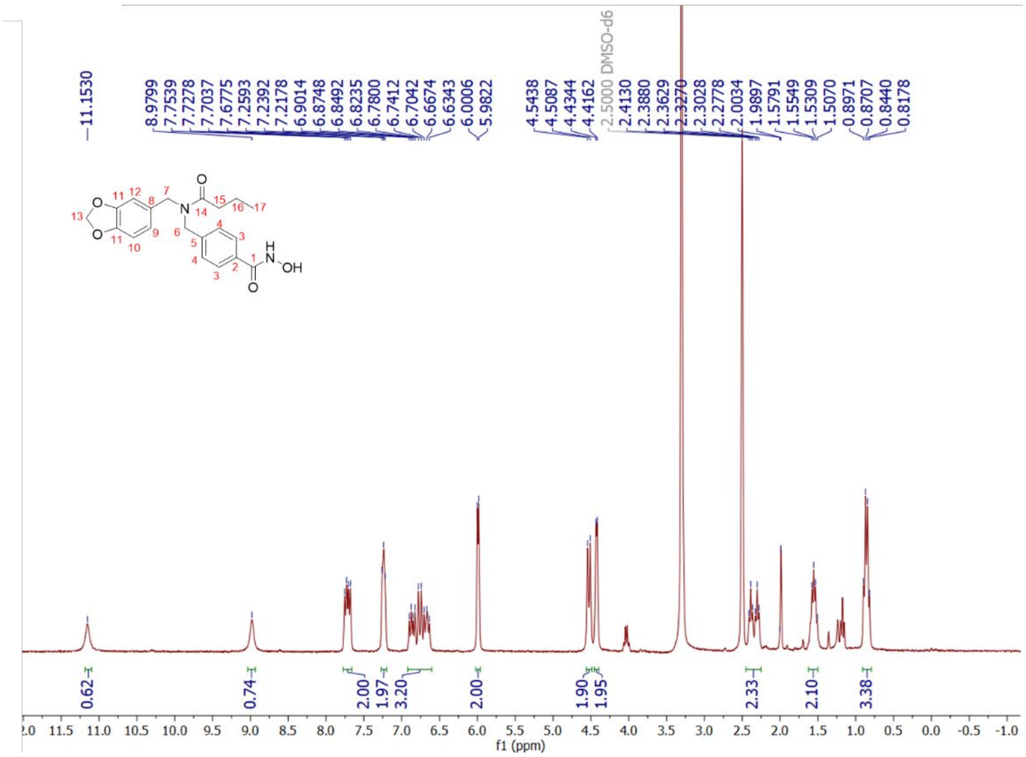

Figure S31. ^{1}H NMR Spectrum of Intermediate **7f** (300 MHz, DMSO-d6, δ = ppm).

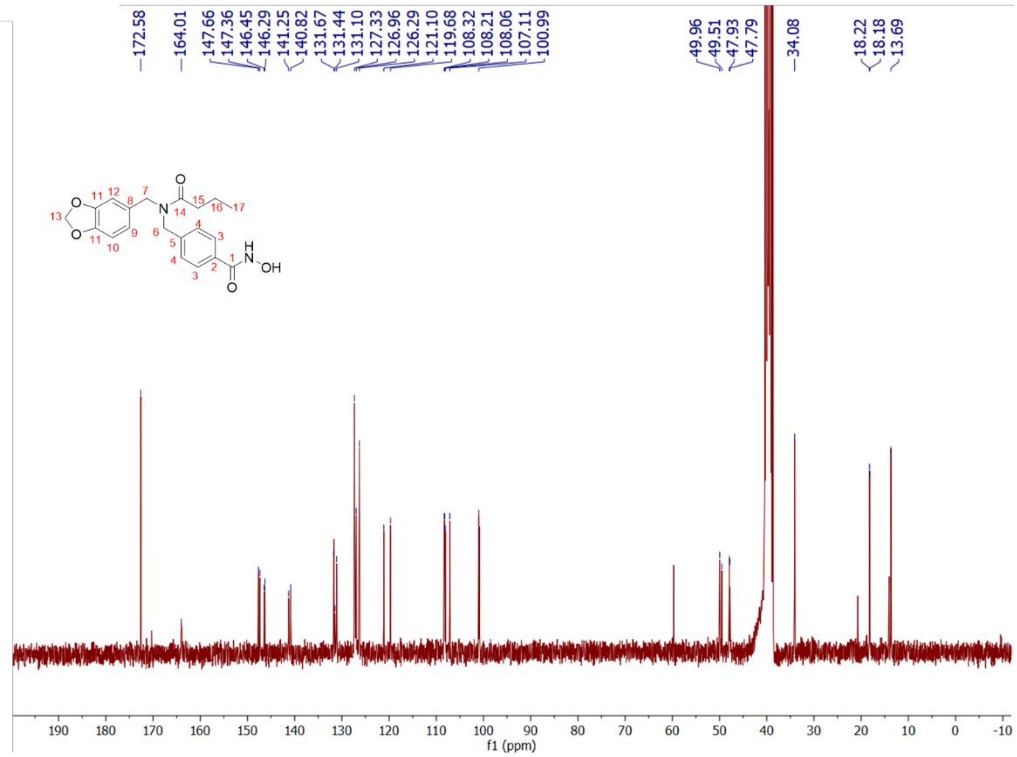

Figure S32. ^{13}C NMR Spectrum of Intermediate **7f** (75 MHz, DMSO-d6 δ = ppm).

Purity Analysis

The purity analyses of the synthesized molecules were conducted at the Analytical Center of the Department of Pharmaceutical Sciences, USP. The analysis was performed using high-performance liquid chromatography (HPLC) with a liquid-solid column system. A Shimadzu Proeminence chromatograph equipped with a Promenex C-18 110 Å analytical column (5 µm, 150 × 4.6 mm) was used.

Samples were diluted in DMSO at a concentration of 0.25 mg/mL. Detection was performed using ultraviolet (UV) light at 254 nm, with an injection volume of 20 µL. The elution system consisted of deionized water and acetonitrile (ACN), both containing 0.1% trifluoroacetic acid (TFA), with a flow rate of 1 mL/min. The chromatographic run started with 5% ACN/TFA and gradually increased to 100% over 20 minutes.

==== Shimadzu LCsolution Analysis Report ====

C:\LabSolutions\Data\Lara\LGB68A_2_16_12_22.lcd

Acquired by	: Admin
Sample Name	: LGB68A_2_16_12_22
Sample ID	: LGB68A_2_16_12_22
Vail #	: 42
Injection Volume	: 20 uL
Data File Name	: LGB68A_2_16_12_22.lcd
Method File Name	: ACN-H2O-0.1TFA-25min.lcm
Batch File Name	:
Report File Name	: relat-Denise.lcr
Data Acquired	: 16/12/2022 10:07:00
Data Processed	: 16/12/2022 10:38:36

<Chromatogram>

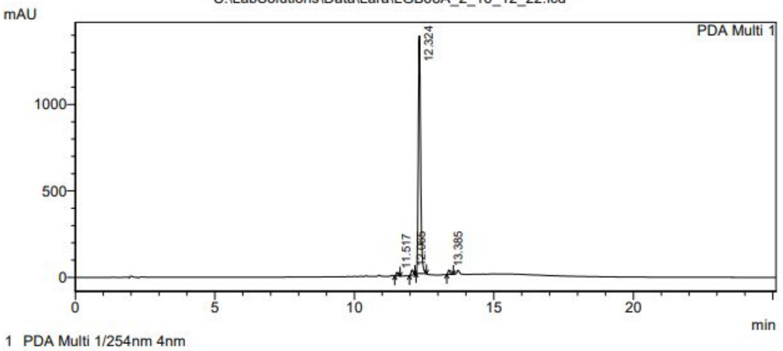

1 PDA Multi 1/254nm 4nm

PeakTable

PDA Ch1 254nm 4nm

Peak#	Name	Ret. Time	Area	Area %
1	RT11.517	11.517	96585	1.174
2	RT12.065	12.065	160095	1.946
3	RT12.324	12.324	7836672	95.277
4	RT13.385	13.385	131813	1.603
Total			8225164	100.000

Purity Spectrum

Figure S33. HPLC Chromatogram of Compound **7a**.

==== Shimadzu LCsolution Analysis Report ====

C:\LabSolutions\Data\Lara\LGB62A 15_12_22.lcd

Acquired by	: Admin
Sample Name	: LGB62A 15_12_22
Sample ID	: LGB62A 15_12_22
Vail #	: 45
Injection Volume	: 20 uL
Data File Name	: LGB62A 15_12_22.lcd
Method File Name	: ACN-H2O-0.1TFA-25min.lcm
Batch File Name	:
Report File Name	: relat-Denise.lcr
Data Acquired	: 15/12/2022 12:47:32
Data Processed	: 15/12/2022 16:38:37

<Chromatogram>

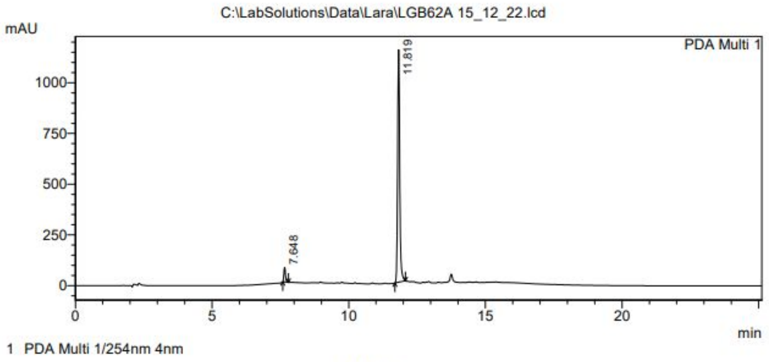

PeakTable

PDA Ch1 254nm 4nm

Peak#	Name	Ret. Time	Area	Area %
1	RT7.648	7.648	299224	4.525
2	RT11.819	11.819	6314134	95.475
Total			6613359	100.000

Purity

Spectrum

Figure S34. HPLC Chromatogram of Compound **7b**.

==== Shimadzu LCsolution Analysis Report ====

C:\LabSolutions\Data\Lara\LGB59A_2_15_12_22.lcd

Acquired by	: Admin
Sample Name	: LGB59A_2_15_12_22
Sample ID	: LGB59A_2_15_12_22
Vail #	: 48
Injection Volume	: 20 uL
Data File Name	: LGB59A_2_15_12_22.lcd
Method File Name	: ACN-H2O-0.1TFA-25min.lcm
Batch File Name	:
Report File Name	: relat-Denise.lcr
Data Acquired	: 15/12/2022 16:07:01
Data Processed	: 16/12/2022 08:32:55

<Chromatogram>

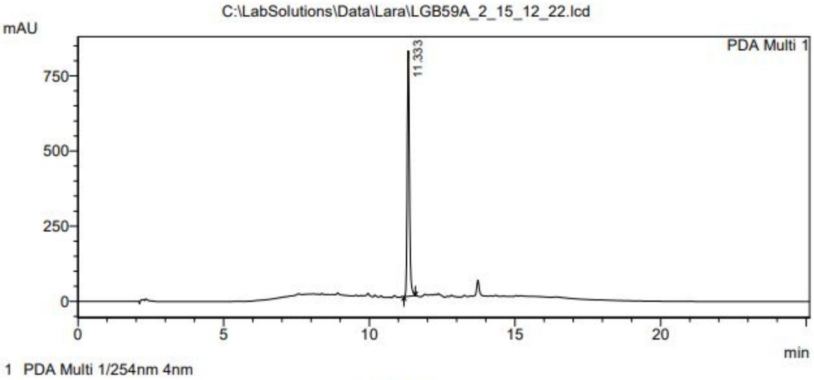

PeakTable

PDA Ch1 254nm 4nm

Peak#	Name	Ret. Time	Area	Area %
1	RT11.333	11.333	4282124	100.000
Total			4282124	100.000

Figure S35. HPLC Chromatogram of Compound **7c**.

==== Shimadzu LCsolution Analysis Report ====

C:\LabSolutions\Data\Lara\LGB57A 15_12_22.lcd

Acquired by : Admin
Sample Name : LGB57A 15_12_22
Sample ID : LGB57A 15_12_22
Vail # : 43
Injection Volume : 20 uL
Data File Name : LGB57A 15_12_22.lcd
Method File Name : ACN-H2O-0.1TFA-25min.lcm
Batch File Name :
Report File Name : relat-Denise.lcr
Data Acquired : 15/12/2022 10:25:45
Data Processed : 15/12/2022 16:35:46

<Chromatogram>

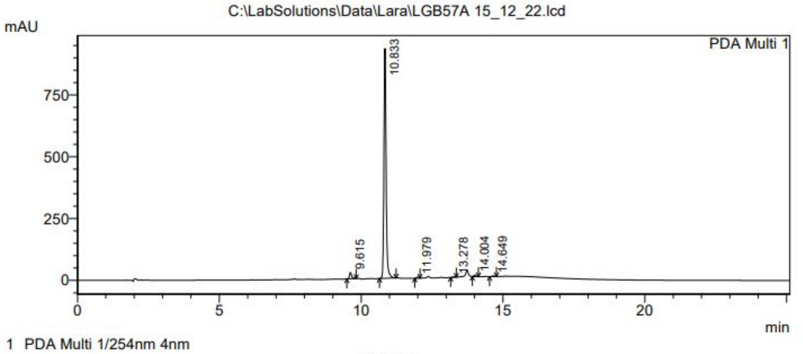

1 PDA Multi 1/254nm 4nm

PeakTable

PDA Ch1 254nm 4nm

Peak#	Name	Ret. Time	Area	Area %
1	RT9.615	9.615	134696	2.625
2	RT10.833	10.833	4925490	95.982
3	RT11.979	11.979	21084	0.411
4	RT13.278	13.278	8708	0.170
5	RT14.004	14.004	26712	0.521
6	RT14.649	14.649	14968	0.292
Total			5131656	100.000

Figure S36. HPLC Chromatogram of Compound **7d**.

==== Shimadzu LCsolution Analysis Report ====

C:\LabSolutions\Data\Lara\LGB58A_2_16_12_22.lcd

Acquired by : Admin
Sample Name : LGB58A_2_16_12_22
Sample ID : LGB58A_2_16_12_22
Vail # : 41
Injection Volume : 20 uL
Data File Name : LGB58A_2_16_12_22.lcd
Method File Name : ACN-H2O-0.1TFA-25min.lcm
Batch File Name :
Report File Name : relat-Denise.lcr
Data Acquired : 16/12/2022 09:39:46
Data Processed : 16/12/2022 10:16:07

<Chromatogram>

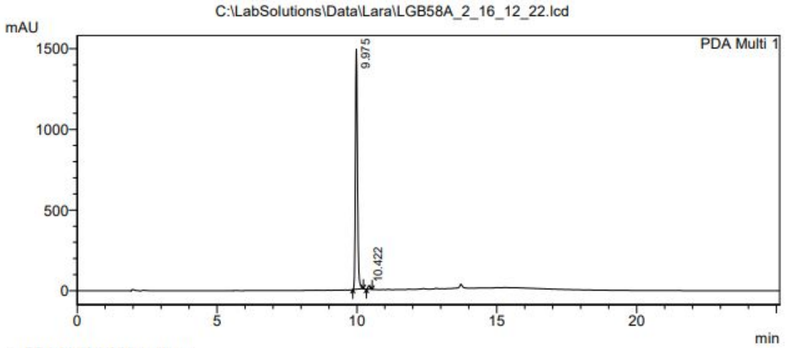

1 PDA Multi 1/254nm 4nm

PeakTable

PDA Ch1 254nm 4nm

Peak#	Name	Ret. Time	Area	Area %
1	RT9.975	9.975	7572983	98.442
2	RT10.422	10.422	119862	1.558
Total			7692845	100.000

Purity Spectrum

Figure S37. HPLC Chromatogram of Compound **7e**.

==== Shimadzu LCsolution Analysis Report ====

C:\LabSolutions\Data\Lara\LGB35A_2_16_12_22.lcd

Acquired by	: Admin
Sample Name	: LGB35A_2_16_12_22
Sample ID	: LGB35A_2_16_12_22
Vail #	: 40
Injection Volume	: 20 uL
Data File Name	: LGB35A_2_16_12_22.lcd
Method File Name	: ACN-H2O-0.1TFA-25min.lcm
Batch File Name	:
Report File Name	: relat-Denise.lcr
Data Acquired	: 16/12/2022 09:12:36
Data Processed	: 16/12/2022 10:19:50

<Chromatogram>

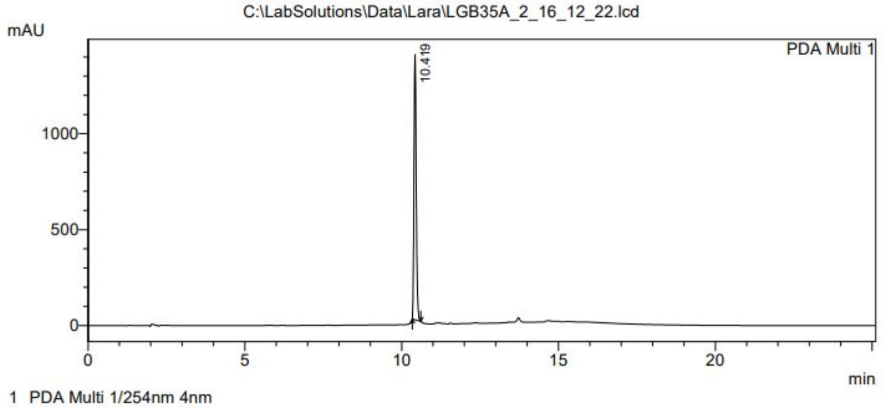

1 PDA Multi 1/254nm 4nm

PeakTable

PDA Ch1 254nm 4nm

Peak#	Name	Ret. Time	Area	Area %
1	RT10.419	10.419	7008990	100.000
Total			7008990	100.000

Figure S38. HPLC Chromatogram of Compound **7f**.

==== Shimadzu LCsolution Analysis Report ====

C:\LabSolutions\Data\Lara\Branco_3_16_12_22.lcd

Acquired by	: Admin
Sample Name	: Branco_3_16_12_22
Sample ID	: Branco_3_16_12_22
Vail #	: 44
Injection Volume	: 20 uL
Data File Name	: Branco_3_16_12_22.lcd
Method File Name	: ACN-H2O-0.1TFA-25min.lcm
Batch File Name	:
Report File Name	: relat-Denise.lcr
Data Acquired	: 16/12/2022 11:11:24
Data Processed	: 16/12/2022 11:36:33

<Chromatogram>

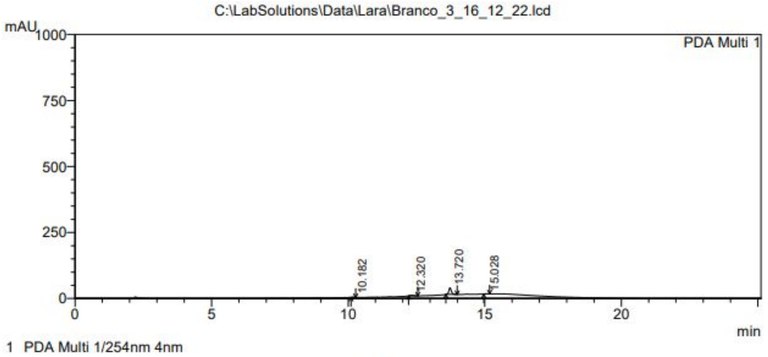

1 PDA Multi 1/254nm 4nm

PeakTable

PDA Ch1 254nm 4nm

Peak#	Name	Ret. Time	Area	Area %
1	RT10.182	10.182	5160	2.677
2	RT12.320	12.320	18510	9.601
3	RT13.720	13.720	164487	85.318
4	RT15.028	15.028	4637	2.405
Total			192793	100.000

Figure S39. HPLC Chromatogram of the Blank sample.
